# Environmentally friendly inhaler decision-making with personalized care in asthma and chronic obstructive pulmonary disease: a qualitative study

**DOI:** 10.1016/j.rcsop.2025.100651

**Published:** 2025-08-29

**Authors:** Claire D. Visser, Alan Sulaiman, Narrin Bakr, Henk-Jan Guchelaar, Martina Teichert

**Affiliations:** a Department of Clinical Pharmacy and Toxicology, Leiden University Medical Center, Leiden, the Netherlands; b Department of Public Health and Primary Care, Leiden University Medical Center, Leiden, the Netherlands; c Department of Research and Development, Royal Dutch Pharmacists Association, The Hague, the Netherlands

**Keywords:** Public health, Carbon footprint, Inhalers, Hydrofluorocarbon (HFC), Asthma, Chronic obstructive pulmonary disease (COPD), Quality of care

## Abstract

**Background:**

Green behavioral shifts in prescribing habits, device selection and patient counseling have been proposed to deliver low-carbon sustainable healthcare, including the transition from pressurized metered-dose inhalers (pMDIs) to propellant-free inhalers.

**Objective:**

This study explores the extent to which healthcare professionals (HCPs) and patients can factor the environmental impact into inhaler decision-making with personalized care in asthma and chronic obstructive pulmonary disease (COPD).

**Methods:**

An exploratory qualitative study was conducted involving seven focus groups and one semi-structured interview with 22 participants consisting of general practitioners, community pharmacists, pulmonologists, assistants and patients. Data was analyzed deductively to identify challenges and facilitators related to capability, opportunity and motivation; and inductively for actions to foster environmentally conscious behavior.

**Results:**

Overall, participants felt a moral responsibility and were willing to opt for environmentally friendly inhaler initiatives including a shift from pMDIs to dry-powder inhalers (DPIs), if clinically appropriate and performed as outcome of shared decision-making. Collaboration between researchers and relevant stakeholders was required to strengthen their capacity to advance in this area. Proposed strategies covered five areas: (1) communication, education and awareness; (2) appropriate inhaler prescribing; (3) promotion of smarter inhaler choices; (4) optimization of quality of care; and (5) appropriate inhaler disposal.

**Conclusion:**

These findings guide the delivery of a range of opportunities to improve quality of care while simultaneously reduce carbon footprint. This requires a multifactorial and interdisciplinary approach with HCPs playing a central role in engaging and educating patients to determine the viability of environmentally friendly alternatives, promote correct inhaler use and appropriate disposal.

## 1 Introduction

Climate change is one of the greatest most pervasive health threats worldwide resulting from anthropogenic factors, in particular greenhouse gas (GHG) emissions.^1^ Asthma and chronic obstructive pulmonary disease (COPD) are highly prevalent non-communicable respiratory diseases facing the effects of the environmental footprint. Increasing temperatures, relative humidity and air pollution levels have been associated with accelerated exacerbation, morbidity and mortality rates.^2^ Inhalation therapy is the cornerstone of long-term treatment for these patients, vital for conveying medication directly to target sites in the airways.^3^^,^^4^ Yet, GHG emissions related to inhaler devices contribute to climate change, promoting or exacerbating existing respiratory diseases, necessitating further inhaler prescribing.^5^^,^^6^

Three principle inhaler device types globally available are pressurized metered-dose inhalers (pMDIs), dry-powder inhalers (DPIs), and soft-mist inhalers (SMIs).^3^^,^^4^ The carbon footprint of these inhaler devices include GHG emissions throughout the entire life cycle under which the manufacturing, transportation, and disposal phase. However, environmental concerns are especially related to the significant burden of hydrofluorocarbon (HFC) propellants in currently used pMDIs, which are predominantly released with dosing. These HFCs (HFC-134a and HFC-227ea) are potent GHGs which form the driving force to generate aerosol clouds containing medication for lung deposition.^7^ In contrast, the alternative options are propellant-free since DPIs rely on patients' inspiratory flow to disaggregate and disperse the powder and SMIs utilize a spring for the dispersion of aqueous medication into the airways.^8^^,^^9^ Specifically, currently available pMDIs have a carbon footprint 20–30 times higher compared to DPIs and SMIs per dose equivalent.^10^ Annually, 630 million pMDIs are used globally for respiratory conditions, accounting for an estimated 13 million tons of CO_2_ equivalent emissions, comparable to the carbon footprint of 2 million European citizens.^11^ Moreover, the demand for pMDIs is expected to grow with increasing population and disease prevalence. It is therefore critical to mitigate GHG emissions resulting from pMDIs, complementing the HFC phase-down under Kigali Amendment of the Montreal Protocol (2016).^12^^,^^13^

Recently, there have been increasing calls to shift away from pMDI prescriptions and encourage DPIs or SMIs as greener inhaler options, targeting the carbon footprint of high-emission inhalers.^14^^,^^15^ While the different inhaler device types offer equivalent clinical effectiveness, they vary significantly with regard to their design and inhaler technique.^16^^,^^17^ Suboptimal matching of inhaler devices with patients can therefore have profound implications, leading to incorrect inhaler use, reduced patient satisfaction, and medication non-adherence.^18^ Evidently, (inter)national guidelines advocate a tailored multi-faceted approach in choosing the most suitable inhaler for each patient, to maximize treatment outcomes.^4^^,^^5^ Moreover, the transition from pMDIs to DPIs has been associated with increased healthcare utilization, raising concerns on potential adverse outcomes and the environmental benefits.^19^ Accordingly, these findings highlight the need to re-evaluate this policy change. Although healthcare professionals (HCPs) are well positioned to facilitate local- and system-level behavior change in practices and organizations,^4^^,^^5^ the extent to which environmentally friendly actions can be incorporated, among the many factors weighed in the complex shared decision-making (SDM) of inhalers, remains uncertain.

Hence, this study aims to explore the perspectives of HCPs and patients on factoring environmental impact into inhaler decision-making with personalized care in asthma and COPD; and reveal actions to promote environmentally conscious behavior in clinical practice.

## 2 Methods

### 2.1 Study design

An exploratory qualitative study was conducted in the Netherlands to provide insights into challenges, facilitators and strategies to implement the environmental impact into inhaler treatment decision-making. This involved focus groups to augment an in-depth understanding of perspectives, attitudes, beliefs, and experiences among primary HCPs (general practitioners (GPs), community pharmacists (CPs), and assistants), secondary HCPs (pulmonologists), and patients (asthma/COPD). Focus groups were organized for each healthcare profession and patients separately to generate a diverse range of information-rich sources.^20^ A flowchart of the study procedures is outlined in appendix A (p 2–3).

The Medical Ethics Committee (MEC) of Leiden University Medical Center (LUMC) declared that the Medical Research Involving Human Subjects Act did not apply to this study. Approval was obtained from the scientific review committee of the department of Clinical Pharmacy and Toxicology of LUMC. The study was conducted in accordance with the Declaration of Helsinki. All HCPs and patients provided written informed consent prior to participation. The ‘Standards for Reporting Qualitative Research’ checklist was followed in the reporting.^21^

### 2.2 Study setting

In the Dutch healthcare system, asthma and COPD patients are predominantly diagnosed and managed by primary HCPs. Community pharmacies and general practices may be organized independently or as part of a franchise or group and may be located in a healthcare center, often sharing a building with multiple HCPs. Difficult to manage respiratory disease patients who require more extensive assessment are often referred to pulmonologists for specialized care in outpatient or hospital settings.^22^, ^23^, ^24^, ^25^ All Dutch citizens have mandatory basic health insurance to cover their primary care needs including GP and hospital visits, specialist and pharmaceutical care, and medical devices.^26^

The majority of prescribed inhaler device types integrated in primary healthcare practices constitute pMDIs and DPIs, as recommended by Dutch medical guidelines. According to the Dutch Foundation of Pharmaceutical Statistics (SFK), who collect medication dispensing data from more than 98 % of approximately 2000 community pharmacies, 1.5 million patients received inhaler medication in year 2022; 51 % as pMDI, 37 % as DPI and 12 % a combination of both inhaler device types. The total number of delivered inhaler medication covered 297 million defined daily dosages (DDDs); 121 million DDD as pMDI and 176 million DDD as DPI. Approximately 60 % of pMDI-using patients received an accompanying spacer or valved holding chamber (VHC). While acknowledging the environmental impact differences of inhalers in Dutch guidelines, an increase in pMDI and decrease in DPI dispensing was observed from 2012 to 2022.^14^^,^^26^

### 2.3 Study participants

HCPs were initially recruited from the Special Interest Group (SIG) Lung associated with the Royal Dutch Pharmacists Association (KNMP), Leiden Academic Network of Pharmacists (LANA) and personal network of the research team. Following, snowball sampling was applied to access hard-to-reach HCPs and enhance participation through social connections. Whereas, patients ≥18 years diagnosed with asthma and/or COPD who received inhaler dispenses in the prior three months (October–December 2022), verified in the electronic pharmacy information system with Anatomical Therapeutic Chemical (ATC) codes for medication treating respiratory diseases (R03), were selected and recruited from four community pharmacies situated across the center and West of the Netherlands.^27^ Purposive sampling ensured the inclusion of patients with various inhaler device types, medication and age groups to provide a comprehensive understanding of inhaler use and its related factors. Additionally, patients were recruited through non-profit lung health organizations and patients' associations using flyers.

Exclusion criteria consisted of cognitive impairments, a lack of digital skills and/or internet access, and non-Dutch speakers. Individuals willing to participate received an information leaflet prior to signing informed consent. No incentives were provided for participation.

### 2.4 Data collection

Focus groups were performed virtually on Microsoft Teams to include geographically dispersed participants with various schedules from January to April 2023. Each focus group was conducted by a moderator (assistant), alternated by three research members (CV, AS, NB). A topic guide with open-ended questions was developed collaboratively by these research members to elicit and encourage participants to provide overt information in an open dialogue approach (appendix B p 4–7). Formulated questions were based on empirical evidence acquired by a systematic literature review regarding patient-related, device-related, and environmentally-related factors influencing inhaler decision-making (appendix C p 8–9 for the applied search strategy). Demographic data were collected at the introductory phase of focus group discussions. A mock focus group was performed in advance to validate the topic guide, develop proficiency in coordinating and moderating, and to detect any technical challenges. Following each of the first three performed focus groups, the research members met to discuss overall impressions from the sessions and field notes, optimize moderating skills and generate ideas for potential analyses codes. Focus groups continued until thematic saturation was reached. Focus groups were video-recorded, transcribed verbatim, anonymized and safeguarded within LUMC surroundings.

### 2.5 Data analysis

Thematic analysis was executed iteratively to contextualize qualitative data of focus group transcripts. Data was coded deductively with the aid of a designed coding scheme (appendix D p 10) and organized into eight key domains using the Theoretical Domains Framework (TDF): *Knowledge; Skills; Memory, Attention and Decision Process, Professional Role and Identity; Beliefs about Capabilities; Beliefs about Consequences; Emotion; Environmental context and resources*. The TDF-domains provide a granular understanding of the fundamental overarching and interacting constitutes ‘Capability’, ‘Opportunity’ and ‘Motivation’ to generate ‘Behavior’ within the COM-B model.^28^, ^29^, ^30^ This theoretical framework offers practical guidance for the assessment of implementation challenges and facilitators, supporting the design of interventions aimed at behavior change. Subsequent salient themes on implementation strategies were identified inductively (appendix D p11), followed by the design of a decision aid to promote environmentally conscious inhaler decision-making. ATLAS.ti (GmbH, Berlin, Germany, version 23.0) was applied for analysis.

## 3 Results

### 3.1 Participant description

**Fig. 1:**
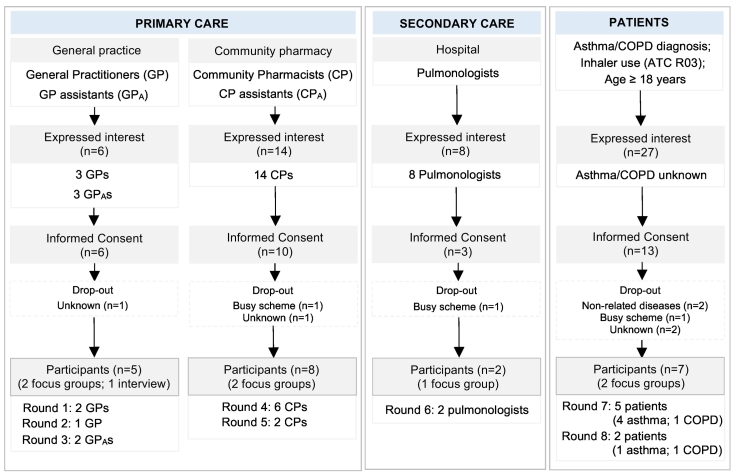
Overview of participating healthcare professionals and patients in focus group discussions. Abbreviations: ATC R03: Anatomical Therapeutic Chemical code for respiratory diseases; COPD: chronic obstructive pulmonary disease; CP_(A)_: community pharmacist (assistant); GP_(A)_: general practitioner (assistant); n: number of (interested) participants.

A series of seven focus groups and one semi-structured interview was conducted with 22 participants until thematic saturation was reached; comprised of 15 practicing HCPs (68·2 %) and seven patients (31·8 %) *(*Fig. 1*)*. Mean duration of focus group discussions was 65 min (range: 42–99 min). Demographic characteristics of HCPs and patients are presented in Table 1, Table 2, respectively.

**Table 1:** Socio-demographic characteristics of healthcare professionals who participated in focus group discussions (*n* = 15).

Healthcare professionals	Participants	Gender	Work experience	Healthcare organization^a^	Located in a healthcare center	Work location
General Practitioners (GP)	GP_1_⁎	Female	26 years	Independent	Yes	Rural
	GP_2_⁎	Female	20 years	Healthcare group	Yes	Urban
	GP_3_⁎	Female	25 years	Healthcare group	Yes	Urban
GP Assistants (GP_A_)	GP_A1_	Female	13 years	Healthcare group	Yes	Urban
	GP_A2_⁎	Female	15 years	Healthcare group	Yes	Urban
Community Pharmacists (CP)^b^	CP_1_⁎	Female	20 years	Franchise	Yes	Rural
	CP_2_	Female	30 years	Independent	Yes	Rural
	CP_3_	Male	3 years	Healthcare group	Yes	Urban
	CP_4_	Female	10 years	Healthcare group	Yes	Urban
	CP_5_	Female	11 years	Healthcare group	Yes	Urban
	CP_6_⁎	Female	21 years	Independent	No	Rural
	CP_7_	Female	4 years	Independent	No	Urban
	CP_8_	Male	5–6 years	Healthcare group	Yes	Urban
Pulmonologists (HP)	HP_1_⁎	Female	7 years	Academic hospital	Yes	Urban
	HP_2_⁎	Male	28 years	Regional hospital	Yes	Urban

Abbreviations: CP: community pharmacist; GP_(A)_:general practitioner (assistant); HCP: healthcare professional; HP: hospital pulmonologist; n: number of HCP's.

a Although multiple healthcare professionals were located in the same organizational setting, participants were located in different practices and pharmacies.

b Managing or supporting pharmacists.

⁎ Specialized in the field of (pharmaceutical) healthcare for asthma and COPD patients and/or sustainable healthcare.

**Table 2:** Socio-demographic characteristics of patients who participated in focus group discussions (*n* = 7).

Lung disease	Participants^a^	Age	Gender	Disease diagnosed	Current inhaler device(s)^b^	Inhaler therapy	Various past inhaler devices
asthma patients (P_A_)	P_A1_	>65 years	female	> 10 years	2 pMDIs + spacer	reliever/controller	yes
	P_A2_	<45 years	male	> 10 years	1 DPI	controller	yes
	P_A3_	45–65 years	male	> 10 years	1 pMDI + spacer; 1 SMI; 1 DPI	reliever/controller	yes
	P_A4_	<45 years	female	6 years	2 pMDIs + spacer	reliever/controller	yes
	P_A5_	<45 years	female	> 10 years	1 DPI, 1 pMDI + spacer	reliever/controller	yes
COPD patients (P_C_)	P_C1_	45–65 years	male	4 years	2 DPIs	reliever/controller	no
	P_C2_	>65 years	female	> 10 years	1 DPI	controller	no

Abbreviations: API: active pharmaceutical ingredient; COPD: Chronic Obstructive Pulmonary Disease; DPI: dry powder inhaler; P_A/C_: asthma/COPD patient; pMDI: pressurized metered dose inhaler; n: number of patients; SMI: soft mist inhaler.

a The disease severity spectrum varied between mild/moderate/severe among the included asthma participants; and was considered mild in the included COPD patients.

b APIs included: salbutamol, salmeterol, formoterol, beclomethasone, ciclesonide, fluticasone, glycopyrronium bromide, umeclidinium bromide and tiotropium bromide.

Out of 15 participating HCPs 13 were employed in primary care (86·7 %): comprised of three GPs (20·0 %), two GP assistants (13·3 %) and eight CPs (53·3 %). Secondary care was represented by two pulmonologists (13·3 %). The majority of HCPs were female (80·0 %), contained >10 years of work experience (93·3 %; range: 4–30 years), practiced in urban areas (73·3 %) and were integrated in an interprofessional collaboration in a healthcare center (86·7 %). Eight HCPs (53·3 %) were involved in sustainable healthcare efforts and/or were specialized in the field of respiratory disease management.

Out of seven participating patients five were diagnosed with asthma (71·4 %) and two with COPD (28·6 %). Patients were predominantly female (57·1 %). Age ranged from <45 years in 42·9 %, between 45 and 65 years in 28·6 %, and > 65 years in 28·6 % of patients. Respiratory diagnosis existed for over more than 10 years in the majority of patients (71·4 %). Currently used inhaler device types of patients included DPIs (71·4 %), pMDIs (57·1 %), and/or SMIs (14·3 %) as reliever and/or controller therapy; of which 28·6 % used multiple inhaler device types (14·3 % a DPI and pMDI; 14·3 % a DPI, pMDI and SMI). Experiences with various inhaler device(s) (types) in the past were reported in 71·4 % of patients.

### 3.2 Perceived implementation challenges and facilitators

Detected challenges and facilitators to implement environmental impact into inhaler treatment decision-making yielded 17 themes within eight predefined TDF-domains related to capability, motivation and opportunity. Emerging themes and illustrative quotes are presented in Table 3 (appendix E contains the complete list of quotes).

**Table 3:** Illustrative quotes of challenges and facilitators to implement environmental impact into inhaler decision-making identified in focus group discussions, integrated under COM-B model constitutes and core TDF-domains^⁎^.

TDF-domains	Themes and illustratieve quotes
Capability	
Knowledge	• Knowledge and awareness on the environmental impact of inhalers
	*“Very exciting, climate-friendly inhalers! But I've no idea what you mean” [P*_*A3*_*]; “I actually didn't think much of it, now that we're addressing it and more is known, environment and climate are of course in the news a lot, that's when you start to think about it more” [P*_*A4*_*]; “I'm very curious about the differences between a DPI and pMDI for the environment” [P*_*A1*_*]; “To what extent is the environmental impact of those pMDIs?” [CP*_*2*_*]; “It's a topic we're working on and which I've heard a few things about from the networking days” [GP*_*3*_*]; “Lately, I've heard about it from different angles…CO*_*2*_*emissions from inhalers, so I've noticed an increased attention. That's good!” [CP*_*8*_*].*
	• Multitude of environmental elements
	*“pMDIs contain at least 200 dosages, if you use it properly it can last a long time while something that contains 60 dosages you'll need more [devices], which has no refill and in turn is all waste. This makes me wonder if plastic production, oil and costs* etc. *are also taken into account before we blame pMDIs for everything?” [GP*_*3*_*]; “I'm surprised when I order rotacaps…first it's in plastic, then aluminum, that's in a box and then another box” [P*_*A3*_*]; “That's also production” [HP*_*2*_*]”; “I think a lot is produced in India, so in terms of distances that is not very convenient for transport” [P*_*A3*_*].*
	• Insufficient overview of critical information
	*“It's important to include the product's footprint from cradle-to-grave, the entire chain. What's happening is ‘cherry picking’, that's a disappointment” [HP*_*2*_*]; “What's wise when you don't have that. I notice then I'm less motivated as I wonder if it's really more environmentally friendly.” [GP*_*A2*_*]; Is there a difference between inhalers with or without a spacer?” [HP*_*1*_*]; “…from start to end, what would it yield if we're to convert everyone? Then HCP's would be more inclined to include sustainability which isn't done currently, other factors weigh more as it isn't clear for us” [CP*_*5*_*].*
Skills	• Inhaler device ability and competence differences
	*“Every form of DPI I've had was easier, they're more compact and you can carry it around” [P*_*A2*_*]; “What a tornado [SMI], what a force to fire off and inhale” [P*_*A5*_*]; “Assembling the Respimat is difficult for which patients visit the pharmacy regularly” [HP*_*2*_*]; “It's not that I don't take my medication, I just don't carry the spacer around” [P*_*A3*_*]; “A lot of people don't use a spacer. I'm surprised they use the one-breath method…while then you might as well use a DPI” [GP*_*3*_*]; “Difficulty is you never know how much dosages are left…I think that's why pMDIs are often thrown away partly full” [GP*_*3*_*].*
	• Inhaler skill development, practice and assessment
	*“Check over time. The first time [patients] say ‘okay I understand’ and then you notice at the next check, it's not so good” [HP*_*1*_*];’I currently no longer experience inhaler issues due to very intensive instructions…that makes a difference” [P*_*A1*_*]; “I think it's [inhaler switch] possible for some, but it requires adequate guidance” [HP*_*2*_*]; “Lung care has also been neglected here, especially after corona. From the pharmacy, but also from GP*_*(A)*_*s less attention has been paid while we know that adherence and use of inhalation medication often goes wrong or certainly requires attention” [CP*_*5*_*].*
Memory, attention and decision process	• Current environmentally friendly inhaler decision influences
	*“I believe we're not very concerned about sustainability in the whim of the day” [GP*_*3*_*]; In principle, [prescribe] DPIs if patients are capable and meet all criteria” [HP*_*2*_*]; I mainly choose pMDIs if people have cognitive problems* e.g. *when home-care plays a role. You can inhale that, even if you can't breathe consciously” [CP*_*6*_*]; People often see someone when they're short of breath and then start a pMDI in emergency or acute situations” [GP*_*3*_*]; “I also consider side-effects, DPIs have more side-effects than pMDIs” [HP*_*2*_*]; “I tend to choose DPIs faster for asthma patients as I have a younger person in front of me. Lately I've been shifting more towards DPIs in COPD patients…nowadays you also have DPIs with low resistance that contain a once daily dose. This is also useful for COPD” [CP*_*6*_*].*
	• Importance of shared treatment decision-making
	*“It depends on the patient whether they want to cooperate but I would certainly suggest it” [CP*_*7*_*]; “I'm open to that and I believe the patient is also open for it in general. It's a hot topic, everyone is talking about it” [CP*_*8*_*]; “You ask people for their preferences and then you offer the possibilities in which you can indicate whether you're environmental conscious or not” [GP*_*3*_*]; “This is how we test it, these are the risks and that it works, that's fine in a safe setting” [P*_*A2*_*]; “I would like to practice with a training device to evaluate if that's something I could use…as there are so many types now” [P*_*A1*_*]; “I would like to be assured patients receive the most suitable inhaler and that this will only be deviated from in consults or with clear reasoning. That we've a choice, do we want that or not?” [P*_*A5*_*].*
	• Prioritization of environmentally friendly inhaler decision-making
	*“I think it's a certainty and right that you get what benefits you, that it isn't a game of Russian roulette” [P*_*A5*_*]; “It's already so complex to find the most suitable inhaler for an individual that sustainability has a lower priority” [CP*_*2*_*]; “Absolutely not [the most important sustainability]! If it [alternative] works and everyone has access, it's [sustainability] a fair third/fourth factor to consider” [P*_*A2*_*]; “I'm positive about taking that step [switch] but it has to remain the same for the patient” [P*_*C1*_*]; “Maybe I would like to think about switching but only under the condition that it works just as well” [P*_*A4*_*].*
**Opportunity**	
Environmental context and resources	• (Potential) environmental stressors
	*“Starting a novolizer is also nice but the DPI does not contain the combination medicine, so you often run into the problem that the ‘series’ isn't complete” [GP*_*3*_*]; “The preference policy doesn't force me to choose a pMDI but it does force me to choose between different DPIs…that's another device with a slightly different resistance” [CP*_*6*_*]; “So in our region people are often switched to pMDIs, then you at least have freedom of choice with a spacer and get a high quality product” [CP*_*1*_*]; “Then you're moving towards a consult, you really have to organize everything, not only the location but also your time and staff” [CP*_*8*_*]; “I really don't have time for it, I'm happy if I can treat patients adequately. Also taking the world issues into account, that's something that wouldn't work” [HP*_*2*_*]; “Low literacy also plays a major role. That causes problems with instructions and which inhaler best to choose. It causes a lot of challenges, especially in the area of communication” [CP*_*3*_*].*
	• (Material) resources
	*“I asked to add more DPI options to the formulary because Foster, as it's a single device, isn't included at the moment…but we've so much Foster in the guidelines with the SMART method, that should also be possible as DPI” [GP*_*3*_*]; “…useful for all prescribers who enter the profession. If they are taught well, they will start sooner with DPIs” [CP*_*3*_*]; “I would strongly argue in favor of expanding and professionalizing educational tools…It could include a paragraph or video on sustainability as ultimately the patient is the end-user, if they realize the footprint, it can help” [HP*_*2*_*]; “There are companies advertising their product is the best, I find that worrying, we don't know that exactly” [HP*_*2*_*].*
**Motivation**	
Professional role and identity	• Environmental responsibility
	*“I believe it would be very good if we're to include sustainability…in many things because there are many aspects regarding medicines and the surrounding industry” [CP*_*1*_*];”I also see its importance and I think people also want to contribute” [CP*_*8*_*];’I must say GP*_*A*_*are very loyal, they attend every training you undertake, GPs are more difficult to motivate, they're engaged in so many topics already or this isn't interesting which makes it difficult as especially GPs start care in acute patients” [GP*_*3*_*]; “In reality, that should be the case but I think right now we're not” [HP*_*1*_*]; “I believe it's very important to implement but it's more important that it has no impact on the people who use it” [P*_*A2*_*].*
	• Potential organizational roles
	*“It starts with the information you receive from your HCP. It hasn't been discussed up to now…so I'm very curious if that will happen the next time, otherwise I will ask about it myself as a result of this conversation” [P*_*C1*_*]; “I don't know how that can be achieved, environmentally friendly, because it's not my profession” [P*_*A5*_*]; “I'm in favor of progressive guidelines but we have to know we're advising correctly” [HP*_*2*_*]; “The DPIs, it's not like we have set a really hard target” [GP*_*3*_*]; “That HCPs have discussed together what is best for the patient and included the sustainability aspect in terms of what's recommended first” [CP*_*8*_*]; “You need to involve all stakeholders, from health insurer to government to patient. You have the strongest and weakest link” [HP*_*2*_*].*
Beliefs about capabilities	• Perceived competence to switch to environmentally friendly alternatives
	*“If patients switch from a pMDI with spacer to a DPI, they might find that more comfortable. So I can see it happening” [CP*_*8*_*]; “There's the In-CheckDIAL© so you can measure whether a patient can do it or not. With the use of this tool, I believe more people use DPIs in our region [CP*_*6*_*];* “*I had the pMDI when I was really short of breath for emergency. Now that I've good treatment, less complaints, I was able to switch to DPI as It's very nice to use” [P*_*A2*_*]; “Then you only look at effect not side-effects, so I believe that proposed number of 70 % is lower but I think 50 % can be converted” [HP*_*2*_*]; “Suppose a patient who has used 10 different devices and is ultimately stable on 1, a pMDI, you're never going to convert that to a DPI. The prior trajectory plays a role in this” [CP*_*8*_*].*
	• Confidence to combat climate change
	*“I believe if you choose to adjust to the CO*_*2*_*impact, you should choose a DPI” [GP*_*3*_*]; “You have to know this makes sense because if you convert everyone now, you will succeed in 5 years' time. Yet, if the carbon footprint of pMDIs is lower than that of DPIs, you've done something wrong” [HP*_*2*_*];’I would say the difference between England and Sweden is very big, that one country has 30 % pMDI and the other country 70 %, there is a whole world of improvement possible in that area” [GP*_*3*_*].*
Beliefs about consequences	• Anticipated regret to prioritize environmentally friendly alternatives
	*“We're not talking about plastic or paper straws…they're people with sensitive lungs” [P*_*A2*_*]; “I almost lost my voice which had a lot to do with the ingredients of inhalers, that's why I switched 4–5 times until I finally had something with the same good effect and suffer less from” [P*_*A3*_*]; “Pharmaceutical companies all produce their own device with various underlying ideas, making it difficult to exchange 1:1” [P*_*A4*_*]; “Try it out with the option that if you don't like it, you can switch back to the previous device, no matter how environmental-unfriendly” [P*_*A1*_*]; “The fact that in two years' time propellants will be less harmful. In 2040 or 2050 I believe the propellants should have a 95 % lower CO*_*2*_*impact” [HP*_*2*_*].*
	• Poor outcome expectancies of non-medical inhaler switching
	*“That may not benefit therapy adherence” [CP*_*1*_*]; “Environment is important but it also has to work. If someone is taken to hospital by ambulance and is hospitalized for a week, then I think you've emitted more CO*_*2*_*than if you just inhaled normally” [P*_*A5*_*]; “I think we should discuss this in a transcendent manner, otherwise the patient will think environment or money is the driver. There's so much distrust in healthcare, we've to be careful” [HP*_*2*_*]; “I believe it's great the environment is taken into account and necessary but I'm afraid we'll overshoot the target, namely care for our lungs. I hope a balance remains in what's good for the environment and care for lung patients remains top priority” [P*_*A5*_*].*
Emotions	• Reaction pattern on environmentally friendly inhaler decision-making
	*“When I hear what you said about the differences…it's clear what direction to go” [P*_*C1*_*]; “I'm very curious what alternatives are out there instead of pMDIs…whether you'll receive DPIs or if there are other options” [P*_*A1*_*]; “I've never heard of it going wrong, maybe this is my imagination but sometimes I find it [switch] a scary idea” [P*_*A2*_*]; “It may also be a fear that you think the patient does not have enough strength and it [DPI] will not reach its target site” [CP*_*6*_*];’I absolutely don't want them [health insurers] to determine that I must have a certain inhaler if it doesn't work for me” [P*_*A4*_*]; “I find it difficult because I've also used those DPIs and that didn't go well so I'm like ‘not with my body’ but then I hear these numbers, then perhaps I need to start looking at DPIs at some point” [P*_*A1*_*].*

The complete list of quotes is available in appendix E (p 11–29).

Abbreviations: COM-B model: Capability, Opportunity, Motivation, Behavior; COPD: Chronic Obstructive Pulmonary Disease; CP: community pharmacist; DPI: dry powder inhaler; GP_(A)_: general practitioner (assistant); HCP: healthcare professional; HP: hospital pulmonologist; P_A/C_: asthma/COPD patient; pMDI: pressurized metered dose inhaler; SMART: Single Maintenance And Reliever Therapy; SMI: soft mist inhaler; TDF: Theoretical Domains Framework.

#### 3.2.1 Capability

##### 3.2.1.1 Knowledge

Although an increasing affinity with sustainable healthcare was observed in all participants, awareness of the substantial impact of inhalers was lagging behind, especially in participants without a personal interest and/or involvement in related activities. Yet, the uprise of educational efforts and campaigns was perceived a fundamental facet to generate more attention and awareness on the topic. The lack of a complete and transparent overview of critical information regarding the overall carbon footprint life-cycle assessment from ‘cradle-to-grave’ and gains on an individual level were often reported as prominent implementation barriers, holding back its translation into clinical practice and explanation to patients accordingly. Notably, participants expressed their concern on packaging waste as well as the scarce number of re-usable inhalers.

##### 3.2.1.2 Skills

DPIs were widely preferred among participants since they are easy to use, more practical within an active lifestyle due to its compact design and often contain built-in dose-counters preventing a rapid inhaler turnover or the use of empty inhalers. Though, a minimal inspiratory effort was required, which was viewed by some as less appropriate in emergency situations. SMIs were also discussed as environmentally friendly option specifically due to its re-usability, but its complexity in use often withheld its widespread applicability. In contrast, pMDIs were considered universal due to the utilization of a spacer or VHC, minimizing dependency on inspiratory flow and coordination. However, this attachment often impacted portability and required regular maintenance, which often resulted in non-adherence. A GP stated if patients were able to handle pMDIs without spacer, they were capable of adapting to DPIs.

Regardless of device type, initiation of a new inhaler was regarded a careful process by participants requiring regular demonstrations and reviews of inhaler technique, education and self-management support to ensure correct use and promote adherence. Though, both patients and HCPs acknowledged a decrease in on-demand or opportunistic respiratory care, especially since the COVID-19 pandemic.

##### 3.2.1.3 Memory, attention and decision process

Minimal efforts were undertaken by HCPs to factor environmental impact into inhaler decision-making, while patients hardly raised any inquiry on the topic. DPIs were however considered first choice device by HCPs, especially for patients within the age group of 10–65 years. In contrast, pMDIs were particularly intended for vulnerable patients (e.g. children <10 years, severe disease status and nursing homes). Nonetheless, some acknowledged an underlying prescribing habit due to a presumed lack of adequate inspiratory flow and cognitive status in elderly patients, leading to more DPIs in asthma and pMDIs in COPD. These concerns were however dismissed throughout discussions and likely only to apply to a small minority of patients, regardless of age. Though, pMDI prescriptions were also sensed to be a result of the continuation of previous inhaler requirements in acute situations. The advancing DPI landscape and diversity in flow-rate characteristics was considered a facilitator by some, while the different designs and product quality constrained others. Furthermore, persistent local adverse effects induced by the dry powder causing throat irritation also precluded the use of DPIs in some patients.

Meanwhile, the majority of HCPs and patients indicated their willingness to opt for environmentally friendly inhaler alternatives if clinically appropriate and performed as an outcome of SDM. Notably, an objective discussion on the topic, including a risk-benefits assessment and the exhibition of a selection of options through placebo devices, was indicated to empower patients in the process. Efficacy, safety and ease of use however remained priority over environmental impact when choosing inhaler devices. Particularly, patients accentuated the preservation of inhaler choice freedom.

#### 3.2.2 Opportunity

##### 3.2.2.1 Environmental context and resources

Limited availability of inhaler medication in various device types was an identified barrier, also hindering uniformity in device type when prescribing multiple inhalers. Moreover, inhaler devices are regularly alternated due to variations in product reimbursement by health insurers, inhaler shortages, parallel imports and patient alternations of health insurance companies. Consequently, some HCPs prioritized pMDIs due to a greater interchangeability of devices within this group. Concerns were also raised regarding time investment and appropriate training, specifically when switching between inhaler device types. Clearly, it was considered more appropriate to factor the environmental impact when selecting an inhaler at treatment initiation or re-evaluation (e.g. when detecting incorrect inhaler use and/or exacerbations). Language barriers and limited health literacy skills were reported as communicational challenges.

HCPs also expressed the need for practical implementation guidelines and tools, to broaden DPI options in policy documents, and to expand educational material to inform both patients and HCPs on environmentally friendly actions. Conversely, use of promotional product material from pharmaceutical industries was considered unreliable and therefore discouraged.

#### 3.2.3 Motivation

##### 3.2.3.1 Professional role and identity

Most participants felt a moral responsibility for the integration of environmental impact into inhaler treatment decision-making. A leading role was especially indicated for HCPs who served as the primary source of medical expertise, while patients reported a limited decision-making capacity in this field. Though, some GPs revealed implementation commitments were often made based on their personal interests in specific topics to manage their workload. Others reported the need for policy coherence including a situation analysis and a clear vision on the defined targets, strategies and arrangements. A multidisciplinary approach was also highlighted to coordinate sustainable actions efficiently, engaging all key stakeholder groups (e.g. health-insurers, pharmaceutical companies and government).

##### 3.2.3.2 Beliefs about capabilities

Overall, HCPs believed that the vast majority of asthma and COPD patients could be shifted from pMDIs to DPIs, particularly due to the reported advantages in design and ease of use. Additionally, utilization of the InCheck-DIAL® (Alliance Tech Medical, Granbury, TX) was highly recommended as tool to assess patients' ability to use a DPI and coach inhalation technique.^31^ In relation, some HCPs have already successfully initiated or switched a great number of patients to DPIs accordingly, suggesting its feasibility in clinical practice. In turn, some patients reported their willingness to try-out DPIs only if guaranteed to switch back to the previous inhaler if the change would not suit them. Yet, what level of implementation could be achieved among (un)stable patients was debated on and may be influenced by the patients' prior inhaler trajectory. One GP considered the lower pMDI prescription rates in Sweden as potential to advance in this area in the Netherlands.

Importantly, focus group discussions highlighted the participants' belief to reduce inhaler-related GHG emissions by combining *indirect* approaches optimizing effective respiratory management with *direct* approaches focusing on the prescription of environmentally friendly alternatives.

##### 3.2.3.3 Beliefs about consequences

Some participants suggested that inhaler switching without medical justification could lead to reduced adherence, poor disease outcomes and subsequent increased healthcare utilization. Hence, ill-advised switches were considered to indirectly contribute to carbon footprint due to increased hospitalizations and (medication) waste. Though, the extent of occurrence remained unclear. Moreover, pharmaceutical excipients of inhalers were acknowledged to affect patients' experience of side-effects, which could pose as a significant challenge for inhaler switching. Consequently, several HCPs noted a potential loss of patient trust and confidence in the healthcare system, which was confirmed by patients whom feared that the environmental impact could take precedence over their own health. Whereas evolving innovations regarding a transition to lower-GWP propellants coincided with efforts of inhaler switching.

##### 3.2.3.4 Emotion

Overall, participants were shocked by the environmental impact of inhaler devices which instigated their curiosity to explore alternative environmentally friendly options. Some patients however perceived inhaler switching as scary due to potential health consequences. Likewise, a cognitive dissonance was sensed consisting of a positive attitude towards environmentally friendly actions and feeling discomfort regarding behavior change. Conversely, some HCPs observed a restraint to initiate DPIs in general out of fear for potential insufficient inspiratory flow in patients. Participants explicitly raised their concerns regarding the preferential policy of health-insurers as potential driving force for sustainable choices, posing a threat to inhaler choice freedom.

### 3.3 Perceived implementation strategies

**Fig. 2:**
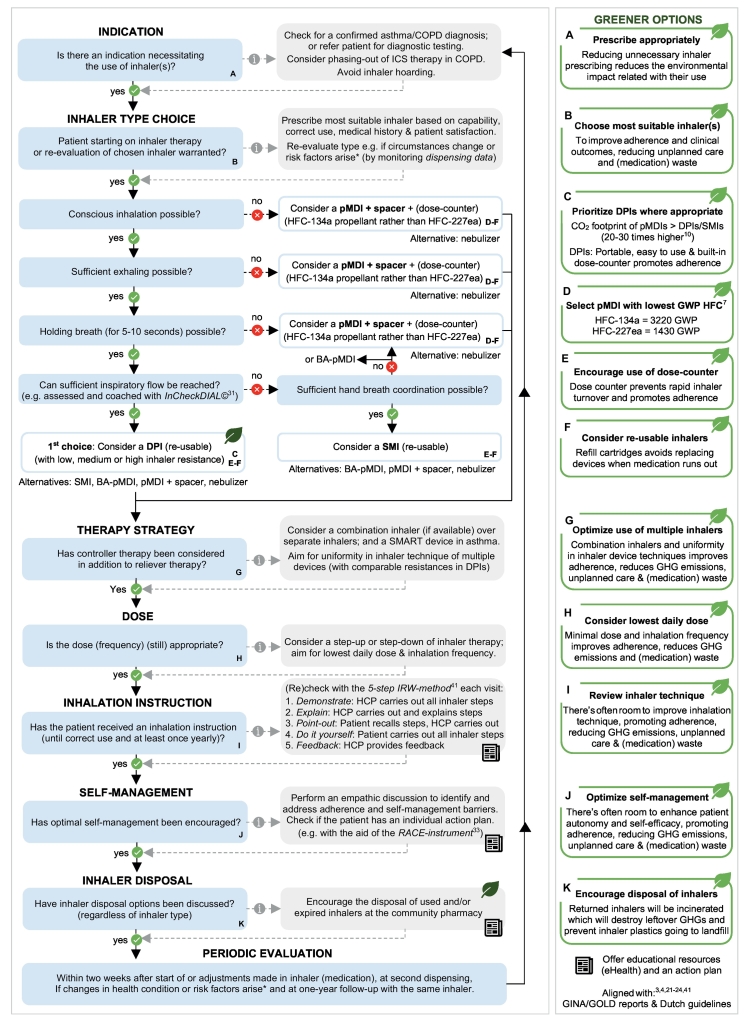
Proposed flowchart for (environmentally friendly) inhaler treatment decision-making in asthma and COPD. ^⁎^Uncontrolled disease or patients at risk for adverse outcomes (e.g. overuse/underuse of inhaler therapy, exacerbations). Abbreviations: B: breath actuated; COPD: chronic obstructive pulmonary disease; DPI: dry powder inhaler; HCP: healthcare professional; HFC: hydrofluorocarbon; IRW: inhaler research workgroup; GHG: greenhouse gas; GINA: Global Initiative for Asthma; GOLD: Global Initiative for Chronic Obstructive Lung Disease; GWP: global warming potential; pMDI: pressurized metered dose inhaler; RACE: respiratory adherence care enhancer; SMART: Single Maintenance And Reliever Therapy; SMI: soft mist inhaler.

Five main action areas for implementation strategies were uncovered, derived from information proposed by HCPs and patients, to be reflected in health policy decisions including: 1) communication, education and awareness, 2) appropriate inhaler prescribing, 3) promotion of smarter inhaler choices, 4) optimization of quality of care and 5) appropriate inhaler disposal. Emerging themes and illustrative quotes are presented in Table 4 (appendix E contains a complete list of quotes). Accordingly, a decision aid was designed by the research team to assist environmentally friendly inhaler treatment decision-making in asthma and COPD in clinical practice (Table 5*;* Fig. 2).

• *Communication, education and awareness*

**Table 4:** Illustrative quotes of potential strategies to implement environmental impact into inhaler treatment decision-making identified in focus groups discussions, integrated under five action areas^⁎^.

**Action areas**	**Themes and illustrative quotes**
Communication, education and awareness	• Relationship-building and educational efforts on topic
	*“To discuss the sustainability aspect, present numbers, so instead of pMDIs as standard prescription, that they consciously think about starting DPIs if the patient can handle it” [CP*_*7*_*]; “Of course we'll consider what you've presented here in the PTAM and better inform doctors about the options available and not to choose for pMDIs so easily” [CP*_*3*_*]; “You first have to generate some support” [P*_*A4*_*]; “We could position that [link to educational patient material] even stronger, also when prescribing medication. I always have a reference on my prescription” [HP*_*2*_*].*
	• A collective environmentally friendly action plan
	*“The first step is to make agreements with GP*_*(A)*_*…” [CP*_*7*_*]; “So we're going to assess what you could do in your own practice, also focus on medication use, what percentage of pMDIs do we actually use and can we do something about that? We make that discussable with each other, then we make a plan with everyone, what they would like to get started with” [GP*_*2*_*]; “This has to be performed regional or even smaller, making those agreements with each other” [CP*_*8*_*].*
	• Cyclic monitoring of environmentally friendly actions
	*“This contains some investment to keep it going and also to go back to your doctors every now and then, ‘hey, listen, we agreed this and that’. That also depends on your PTAM structure” [CP*_*6*_*]; “If we want to find out the status of things, indeed overuse of salbutamol, then I ask the pharmacy for a printout of patients who use too much” [GP*_*2*_*].*
Appropriate inhaler prescribing	• Reduce unnecessary inhaler use
	*“It's also about the theme doing or not doing, that's not only sensible care but also sustainable care. So we make that a topic of discussion with each other [GP*_*1*_*]; “I believe in optimal use and phasing-out of medicine when necessary so people do not stay on inhalers for an unnecessarily long time” [CP*_*7*_*]; “Those ICS, which can be reduced at a certain point in COPD, that should* e.g. *be closely monitored” [CP*_*7*_*]; “In COPD it hasn't been proven that ICS helps. The assignment was to stop Seretide in COPD patients and only provide a bronchodilator. We did that in the majority of patients. A few patients did not want to switch, so I left it. While some who switched got more exacerbations…for this patient it worked apparently. So I find that difficult” [GP*_*A1*_*].*
Promote smarter inhaler choices	• Prioritize DPIs as environmentally friendly inhaler *(if appropriate)*
	*“Especially not to prescribe pMDIs” [GP*_*1*_*]; “When it comes to sustainability, I think that if you prescribe something, then consider what kind of medication to prescribe” [GP*_*2*_*]; “We've been busy in our care group to reduce pMDIs. We've chosen, I believe, age >* *10 years and maybe <* *70 or 75 years to start converting those people and consciously explained to patients it's because of the environment” [GP*_*2*_*]; “For starters it's easier as people don't know the difference yet, while if you already have one you often have people who prefer the previous device” [GP*_*A2*_*].*
	• Consider a sustainable device design and dosing treatment regimen *(if appropriate)*
	*“Prescribe small quantities” [GP*_*1*_*]; “Dosing frequency, as the more you use, the more…with the lowest possible dosage as why use more medication if it isn't necessary?” [HP*_*2*_*]; “Dosing once or* max *twice daily, that's also something I think is important, also to ensure people use it” [CP*_*6*_*]; “If patients use several inhalers, a combination inhaler is often a solution, increasing patient compliance” [CP*_*8*_*]; “If they use multiple inhalers, that they get the same type not everything mixed together” [GP*_*A1*_*]; “That the discus itself can last longer and does not have to be replaced often” [P*_*C2*_*]; “We might also have to think about re-using devices but that also has to remain convenient for the patient, that they get it in easily” [CP*_*4*_*]; “Or another type of propellant?” [P*_*C1*_*].*
Optimization of quality of care	• Support individualized self-management of inhaler use in patients
	*“Whether patients use it faithfully or not as that's where the effectiveness lies [HP*_*2*_*]; “Always ask why they use something or not and how often. A lot goes wrong in that area, it's important to get a good view of why it does not work or people don't do that” [GP*_*A2*_*]; “Underuse, which you can detect well by what has actually been collected in the pharmacy in the past period and sometimes it's also an incorrect inhalation technique. Aspects that all have to be checked” [GP*_*3*_*]; “Number of exacerbations someone has and use of SABA. If that's high and they also had a prednisone course, those are people where at least the inhalation technique should be re-evaluated. Perhaps you'll come to the conclusion to switch to a different device” [CP*_*1*_*].*
	• Regular assessment and ongoing education on correct inhaler technique
	*“I believe we can gain a lot with inhalation instructions and adequate use” [HP*_*2*_*]; “Literally placed the inhaler in the middle of the room, thought I'll breathe that in or something. I had really given an instruction but it didn't come through” [GP*_*A1*_*]; “Of course I've seen them all [inhaler products] at some point but the last IMIS I did was probably three years ago. In the meantime a lot of new devices have been released” [GP*_*2*_*]; “Pulmonary nurses talk a lot about the one-breath method, especially with the spacer…but you actually shouldn't do that with pMDIs” [CP*_*1*_*].*
Appropriate inhaler disposal	• Return inhalers to the community pharmacy
	*“We must support patients to not throw away their DPIs plastic but to dispose of it” [HP*_*2*_*]; “That they [inhalers] can be handed in at the pharmacy as now we throw it in one pile of chemical waste” [CP*_*7*_*]; “I believe that's the pharmacist’ job, that's where they receive medication and where the boxes stand to hand it in” [GP*_*1*_*].*
	• Set-up of inhaler recycling scheme
	*“There's a lot to be gained in terms of material, that's industrial design and I've no idea how to replace plastic…but suppose you're to make a mountain of all inhalers used in a year, how much plastic that would be” [P*_*A3*_*]; “I believe the manufacturer can play a role in terms of making sure it's made from recycled plastic…maybe special recycling bins can separate inhalers” [CP*_*7*_*].*

*The complete list of quotes is available in appendix E (p 30–32).

Abbreviations: COPD: Chronic Obstructive Pulmonary Disease; CP: community pharmacist; DPI: dry powder inhaler; GP_(A)_: general practitioner (assistant); HCP: healthcare professional; HP: hospital pulmonologist; ICS: inhalation corticosteroids; IMIS: Inhalation Medication Instruction School; P_A/C_: asthma/COPD patient; pMDI: pressurized metered dose inhaler; PTAM: pharmacotherapeutic audit meeting; SABA: short-acting β_2_-agonist; SMI: soft mist inhaler.

**Table 5:** Proposed decision aid to assist in environmentally friendly inhaler decision making in asthma/COPD patients at treatment initiation or re-evaluation, if clinically appropriate and performed as outcome of shared-decision making*.

Guide	Practical environmentally friendly considerations for inhaler decision making in patients^3^^,^^4^^,^^19^, ^20^, ^21^, ^22^
**Step 1**	**(*Re*-)evaluate the indication for inhaler prescribing.**
	*Tackling inappropriate inhaler prescribing reduces unnecessary inhaler use and its environmental burden.* e.g. *avoid inhaler hoarding and consider phasing-out of inhalation corticosteroids in COPD.*
**Step 2**	**Consider a DPI as first choice device if the patient is capable** (in particular in patients between 10 and 65 years)**.** (e.g. check the required inhalation criteria and coach the required inspiratory flow with the In-Check DIAL©)**.**
	*DPIs are widely preferred by asthma and COPD patients since they do not contain GHGs, are easy to use, practical due to its compact design and often contain built-in dose-counters. DPIs require the following inhalation criteria: conscious inhalation, sufficient exhaling, holding breath for 5–10* *s and a sufficient inspiratory flow. There is a great diversity in DPIs available with various inspiratory flow-rates (mild-moderate-high resistance).*
**Step 3**	**If pMDIs are warranted, aim for a pMDI with the lowest GWP propellant (HFC-134a).**
	*pMDIs with a spacer or valved holding chamber are particularly intended for vulnerable patients (*e.g. *children < 10 years, severe disease status and nursing homes). pMDIs containing HFC-134a as propellant are preferred due to its lower GWP (∼1430 times that of CO*_*2*_*) over pMDIs containing HFC-227ea (∼3220 times that of CO*_*2*_*).*^7^
**Step 4**	**Choose an inhaler device with a built-in dose-counter or add an attachment** (e.g. CountAir®)**.**
	*A dose-counter allows patients to keep track of the number of dosages in the inhaler, counting down after each inhalation. This prevents a rapid inhaler turnover and promotes inhaler adherence.*
**Step 5**	**Consider a re-usable inhaler device where the cartridge is replaced.**
	*Refill cartridges avoids replacing inhaler devices when medication runs out, decreasing environmental burden.*
**Step 6**	**If multiple inhalers are warranted, consider a combination inhaler or aim for uniformity in device types.**
	*Utilizing various inhaler devices and/or types can be confusing to patients due to different inhaler techniques and decrease efficacy* e.g. *consider a SMART device with ICS-formoterol and avoid SABA monotherapy in asthma.*
**Step 7**	**Aim for the lowest daily treatment dose and frequency.**
	*Minimal dose and inhalation frequency improves adherence, reduces GHG emissions and/or (medication) waste.*
**Step 8**	**Review the patients' inhaler technique at least once yearly.**
	*Inhaler technique must be addressed to identify areas of improvement, promoting adherence and drug deposition in the lungs. This reduces the need to escalate treatment, reduce potential hoarding and (medication) waste.*
**Step 9**	**Review the patients' self-management behavior** (e.g. overuse and/or underuse of inhalers)**.**
	*Discussing behavioral barriers and designing an action plan aligned with personal goals can improve patient autonomy, enhancing self-efficacy, patient satisfaction and adherence.*
**Step 10**	**Encourage patients to return used, unwanted or expired inhalers to the community pharmacy.**
	*Landfill disposal of inhalers is harmful to the environment as leftover gases are released into the atmosphere and plastics cannot be recycled using domestic recycling schemes. Returned inhaler devices will be incinerated which will destroy leftover GHGs and prevent inhaler plastics going to landfill.*

Considerations are based on focus group discussions with healthcare professionals (n = 15) and asthma/COPD patients (n = 7). Abbreviations: COPD: Chronic Obstructive Pulmonary Disease; DPI: dry powder inhaler; GWP: global warming potential; HFC: hydrofluorocarbon; ICS: inhalation corticosteroids; IMIS: Inhalation Medication Instruction School; pMDI: pressurized metered dose inhaler; SABA: short-acting β_2_-agonists; SMART: Single Maintenance And Reliever Therapy.

Participants marked relationship-building and educational efforts among HCPs and patients as vital components to achieve credibility for environmentally friendly agreements and actions (e.g. through pharmacotherapeutic audit meetings (PTAM) between GPs and CPs, (accredited) training programs and in consults). Likewise, continuous monitoring and evaluation of prescription behavior was deemed essential by HCPs to provide insights into the current status and impact of interventions (e.g. through pharmacy dispensing data). Integration of educational material (e.g. through eHealth) was reported pivotal to involve and support patients in the process of environmentally sustainable healthcare.

• *Appropriate inhaler prescribing*

Tackling inappropriate inhaler prescribing was reported by HCPs to reduce unnecessary inhaler use and its corresponding environmental burden. Correspondingly, some reported the option of (cautiously) phasing-out inhalation corticosteroids (ICS) as maintenance therapy in COPD patients for whom a clear indication is lacking.

• *Promotion of smarter inhaler choices*

Prioritization of DPIs was encouraged as environmentally friendly alternative by most HCPs and patients, if clinically appropriate. Whereas if pMDIs remained indispensable, one patient reported a potential transfer to a lower-GWP propellant inhaler. Additionally, various device designs (e.g. presence of a dose-counter, re-usability, combination inhaler and/or uniformity of multiple inhaler devices) and treatment regimen options (e.g. lowest-dose (frequency) and single maintenance and reliever therapy (SMART)) were accentuated to further refine and control (sustainable) healthcare.

• *Optimization of quality of care*

Individualized self-management support was emphasized by HCPs to identify and address underlying patient perceptions related to overuse of reliever therapy and/or underuse of controller therapy. Moreover, repeated education and training for HCPs and patients was warranted to increase familiarity with inhaler device products, correct techniques for teaching about and/or using these devices.

• *Appropriate inhaler disposal*

HCPs encouraged the return of used, unwanted or expired inhalers to community pharmacies for safe disposal as opposed to landfill. In turn, the launch of a recycling inhaler scheme was suggested by both HCPs and patients to recover inhaler residual. This also highlighted the importance of informing patients to support environmentally conscious inhaler disposal.

## 4 Discussion

This qualitative study conveyed new insights into perceived challenges, facilitators and strategies to factor environmental impact into inhaler decision-making with personalized care in asthma and COPD. Overall, HCPs and patients demonstrated their responsibility and willingness to opt for environmentally friendly inhaler actions including a shift from pMDIs towards DPIs, if clinically appropriate and performed as outcome of SDM. Notably, unawareness of the environmental impact of inhalers and a lack of policy coherence on subsequent opportunities to advance in this area were prominent barriers hampering its implementation. Accordingly, an inhaler treatment decision aid was realized to enhance salience, credibility and legitimacy to foster environmentally conscious behavior change in the near future.

To our knowledge, this study is the first to have explored the perspectives of HCPs across various medical specialties and patients on factoring environmental impact into inhaler treatment decision-making in the Netherlands, utilizing a theoretical framework and hybrid thematic analyses approach. With the applied TDF and COM-B model as theoretical basis, the perceived implementation challenges and facilitators covered eight key domains related to capability, opportunity and motivation. Following, five main action areas were highlighted containing a range of potential opportunities to improve quality of care while simultaneously reduce GHG emissions. This supports the notion that implementation of environmentally friendly behavior change comprises a complex multifactorial and multicomponent intervention approach. To date, a number of empirical studies have demonstrated promising results for the utilization of a theoretical basis in the identification and targeting of behavioral challenges related to adherence and self-management.^32^, ^33^, ^34^ Likewise, the integration of evidence- and theory-based interventions is endeavored by the Medical Research Council for the development and evaluation of complex interventions.^35^ Importantly, implementation of environmentally friendly actions was perceived counterproductive without patients' capability and acceptability, adversely affecting established treatment routines, adherence, number of inhalation technique errors, disease outcomes and carbon footprint.^19^

The present study revealed that the majority of HCPs and patients prioritized the use of DPIs due to its advantages in design and ease of use. Taking the lower environmental impact into account, DPIs were considered an even more appealing option. Moreover, most patients between 10 and 65 years were considered capable of using DPIs, making a shift from pMDIs towards DPIs likely, especially at treatment initiation or re-evaluation. In accordance with literature, especially the elderly and children or patients with a severe disease state may experience reduced effectiveness when using DPIs due to insufficient inspiratory force and unfamiliarity with the device.^19^ Collaboration between researchers and all relevant stakeholders was continuously articulated across all participants to motivate the uptake of evidence-based policy within this relatively new field in clinical practice. A particular evidence gap included the lack of comprehensive life-cycle footprint assessments for inhalers currently on the market. Future research should therefore endeavor a homogeneous methodology including more robust data sources, social and economic factors and standardized reporting and communication to ensure comparability and consistency in the impact assessment.^36^ This will allow for more informed sustainable inhaler decision- and policy-making, crucial for the transitioning towards a more circular economy, minimizing carbon footprint. Furthermore, an interdisciplinary approach was highlighted to engage stakeholders of all healthcare-levels and groups to maximize successful implementation. Performance management was cited an iterative process of careful planning, delivering, monitoring and evaluating.

Meanwhile, the pharmaceutical industry has made significant progress in the development of lower-GWP propellant use in pMDIs. As a result, a transition to HFC-152a (GWP 138) could potentially reduce the environmental impact of currently available pMDIs with >90 %, placing it within the range of DPIs and SMIs.^37^ This is especially meaningful for (vulnerable) patients whom are dependent on pMDIs. Though, several regulatory hurdles still need to be overcome including long-term safety and efficacy assessments before novel pMDI products based on HFA-152a will be approved and reach the market. Nonetheless, participants advocated a spectrum of promising environmentally friendly inhaler actions to help mitigate climate change related to disease management changes on both levels of disease control as well as the overall GWP of inhaler usage.

In relation, previous studies have demonstrated a misdiagnosis prevalence of up to 60 % in asthma and COPD worldwide.^38^^,^^39^ To minimize overdiagnosis and the prescribing of non-indicated inhaler therapies accordingly, patients with suspected and unconfirmed asthma or COPD should therefore be further investigated to elucidate their underlying true diagnosis.^40^ Moreover, to achieve optimal benefit from inhaler therapy, patients should follow the prescribed treatment regimen persistently and use their inhaler correctly to ensure adequate medication delivery to the lungs. Yet, suboptimal self-management of inhalation therapy has been observed in >50 % of patients, associated with an increased risk of exacerbations, hospitalization, and mortality.^41^^,^^42^ Meanwhile, poor treatment adherence to controller therapy has demonstrated to increase the overall carbon footprint, due to (over)use of inhaled reliever therapy which is typically delivered through high-GWP pMDIs.^43^ Notably, numerous strategies have been identified to enhance medication adherence including the use of dose-counters, combination inhalers and/or uniformity in inhaler devices, lowest daily treatment dose and frequency, and educational efforts. Though further environmental benefits may also come from reusable and recyclable inhalers, and appropriate inhaler disposal.

This study has several limitations. First, the reported findings may be prone to selection bias inherent to a small convenience sample of voluntary participants who may be more active and/or interested in the field of sustainable or respiratory healthcare. However, the involved participants could be more eager to improve care for respiratory patients and therefore be more inclined to an objective approach, acknowledging potential pitfalls. In addition, purposive sampling was performed to ensure heterogeneity in participants, characteristics and inhaler device use. Second, although the inclusion of four different professions as well as both asthma and COPD patients provided a broad overview of perspectives, the generated data may not give a full representative insight as this design could not test data saturation within each healthcare profession and patient group.^44^ Third, the focus groups and interview were performed virtually which could have influenced participant interaction and the generation of in-depth data in comparison to face-to-face sessions. Though, some studies have reported contrary findings for which virtual sessions are favored in certain contexts, in particular for sensitive topics (e.g. planetary health). Moreover, virtual sessions can include geographically diverse participants which can be more difficult to attain in a face-to-face environment.^45^ Fourth, the included HCPs predominantly consist of pharmacists which may have influenced the data generated. Yet, pharmacists have been recognized as an essential component of the multidisciplinary team due to their clinical-pharmaceutical expertise whom also promote correct use of inhalers in patients.^23^^,^^24^^,^^46^ Fifth, the healthcare setting and organization of care for asthma and COPD patients in the Netherlands may not be representative for other countries due to differences in infrastructure, responsibilities and roles, education and reimbursement policies. These countries may therefore experience other implementation challenges, facilitators and strategies specific for their healthcare setting. Though, a definite advantage of utilizing a theoretical framework is that findings can be compared throughout different settings. Finally, a multi-stakeholder approach was encouraged for which perspectives of health insurers, the government and pharmaceutical companies could be useful to further tailor a successful implementation strategy in clinical practice. This involves careful navigation of power differentials and the establishment of clear governance structures, fostering trust and transparency, and adapting co-design processes to the specific context. By acknowledging and engaging with the defined potential drivers, the transition process towards sustainable respiratory healthcare will be substantially increased.^47^

In conclusion, this study provided a broad overview of implementation challenges, facilitators and strategies to factor environmental impact into inhaler treatment decision-making in asthma and COPD. These findings guide the delivery of a range of opportunities to improve quality of care while simultaneously reduce carbon footprint. This will require a multifactorial and interdisciplinary approach with HCPs playing a central role in engaging and educating patients to determine the viability of environmentally friendly alternatives, promote correct inhaler use and appropriate disposal. Future research has to reveal the feasibility and effectiveness of this environmentally conscious behavior in clinical practice.

## CRediT authorship contribution statement

**Claire D. Visser:** Writing – original draft, Methodology, Investigation, Conceptualization. **Alan Sulaiman:** Writing – review & editing, Methodology, Investigation. **Narrin Bakr:** Writing – review & editing, Methodology, Investigation. **Henk-Jan Guchelaar:** Writing – review & editing, Methodology, Conceptualization. **Martina Teichert:** Writing – review & editing, Methodology, Conceptualization.

## Funding statement

This work was supported by 10.13039/100004325AstraZeneca (grant number 2605096485) and the Royal Dutch Pharmacists Association (KNMP) (grant number PR20_002) with unconditional research grants. The funders were not involved in the design, content and conduct of the study, analyses and interpretation of the data including the writing of the report.

## Declaration of competing interest

MT received unconditional research grants from AstraZeneca and the KNMP for the advancement of pharmacy. The PhD project of CV is funded with these grants. AS and NB contributed equally to this manuscript as master pharmacy students at the Leiden University. The remaining author HJG declared no competing interests for this work.

## Data Availability

The raw data supporting the conclusions of this article will be made available by the authors upon reasonable request, without undue reservation.

## Appendix A Supplementary data

Supplementary material Image 1
