# Supplementary material for: Environmentally friendly inhaler decision-making with personalized care in asthma and chronic obstructive pulmonary disease: a qualitative study
Source: Explor Res Clin Soc Pharm. 2025 Aug 29;20:100651. doi: 10.1016/j.rcsop.2025.100651 (PMC12455118; doi:10.1016/j.rcsop.2025.100651)
Supplement: Supplementary file 1 — Supplementary material [file mmc1.docx]

**Supplementary material**

Supplement to: ‘’Environmentally friendly inhaler decision-making with personalized care in asthma and chronic obstructive pulmonary disease (COPD): a qualitative study’’.

**Contents**

[**Appendix A. Study procedures flowchart** 2](#_Toc183607152)

[**Appendix B. Topic guides semi-structured focus groups** 4](#_Toc183607153)

[**Appendix C. Search strategy systematic literature review** 8](#_Toc183607154)

[**Appendix D. Coding scheme qualitative data** 10](#_Toc183607155)

[**Appendix E. Perceptions of participants from focus group discussions** 12](#_Toc183607156)

# **Appendix A. Study procedures flowchart**


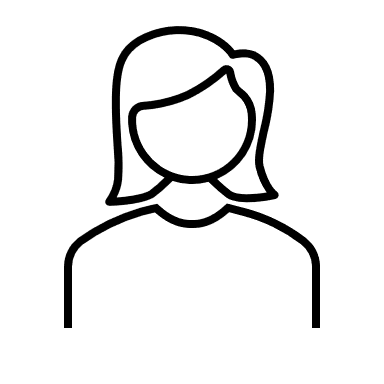

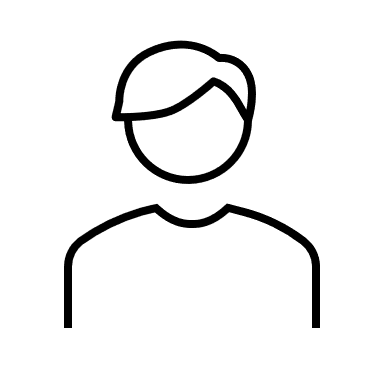

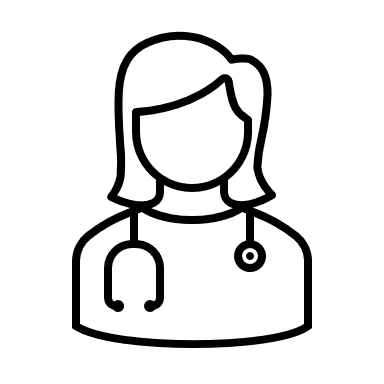


**A**

**Recruitment of primary and secondary healthcare professionals**


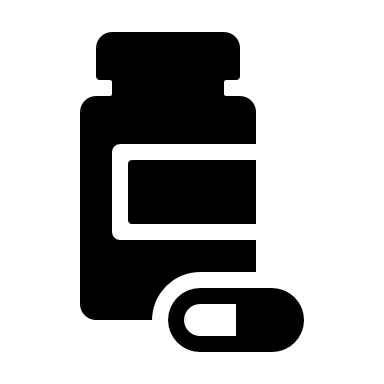

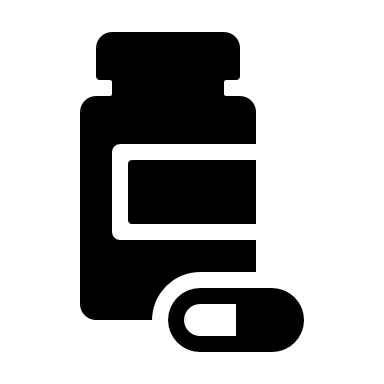

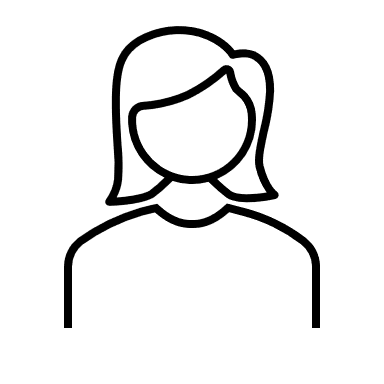

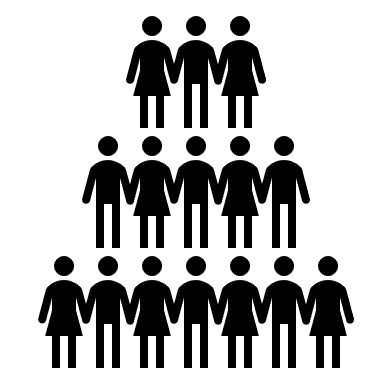

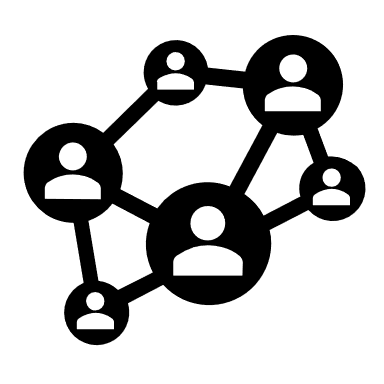

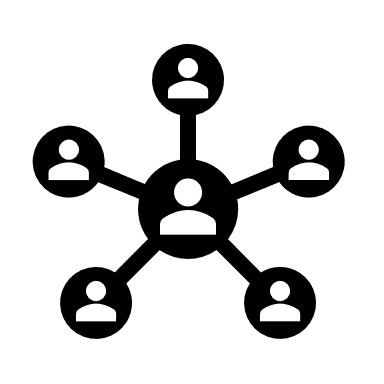


CP_A_s

CPs

GP_A_s

GPs

Primary care


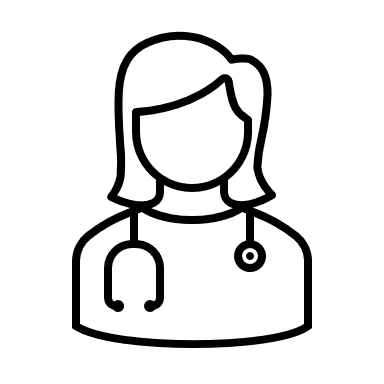

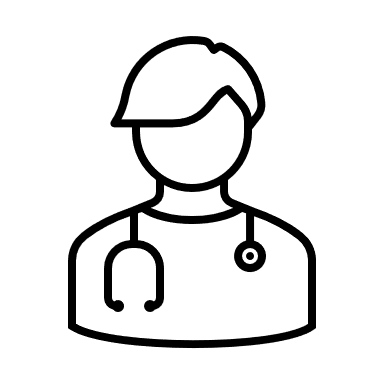


Snowball sampling

Pulmonologists

Secondary care

**Healthcare
Professionals**

Organizations

SIG Lung – KNMP,

LANA & personal network of research team


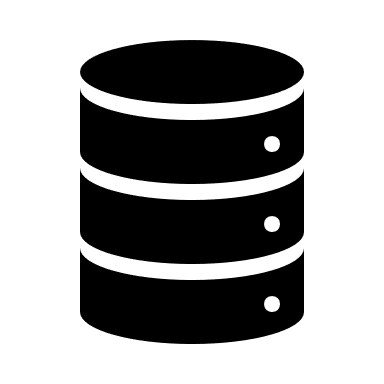

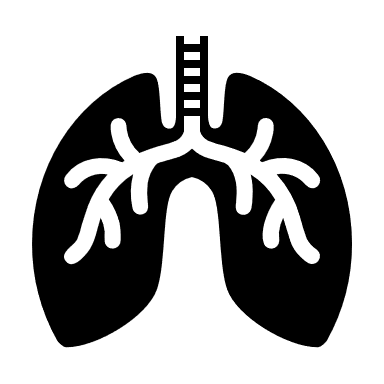

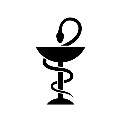


**B**

**Selection and recruitment of asthma and COPD patients**

Prior three month period


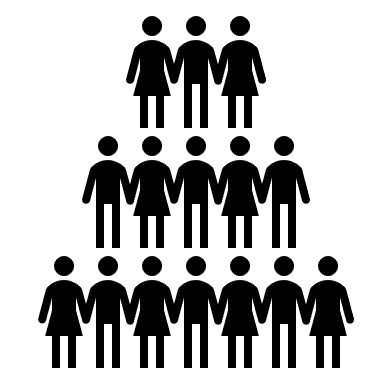

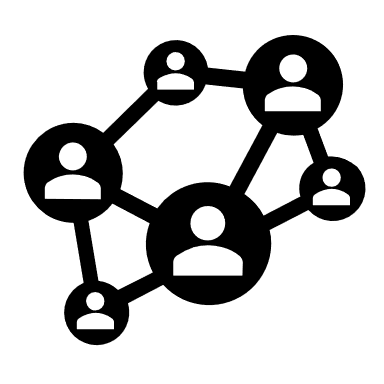

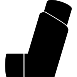


Organizations

non-profit lung

health organizations and patients’ associations

**Asthma & COPD**

**patients**

Inclusion criteria

Individuals aged >18 years & screened for asthma/COPD within pharmacy

Inclusion criteria

Patients on inhaler therapy (ATC R03)

Population base

Patient medication

dispensing data

4 community pharmacies

Selection & recruitment of patients in December 2022 in the Netherlands

**Figure A.1** **Study procedures flowchart.** Primary and secondary HCPs (GP_(A)_s, CP_(A)_s, pulmonologists) were recruited from the SIG Lung associated with the KNMP, LANA and personal network of the study team, followed by snowball sampling (A); Patients ≥ 18 years diagnosed with asthma and/or COPD who received inhaler dispenses in the prior 3-months, verified in the electronic pharmacy information system with ATC R03 codes, were purposively selected and recruited from four community pharmacies in the Netherlands. Patients were also recruited through non-profit lung health organizations and patients’ associations (B).

*Abbreviations: ATC R03: Anatomical Therapeutic Chemical code for respiratory diseases; COPD: Chronic Obstructive Pulmonary Disease; CP_(A)_: community pharmacist (assistant); GP_(A)_: general practitioner (assistant); HCP: healthcare professional; KNMP: Royal Dutch Pharmacists Association; LANA: Leiden Academic Network of Pharmacists; SIG: Special Interest Group.*

*Potential implementation strategies* based on perspectives of HCPs and patients, targeting the carbon footprint of high-emission inhalers

**B**

**A**

Suboptimal matching of inhaler devices in patients can have profound implications, for which the theory may not be feasible in clinical practice

**Figure A.2** **Thematic analyses approach.** Deductive thematic analyses (‘top-down’) was applied to provide insights into *implementation barriers and facilitators* (A) and inductive thematic analyses (‘bottom-up’) was applied to identify *potential implementation strategies* (B) on factoring environmental impact into inhaler treatment decision-making in asthma and COPD in the Netherlands.

*TDF-domains: Knowledge; Skills; Memory, Attention, Decision Process; Environmental Context and Resources; Professional Role and Identity; Beliefs about Capabilities; Beliefs about Consequences; Emotion.

*Abbreviations: COPD: Chronic Obstructive Pulmonary Disease; COM-B: Capability, Opportunity, Motivation, Behavior; DPI: dry powder inhaler; pMDI: pressurized metered dose inhaler; HCP: healthcare professional; SMI: soft mist inhaler; TDF: Theoretical Domains Framework.*

Substantiated implementation actions may increase the feasibility

of factoring environmental impact into inhaler decision-making

Perspectives of HCPs and patients concerning 8 TDF-domains*

related to ‘Capability’, ‘Opportunity’, ‘Motivation’ (COM-B model)

**Hypothesis**

**Inductive thematic analyses approach**

Identification of emerging patterns/trends (action areas) in data

Identified implementation actions may accomplish a

significant reduction in the environmental impact of inhalers

Based on insights into *implementation barriers and facilitators* to factor environmental impact into inhaler decision-making

Shift away from pMDIs and encourage DPIs or SMIs as greener option, targeting the carbon footprint of high-emission inhalers

**Deductive thematic analyses approach**

**Confirmation/Rejection of hypothesis**

**Tentative Hypothesis**

**Investigation of themes *(transcripts)***

**Theory**

**Theory**

**Patterns *(transcripts)***

**Investigation of themes *(transcripts)***

TDF-domains

*‘’Knowledge’’*

*‘’Skills’’*

*‘’Memory, Attention, Decision Process’’*

TDF-domains

*‘’Professional Role and Identity’’*

*‘’Beliefs about Capabilities’’*

*‘’Beliefs about Consequences’*

*‘’Emotion’’’*

Motivation (M)

Reflective and automatic processes that influence decision making and behavior

Capability (C)

Physical & psychological capacity to engage in behavior

**Behavior (B)**

Factoring environmental impact into inhaler treatment decision-making

Opportunity (O)

Physical and social factors which make the execution of a behavior possible

TDF-domains

*‘’Environmental Context and Resources’’*

**Figure A.3** **The applied COM-B model consisting of interacting constitutes ‘Capability’ (C), ‘Opportunity’ (O) and ‘Motivation’ (M) to generate ‘Behavior’ (B); including the accompanying Theoretical Domain Framework (TDF) domains for a granular understanding.**


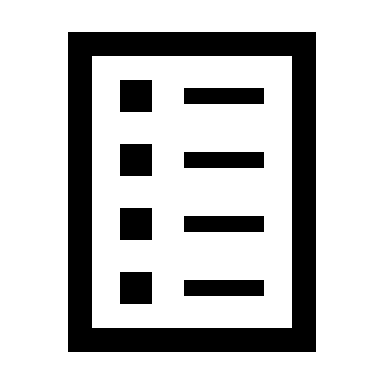

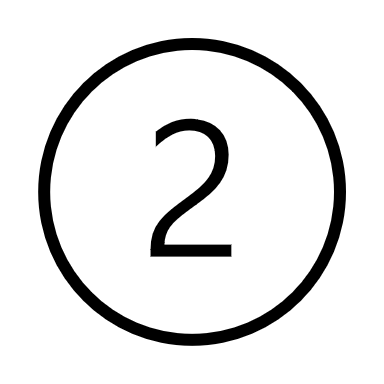

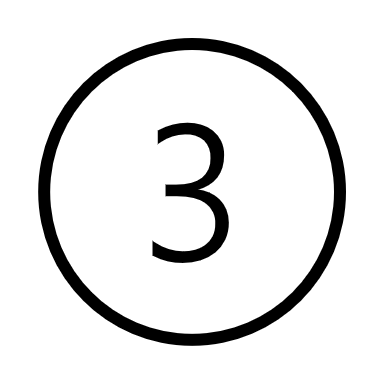

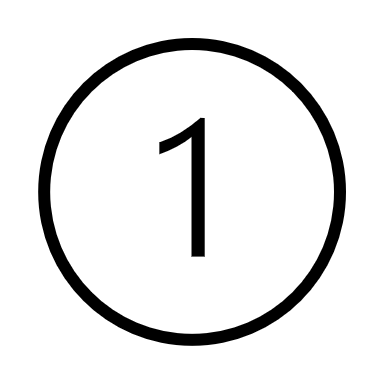

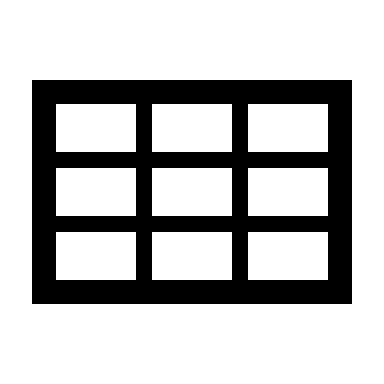

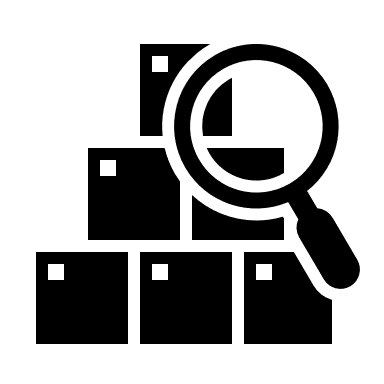


Coding scheme

(AS, NB, CV)

- COM-B constitutes

-TDF-domains/constructs

Test coding phase

(AS, NB, CV)

- initial test codings (AS, NB)

- subsequent test codings (CV)

- 2/3 transcripts per test round

Systematic literature review

(AS, NB, CV)

- patient related characteristics

- inhaler device related factors

- environmental related factors

**TEST**

**INPUT**

**OUTPUT**

**TEST ROUNDS (5x)**


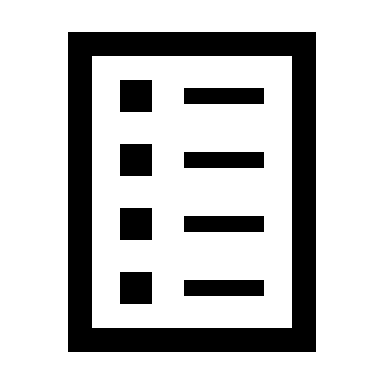

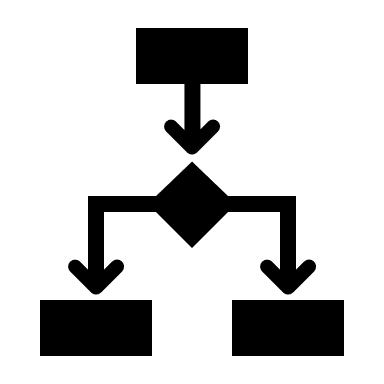

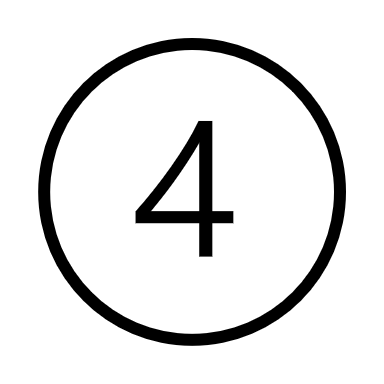


**OUTPUT**

Implementation

barriers and facilitators

8x

Transcripts

(AS, NB, CV)

8 focus group transcripts

**Deductive** thematic analysis

(finalized by CV)

- 8 focus group transcripts

- 2 coding rounds per transcript

**OUTPUT**


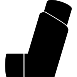

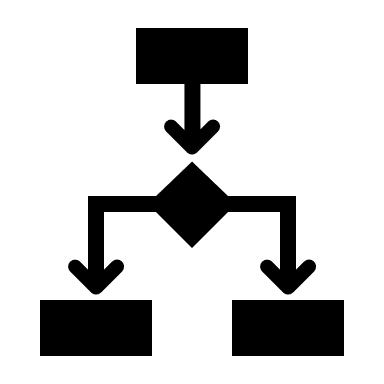

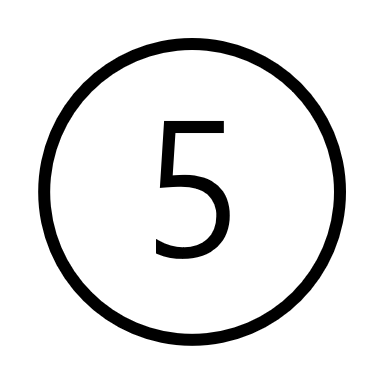


Implementation

strategies

**Figure A.4** **Overview of thematic analysis steps (1-5).** A systematic literature review (1) was conducted to acquire empirical evidence for the development of a topic guide and coding scheme (2); followed by a cyclic test coding phase on a selected number of transcripts (3); after which deductive analysis was performed on transcripts with the definitive coding scheme (4) to provide insights into implementation barriers and facilitators on environmental-friendly inhaler treatment decision-making; and inductive to identify implementation strategies (5).

*Abbreviations: COM-B: Capability, Opportunity, Motivation, Behavior; TDF: Theoretical Domains Framework.*

**Environmental-friendly inhaler treatment decision-making aid**

**Inductive** thematic analysis

(finalized by CV)

- 8 focus group transcripts

- 2 coding rounds per transcript

**ACTIONS**

# **Appendix B. Topic guides semi-structured focus groups**

The topic guides for the semi-structured focus groups (± 90 minutes) were developed by three members of the research team (AS, NR, CV) and are depicted in *table B.1* and *table* *B.2*. Input was based on empirical evidence acquired by a systematic literature review regarding patient-related, inhaler device-related and environmental-related factors influencing inhaler treatment decision-making (appendix C). Following questions were formulated revolving around 8 components of the theoretical domains framework (TDF): *Knowledge*; *Skills*; *Memory, Attention and Decision Process;* *Environmental Context and Resources; Professional Role and Identity*; *Beliefs about Capabilities*; *Beliefs about Consequences*; and *Emotion*. Prompts were based on insights of TDF based topic guides from previous studies.^1-4^ Themes, domains and order of questions highlighted in focus groups were dependent on the natural course of discussions.

**Table B.1** **Topic guide for focus groups with healthcare professionals *(translated from the original in Dutch)*.**

| **Personalized care with environmentally friendly inhaler treatment choices for asthma and COPD** | | | | |
| --- | --- | --- | --- | --- |
| **Introduction** | | | | |
| - Welcoming and introduction of participants   (name/healthcare role/healthcare organization/work experience/location)   - Provision of a brief explanation about the research - Provision of a brief explanation about the purpose and structure of the focus group - Acquire additional oral informed consent from participants for recording of the focus group | | | | |
| **General knowledge on topic** | | | | |
| 1) Have you ever participated in a group discussion before? What are your expectations from a group discussion?  2) When you think of ‘personalized care with environmentally friendly or sustainable inhalers in asthma and Chronic Obstructive Pulmonary Disease (COPD)’, what are your thoughts on this? | | | | |
| *Background on topic (Carbon footprint healthcare sector and the contribution of inhalers)* | | | | |
| **Theme 1: Experiences and preferences related to inhaler treatment choices based on patient related characteristics** | | | | |
| 3) To what extent do various/what patient characteristics determine the inhaler (device) prescribed in clinical practice? Why? What are your experiences on this?  4) How important do you believe these characteristics are when prescribing/choosing and correctly using an inhaler? Why? | | | | |
| *Prompts* | - Do you perceive differences in inhaler (device) use between patients regarding:   - (non-)actionable characteristics:     - Age (younger and older patients)?     - Co-morbidities (Tremor, rheumatism, arthritis, Parkinson etc. regarding hand-lung coordination)?     - Cognitive status?     - Educational attainment?     - Disease severity?     - Lung function?     - Inhaler therapy adherence?     - Smoking?     - Self-management?     - Etc. - To what extent are these characteristics decisive for prescribing a certain inhaler (device) type in clinical practice? - Would you like to observe reinforced attention for some patient characteristics when prescribing inhalers/for inhaler treatment choices? - Are there any differences for prescribing a certain inhaler (device) type between respiratory diseases (asthma and COPD patients)? What are they? Why? | | | |
| **Theme 2: Experiences and preferences on inhaler treatment choices based on inhaler device characteristics/design** | | | | |
| 5) To what extent do various/what inhaler device/design-related factors determine the inhaler (device) prescribed in clinical practice? Why? What are your experiences on this?  6) How important do you believe these factors are when prescribing/choosing and correctly using an inhaler? Why? | | | | |
| *Prompts* | | - Do you perceive differences in inhaler (device) use between patients regarding:   - Effectiveness?   - Side-effects?   - Number of steps/actions required for inhaling?   - Difficulty/easiness of steps/actions required for inhaling?   - Easy to learn/instruct?   - Compactness?   - Inhalation force required/inhaler device resistance?   - Hand-lung coordination?   - Dose counter?   - Hygiene?   - Use of a spacer?   - Sounds?   - Uniformity when using multiple inhalers?   - Costs/reimbursement/health insurer?   - Unidose/multidose?   - Dose frequency?   - Etc. - To what extent are these factors decisive for prescribing a certain inhaler (device) type in clinical practice? - Would you like to observe reinforced attention for some inhaler device/design related factors when prescribing inhalers/for inhaler treatment choices? - Are there any differences for prescribing a certain inhaler (device) type between respiratory diseases (asthma and COPD patients)? What are they? Why? | | |
| **Theme 3: The environmental impact of inhalers** | | | | |
| 7) To what extent do you believe inhalers contribute to the environmental impact/climate change?  8) To what extent do you consider the environmental impact in inhaler treatment choices in clinical practice? How is this implemented?  9) What opportunities or barriers do you foresee to include environmentally friendly inhaler treatment choices in clinical practice?  10) What are your experiences on switching inhalers in patients? And in particular on switching from a pressurized metered dose inhaler (pMDI) to a dry powder inhaler (DPI) or soft mist inhaler (SMI)?  11) How important do you believe these (discussed) contributing factors to the environment are when prescribing/choosing an inhaler in clinical practice? | | | | |
| *Prompts* | | | | - Which inhaler related factors do you consider to have a negative impact on the environment or may contribute to the carbon footprint? What are your thoughts on:   - The direct impact of inhalers on the environment? The carbon footprint of propellants in pMDI?   - The indirect impact of inhalers on the environment?     - The entire life cycle of an inhaler?     - The contribution of residual waste (e.g. packaging)?     - The contribution of plastic (including spacers that are replaced annually)?     - Disposable inhalers vs. reusable inhaler?     - The production of inhalers (e.g. use of raw material and water, waste)? - Starting/switching to a DPI/SMI:   - Suppose the patient is prescribed an inhaler for the first time: Would you give the preference for a DPI or SMI over a pMDI regarding the environmental impact? Why?     - What patient related characteristics or device/design-related factors would perform a barrier?   - Suppose we focus on patients who are already prescribed a pMDI: Would you consider to convert these patients with a pMDI to a DPI or SMI? Why?     - In which cases would you consider a switch and in which cases would you discourage it? Why?   - For how many patients do you believe you can achieve to start or convert to a DPI or SMI in clinical practice? Why? - Do you feel responsible as a healthcare professional to factor the environmental impact into inhaler treatment decision making? - What knowledge is currently lacking to include the environmental impact into inhaler treatment decision making? |
| **Theme 4: Supporting tools for (environmentally friendly) inhaler treatment choices** | | | | |
| 12) What are your experiences with the application of available supporting inhaler decision tools in clinical practice?  13) What do you believe would be the ideal approach to factor the environmental impact into inhaler decision-making? | | | | |
| *Prompts* | | | - What kind of supporting tools are there available? Are they frequently applied? How are they are applied? Is this sufficient? - Is shared treatment decision making applied between patients and healthcare professionals? How is this currently performed? - Are there any agreements made between healthcare professionals according to inhaler choices? What do these entail? - What kind of barriers do you experience with the currently available tools? - To what extent do you believe that discussing the environmental impact of inhalers will affect the relationship with the patient? - Is there sufficient information available on environmentally friendly inhalers (for patients and healthcare professionals)? | |
| **Closing of focus group discussion** | | | | |
| - Provision of information about the follow-up procedures of the research and focus group discussion data. - Acquire and address any remaining questions. | | | | |

**Table B.2 Topic guide for focus groups with patients *(translated from the original in Dutch)*.**

| **Personalized care with environmentally friendly inhaler treatment choices for asthma and COPD** | | | | |
| --- | --- | --- | --- | --- |
| **Introduction** | | | | |
| - Welcoming and introduction of participants   (name/age/educational status/work/respiratory disease/duration of disease/severity of disease/inhaler device use(d))   - Provision of a brief explanation about the research - Provision of a brief explanation about the purpose and structure of the focus group - Acquire additional oral informed consent from participants for recording of the focus group | | | | |
| **General knowledge on topic** | | | | |
| 1) Have you ever participated in a group discussion before? What are your expectations from a group discussion?  2) When you think of ‘personalized care with environmentally friendly or sustainable inhalers in asthma and Chronic Obstructive Pulmonary Disease (COPD)’, what are your thoughts on this? | | | | |
| *Prompts* | - Inhalers:   - When you think of inhalers, what are your thoughts on these? (e.g. various types, inhalation instruction, spacer etc.)   - Are you aware of the existence of various types of inhalers? Which inhaler types are you familiar with? - Personalized care:   - What are your thoughts on healthcare for your respiratory disease? (e.g. treatment, medication, lung function, inhalation instruction, shared treatment decision making)   - Which healthcare professionals are involved in keeping your respiratory disease under control?   - What are your thoughts on personalized care with regard to your inhaler? - Environmentally friendly/sustainability:   - What comes to mind when hearing the words ‘climate-friendly’, ‘sustainability’ or ‘environmental impact’?   - What are your thoughts on these words in relation to inhalers? (e.g. residual waste, plastic, air pollution, carbon footprint)   - Are you aware that inhalers are harmful to the environment and can contribute to the carbon footprint? What do you know about this topic? Would you like to learn more about this topic?   - Are you aware that propellants in pressurized metered dose inhalers (pMDIs) are greenhouse gases which contribute to the carbon footprint? | | | |
| *Background on topic (Carbon footprint healthcare sector and the contribution of inhalers)* | | | | |
| **Theme 1: Experiences and preferences on inhaler device characteristics/design** | | | | |
| 3) What are your experiences on using your current and/or previous inhaler(s)?  4) How important do you believe the inhaler device characteristics/design are for choosing and correctly using an inhaler?  5) If you could rate each of these attributable factors for choosing and correctly using an inhaler from 1 (most important) to 10 (least important). What would be your order of importance? Why? | | | | |
| *Prompts* | | - How do you feel about the use of your inhaler?   - What do you like about (the use of) your inhaler? What are you satisfied with?   - What do you find difficult about using your inhaler? What do you believe is the cause for this?   - On what aspects would you like to observe reinforced attention in inhaler treatment choices? - What do you consider important in (using) an inhaler? Why? What are your experiences on this?   - Effectiveness?   - Side-effects? (e.g. gargling and rinsing mouth with water after inhaling to prevent or decrease side-effects, effects on adherence)   - Number of steps/actions required for inhaling?   - Difficulty/easiness of steps/actions required for inhaling? (e.g. preparation of inhaler, hand-lung coordination, inhalation force required, different settings (un)controlled disease)   - Easy to learn/instruct? (e.g. dependent on inhalation-instruction, device feedback)   - Compactness? (e.g. use in public, traveling, work)   - Inhalation force required?   - Hand-lung coordination?   - Dose counter?   - Hygiene?   - Use of a spacer?   - Sounds?   - Uniformity when using multiple inhalers?   - Costs/reimbursement/health insurer?   - Unidose/multidose?   - Number of inhaler dosages per day? (e.g. twice daily or once daily with controller therapy)   - Etc. - To what extent have you noticed any differences in inhaler (devices) that you use or have used? (e.g. pMDI versus dry powder inhaler (DPI) or soft mist inhaler (SMI)) What are your experiences on this? (e.g. barriers/facilitators) - Wat are your experiences on having to switch inhaler (device) and the different inhalation skills required per inhaler device? - Which type of inhaler would you prefer when considering your previous and current inhaler experiences or knowledge on the different inhaler (devices)? (e.g. pMDI, DPI or SMI) Why? | | |
| **Theme 2: The environmental impact of inhalers** | | | | |
| 6) To what extent do you believe inhalers contribute to the environmental impact/climate change?  7) How important is the environmental impact of inhalers for you? Why?  8) To what extent do you consider the environmental impact to be important in inhaler treatment choices?  9) If you would add the environmental impact/sustainability of inhalers to the beforementioned attributable factors for choosing and correctly using an inhaler from 1 (most important) to 10 (least important), were would you put this factor? Why? | | | | |
| *Prompts* | | | | - Which inhaler related factors do you consider to have a negative impact on the environment or may contribute to the carbon footprint? What are your thoughts on:   - The direct impact of inhalers on the environment? The carbon footprint of propellants in pMDI?   - The indirect impact of inhalers on the environment?     - The entire life cycle of an inhaler?     - The contribution of residual waste (e.g. packaging)?     - The contribution of plastic (including spacers that are replaced annually)?     - Disposable inhalers vs. reusable inhaler?     - The production of inhalers (e.g. use of raw material and water, waste)? - To what extent is the environmental impact or sustainability of inhalers taken into account as factor in inhaler treatment decision making by your healthcare professional(s)? Are you involved in this choice? Would you like to be involved? - How motivated would you be to switch to environmentally friendly inhalers? Why?   - Switch from pMDI to DPI or SMI?   - Switch from pMDI with a high impact propellant to a lower impact propellant?   - Switch to reusable inhalers?   - Switch according to inhalers with low greenhouse gas (GHG) emissions throughout the entire life cycle? - What inhaler device characteristics/design would you be willing to sacrifice in order to switch to an environmentally friendly inhaler? - Do you feel responsible to take the environmental impact into consideration within the inhaler treatment decision making process and the use of inhalers? - What knowledge is currently lacking or would you like to receive from your healthcare professional to include the environmental impact into inhaler treatment decision making? Why? |
| **Theme 3: Shared (environmentally friendly) treatment inhaler decision making** | | | | |
| 10) To what extent were you involved in the choice of your inhaler (device)?  11) Should the environmental impact be taken into consideration in inhaler treatment decision making? How and why? | | | | |
| *Prompts* | | | - Is shared treatment decision making applied between patients and healthcare professionals? How was this performed?   - Have you had the opportunity to choose a particular type of inhaler?   - How much influence did you have on the choice of inhaler (device)? - Were the various inhaler device characteristics discussed between you and your healthcare professional when choosing an inhaler (device)?   - Have your wishes, needs and preferences been taken into account?   - What characteristics were not addressed which you would like reinforced attention on in inhaler treatment choices? - To what extent do you think it is important to be able to choose your own inhaler (device)? Why?   - Have you ever tried to suggest a different inhaler (device) than the inhaler prescribed or dispensed by your doctor/pharmacist? Why?   - Have you experienced not receiving the prescribed inhaler from your doctor/pharmacist?   - How satisfied are you with your current inhaler? - Do you receive feedback from your healthcare professional(s) on how well you use your inhaler?   - How often is the chosen inhaler reviewed?   - What are your wishes and needs on this? - Is there sufficient information available on environmentally friendly inhalers? | |
| **Closing of focus group discussion** | | | | |
| - Provision of information about the follow-up procedures of the research and focus group discussion data. - Acquire and address any remaining questions. | | | | |

^1^Presseau J, Schwalm JD, Grimshaw JM, Witteman HO, Natarajan MK, Linklater S, Sullivan K, Ivers NM. Identifying determinants of medication adherence following myocardial infarction using the Theoretical Domains Framework and the Health Action Process Approach. Psychol Health. 2017 Oct;32(10):1176-1194. doi: 10.1080/08870446.2016.1260724. Epub 2016 Dec 20. PMID: 27997220.

^2^Lawton R, Heyhoe J, Louch G, Ingleson E, Glidewell L, Willis TA, McEachan RR, Foy R; ASPIRE programme. Using the Theoretical Domains Framework (TDF) to understand adherence to multiple evidence-based indicators in primary care: a qualitative study. Implement Sci. 2016 Aug 8;11:113. doi: 10.1186/s13012-016-0479-2. PMID: 27502590; PMCID: PMC4977705.

^3^Visser CD, Faay MRA, Özdemir A, Guchelaar HJ, Teichert M. Short-acting β2-agonists (SABA) overuse in asthma and patients' perceptions for this behavior. Respir Med. 2024 Sep;231:107723. doi: 10.1016/j.rmed.2024.107723. Epub 2024 Jun 25. PMID: 38936636.

^4^Visser CD, Linthorst JM, Kuipers E, Sont JK, Lacroix JPW, Guchelaar HJ, Teichert M. Respiratory Adherence Care Enhancer Questionnaire: Identifying Self-Management Barriers of Inhalation Corticosteroids in Asthma. Front Pharmacol. 2021 Dec 22;12:767092. doi: 10.3389/fphar.2021.767092. PMID: 35002706; PMCID: PMC8729223.

# **Appendix C. Search strategy systematic literature review**

Three consecutive systematic literature searches have been performed to acquire empirical evidence on patient-related, inhaler device-related and environmental-related factors influencing inhaler treatment decision-making in asthma and COPD. The search was conducted in PubMed using a combination of keywords and Medical Subject Headings (MeSH), as depicted in *table C.1*. Retrieved articles were screened on title, abstract and full-text. Commentaries, editorials, poster abstracts and publications in languages other than English or Dutch were excluded. Any discrepancies were resolved through discussions.

| **Table C.1 Search strategy to acquire empirical evidence on factors influencing inhaler decision-making.**  **Search strategy 1: Patient-related characteristics influencing inhaler treatment decision making** | | | | | |
| --- | --- | --- | --- | --- | --- |
| Reseach question | | Which patient characteristics (of asthma and COPD patients) are compatible with inhaler device skills? | | | |
| (("Asthma"[majr] OR "Asthma"[ti] OR "Asthma*"[ti] OR "Bronchial asthma"[ti] OR "Respiratory Hypersensitivity"[majr:NoExp] OR "Respiratory Hypersensitivity"[ti] OR "Pulmonary Disease, Chronic Obstructive"[majr] OR "Lung Diseases, Obstructive"[majr:noexp] OR "Chronic Obstructive Lung Disease"[ti] OR "Chronic Obstructive Pulmonary Diseases"[ti] OR "COPD"[ti] OR "Chronic Obstructive Airway Disease"[ti] OR "Chronic Obstructive Pulmonary Disease"[ti] OR "Chronic Airflow Obstructions"[ti] OR "Chronic Airflow Obstruction"[ti] OR "Obstructive Lung Disease"[ti] OR "Obstructive Pulmonary Diseases"[ti] OR "Obstructive Pulmonary Disease"[ti] OR "Obstructive Lung Diseases"[ti]) AND ("Dry Powder Inhalers"[majr] OR "Metered Dose Inhalers"[majr] OR "Powders"[majr] OR "Aerosols"[majr] OR "Nebulizers and Vaporizers"[majr] OR "soft mist inhaler"[ti] OR "Dry Powder Inhaler"[ti] OR "Dry Powder Inhalers"[ti] OR "Metered Dose Inhaler"[ti] OR "MDI"[ti] OR "DPI"[ti] OR "SMI"[ti] OR "Spacer Inhalers"[ti] OR "Spacer Inhaler"[ti] OR "Spacer-Inhalers"[ti] OR "Spacer-Inhaler"[ti] OR "Aerosol"[ti] OR "Vaporizers and Nebulizers"[ti] OR "Vaporizers"[ti] OR "Vaporizer"[ti] OR "Vaporisers"[ti] OR "Vaporiser"[ti] OR "Inhalers"[ti] OR "Inhaler"[ti] OR "Inhalators"[ti] OR "Inhalator"[ti] OR "Nebulizers"[ti] OR "Nebulizer"[ti] OR "Nebulisers"[ti] OR "Nebuliser"[ti] OR "Atomizers"[ti] OR "Atomizer"[ti] OR "Inhalation Devices"[ti] OR "Inhalation Device"[ti] OR "Inhalation Spacer"[ti] OR "Inhalation Spacers"[ti]) AND ("inhaler skill"[tw] OR "inhaler skills"[tw] OR "device skill"[tw] OR "device skills"[tw] OR "inhaler technique"[tw] OR "inhaler techniques"[tw] OR "device technique"[tw] OR "device techniques"[tw] OR "skills"[ti] OR "skill"[ti] OR "skilled"[ti] OR "Motor Skills"[Mesh]) NOT (("Infant"[mesh] OR "infant"[ti] OR "infants"[ti] OR "Child"[mesh] OR "child"[ti] OR "children"[ti] OR "girl"[ti] OR "girls"[ti] OR "boy"[ti] OR "boys"[ti] OR "Adolescent"[mesh] OR "adolescent"[ti] OR "adolescents"[ti] OR "pediatric"[ti] OR "pediatrics"[ti] OR "paediatric"[ti] OR "paediatrics"[ti]) NOT ("Adult"[mesh] OR "adult"[ti] OR "adults"[ti] OR "middle aged"[ti] OR "elderly"[ti])) AND ("2012/01/01"[PDAT] : "3000/12/31"[PDAT])) | | | | | |
| Articles retrieved | | 337 references on 15-11-2022 | | | |
| **Search strategy 2: Inhaler device-related factors influencing inhaler treatment decision making** | | | | | |
| Research question | What are asthma and COPD patients’ and healthcare providers’ perspectives, preferences, and experiences on inhaler devices on the market? | | | | |
| (("Asthma"[mesh] OR "Asthma"[tw] OR "Asthma*"[tw] OR "Bronchial asthma"[tw] OR "Respiratory Hypersensitivity"[Mesh:NoExp] OR "Respiratory Hypersensitivity"[tw] OR "Pulmonary Disease, Chronic Obstructive"[Mesh] OR "Lung Diseases, Obstructive"[Mesh:noexp] OR "Chronic Obstructive Lung Disease"[tw] OR "Chronic Obstructive Pulmonary Diseases"[tw] OR "COPD"[tw] OR "Chronic Obstructive Airway Disease"[tw] OR "Chronic Obstructive Pulmonary Disease"[tw] OR "Chronic Airflow Obstructions"[tw] OR "Chronic Airflow Obstruction"[tw] OR "Obstructive Lung Disease"[tw] OR "Obstructive Pulmonary Diseases"[tw] OR "Obstructive Pulmonary Disease"[tw] OR "Obstructive Lung Diseases"[tw]) AND ("Dry Powder Inhalers"[mesh] OR "Metered Dose Inhalers"[mesh] OR "Powders"[mesh] OR "Aerosols"[mesh] OR "Nebulizers and Vaporizers"[mesh] OR "soft mist inhaler"[tw] OR "Dry Powder Inhaler"[tw] OR "Dry Powder Inhalers"[tw] OR "Metered Dose Inhaler"[tw] OR "MDI"[ti] OR "DPI"[tw] OR "SMI"[tw] OR "Spacer Inhalers"[tw] OR "Spacer Inhaler"[tw] OR "Spacer-Inhalers"[tw] OR "Spacer-Inhaler"[tw] OR "Aerosol"[tw] OR "Vaporizers and Nebulizers"[tw] OR "Vaporizers"[tw] OR "Vaporizer"[tw] OR "Vaporisers"[tw] OR "Vaporiser"[tw] OR "Inhalers"[tw] OR "Inhaler"[tw] OR "Inhalators"[tw] OR "Inhalator"[tw] OR "Nebulizers"[tw] OR "Nebulizer"[tw] OR "Nebulisers"[tw] OR "Nebuliser"[tw] OR "Atomizers"[tw] OR "Atomizer"[tw] OR "Inhalation Devices"[tw] OR "Inhalation Device"[tw] OR "Inhalation Spacer"[tw] OR "Inhalation Spacers"[tw]) AND ("Patient Preference"[mesh] OR "Patient Satisfaction"[mesh] OR "Patient Preference"[tw] OR "Patient Preferences"[tw] OR "Patient Satisfaction"[tw] OR "Patient Perspective"[tw] OR "Patient Perspectives"[tw] OR "Patient Experience"[tw] OR "Patient Experiences"[tw] OR "Patients Preference"[tw] OR "Patients Preferences"[tw] OR "Patients Satisfaction"[tw] OR "Patients Perspective"[tw] OR "Patients Perspectives"[tw] OR "Patients Experience"[tw] OR "Patients Experiences"[tw] OR "Patient's Preference"[tw] OR "Patient's Preferences"[tw] OR "Patient's Satisfaction"[tw] OR "Patient's Perspective"[tw] OR "Patient's Perspectives"[tw] OR "Patient's Experience"[tw] OR "Patient's Experiences"[tw] OR "Patients' Preference"[tw] OR "Patients' Preferences"[tw] OR "Patients' Satisfaction"[tw] OR "Patients' Perspective"[tw] OR "Patients' Perspectives"[tw] OR "Patients' Experience"[tw] OR "Patients' Experiences"[tw] OR "Patient Perception"[tw] OR "Patient Perceptions"[tw] OR "Patients Perception"[tw] OR "Patients Perceptions"[tw] OR "Patient's Perception"[tw] OR "Patient's Perceptions"[tw] OR "Patients' Perception"[tw] OR "Patients' Perceptions"[tw] OR "Physician Preference"[tw] OR "Physician Preferences"[tw] OR "Physician Satisfaction"[tw] OR "Physician Perspective"[tw] OR "Physician Perspectives"[tw] OR "Physician Experience"[tw] OR "Physician Experiences"[tw] OR "Physicians Preference"[tw] OR "Physicians Preferences"[tw] OR "Physicians Satisfaction"[tw] OR "Physicians Perspective"[tw] OR "Physicians Perspectives"[tw] OR "Physicians Experience"[tw] OR "Physicians Experiences"[tw] OR "Physician's Preference"[tw] OR "Physician's Preferences"[tw] OR "Physician's Satisfaction"[tw] OR "Physician's Perspective"[tw] OR "Physician's Perspectives"[tw] OR "Physician's Experience"[tw] OR "Physician's Experiences"[tw] OR "Physicians' Preference"[tw] OR "Physicians' Preferences"[tw] OR "Physicians' Satisfaction"[tw] OR "Physicians' Perspective"[tw] OR "Physicians' Perspectives"[tw] OR "Physicians' Experience"[tw] OR "Physicians' Experiences"[tw] OR "Doctor Preference"[tw] OR "Doctor Preferences"[tw] OR "Doctor Satisfaction"[tw] OR "Doctor Perspective"[tw] OR "Doctor Perspectives"[tw] OR "Doctor Experience"[tw] OR "Doctor Experiences"[tw] OR "Doctors Preference"[tw] OR "Doctors Preferences"[tw] OR "Doctors Satisfaction"[tw] OR "Doctors Perspective"[tw] OR "Doctors Perspectives"[tw] OR "Doctors Experience"[tw] OR "Doctors Experiences"[tw] OR "Doctor's Preference"[tw] OR "Doctor's Preferences"[tw] OR "Doctor's Satisfaction"[tw] OR "Doctor's Perspective"[tw] OR "Doctor's Perspectives"[tw] OR "Doctor's Experience"[tw] OR "Doctor's Experiences"[tw] OR "Doctors' Preference"[tw] OR "Doctors' Preferences"[tw] OR "Doctors' Satisfaction"[tw] OR "Doctors' Perspective"[tw] OR "Doctors' Perspectives"[tw] OR "Doctors' Experience"[tw] OR "Doctors' Experiences"[tw] OR "Provider Preference"[tw] OR "Provider Preferences"[tw] OR "Provider Satisfaction"[tw] OR "Provider Perspective"[tw] OR "Provider Perspectives"[tw] OR "Provider Experience"[tw] OR "Provider Experiences"[tw] OR "Providers Preference"[tw] OR "Providers Preferences"[tw] OR "Providers Satisfaction"[tw] OR "Providers Perspective"[tw] OR "Providers Perspectives"[tw] OR "Providers Experience"[tw] OR "Providers Experiences"[tw] OR "Provider's Preference"[tw] OR "Provider's Preferences"[tw] OR "Provider's Satisfaction"[tw] OR "Provider's Perspective"[tw] OR "Provider's Perspectives"[tw] OR "Provider's Experience"[tw] OR "Provider's Experiences"[tw] OR "Providers' Preference"[tw] OR "Providers' Preferences"[tw] OR "Providers' Satisfaction"[tw] OR "Providers' Perspective"[tw] OR "Providers' Perspectives"[tw] OR "Providers' Experience"[tw] OR "Providers' Experiences"[tw] OR "Practitioner Preference"[tw] OR "Practitioner Preferences"[tw] OR "Practitioner Satisfaction"[tw] OR "Practitioner Perspective"[tw] OR "Practitioner Perspectives"[tw] OR "Practitioner Experience"[tw] OR "Practitioner Experiences"[tw] OR "Practitioners Preference"[tw] OR "Practitioners Preferences"[tw] OR "Practitioners Satisfaction"[tw] OR "Practitioners Perspective"[tw] OR "Practitioners Perspectives"[tw] OR "Practitioners Experience"[tw] OR "Practitioners Experiences"[tw] OR "Practitioner's Preference"[tw] OR "Practitioner's Preferences"[tw] OR "Practitioner's Satisfaction"[tw] OR "Practitioner's Perspective"[tw] OR "Practitioner's Perspectives"[tw] OR "Practitioner's Experience"[tw] OR "Practitioner's Experiences"[tw] OR "Practitioners' Preference"[tw] OR "Practitioners' Preferences"[tw] OR "Practitioners' Satisfaction"[tw] OR "Practitioners' Perspective"[tw] OR "Practitioners' Perspectives"[tw] OR "Practitioners' Experience"[tw] OR "Practitioners' Experiences"[tw] OR "Physician Perception"[tw] OR "Physician Perceptions"[tw] OR "Physicians Perception"[tw] OR "Physicians Perceptions"[tw] OR "Physician's Perception"[tw] OR "Physician's Perceptions"[tw] OR "Physicians' Perception"[tw] OR "Physicians' Perceptions"[tw] OR "Doctor Perception"[tw] OR "Doctor Perceptions"[tw] OR "Doctors Perception"[tw] OR "Doctors Perceptions"[tw] OR "Doctor's Perception"[tw] OR "Doctor's Perceptions"[tw] OR "Doctors' Perception"[tw] OR "Doctors' Perceptions"[tw] OR "Provider Perception"[tw] OR "Provider Perceptions"[tw] OR "Providers Perception"[tw] OR "Providers Perceptions"[tw] OR "Provider's Perception"[tw] OR "Provider's Perceptions"[tw] OR "Providers' Perception"[tw] OR "Providers' Perceptions"[tw] OR "Practitioner Perception"[tw] OR "Practitioner Perceptions"[tw] OR "Practitioners Perception"[tw] OR "Practitioners Perceptions"[tw] OR "Practitioner's Perception"[tw] OR "Practitioner's Perceptions"[tw] OR "Practitioners' Perception"[tw] OR "Practitioners' Perceptions"[tw] OR (("Life Course Perspective"[Mesh] OR "Perception"[Mesh] OR "Clinical Reasoning"[Mesh] OR "preference"[ti] OR "preferences"[ti] OR "Life Course Perspective"[ti] OR "Life Course Perspectives"[ti] OR "Perception"[ti] OR "perceptions"[ti] OR "Clinical Reasoning"[ti] OR "Clinical Judgment"[ti] OR "Clinical Judgments"[ti] OR "Reasoning"[ti] OR "Reason"[ti] OR "Reasons"[ti] OR "experience"[ti] OR "experiences"[ti]) AND ("Patients"[Mesh] OR "Patients"[tw] OR "Patient"[tw] OR "Health Personnel"[Mesh] OR "Physician"[tw] OR "Physicians"[tw] OR "Doctor"[tw] OR "Doctors"[tw] OR "Provider"[tw] OR "Provider"[tw] OR "Practitioner"[tw] OR "Practitioners"[tw]) AND ("Dry Powder Inhalers"[majr] OR "Metered Dose Inhalers"[majr] OR "Powders"[majr] OR "Aerosols"[majr] OR "Nebulizers and Vaporizers"[majr] OR "soft mist inhaler"[ti] OR "Dry Powder Inhaler"[ti] OR "Dry Powder Inhalers"[ti] OR "Metered Dose Inhaler"[ti] OR "MDI "[ti] OR "DPI"[ti] OR "SMI"[ti] OR "Spacer Inhalers"[ti] OR "Spacer Inhaler"[ti] OR "Spacer-Inhalers"[ti] OR "Spacer-Inhaler"[ti] OR "Aerosol"[ti] OR "Vaporizers and Nebulizers"[ti] OR "Vaporizers"[ti] OR "Vaporizer"[ti] OR "Vaporisers"[ti] OR "Vaporiser"[ti] OR "Inhalers"[ti] OR "Inhaler"[ti] OR "Inhalators"[ti] OR "Inhalator"[ti] OR "Nebulizers"[ti] OR "Nebulizer"[ti] OR "Nebulisers"[ti] OR "Nebuliser"[ti] OR "Atomizers"[ti] OR "Atomizer"[ti] OR "Inhalation Devices"[ti] OR "Inhalation Device"[ti] OR "Inhalation Spacer"[ti] OR "Inhalation Spacers"[ti] OR "device"[ti] OR "devices"[ti]))) NOT (("Infant"[mesh] OR "infant"[ti] OR "infants"[ti] OR "Child"[mesh] OR "child"[ti] OR "children"[ti] OR "girl"[ti] OR "girls"[ti] OR "boy"[ti] OR "boys"[ti] OR "Adolescent"[mesh] OR "adolescent"[ti] OR "adolescents"[ti] OR "pediatric"[ti] OR "pediatrics"[ti] OR "paediatric"[ti] OR "paediatrics"[ti]) NOT ("Adult"[mesh] OR "adult"[ti] OR "adults"[ti] OR "middle aged"[ti] OR "elderly"[ti])) AND ("2012/01/01"[PDAT] : "3000/12/31"[PDAT])) | | | | | |
| Articles retrieved | | | | 514 references on 15-11-2022 | |
| **Search strategy 3: Environmental related factors influencing inhaler treatment decision making** | | | | | |
| Research question | | | What is the contribution of pMDI, SMI and DPI devices on carbon footprint? (pressurized metered dose inhalers, soft mist inhalers and dry powder inhalers)? | | |
| (("Dry Powder Inhalers"[mesh] OR "Metered Dose Inhalers"[mesh] OR "Nebulizers and Vaporizers"[mesh] OR "soft mist inhaler"[tw] OR "Dry Powder Inhaler"[tw] OR "Dry Powder Inhalers"[tw] OR "Metered Dose Inhaler"[tw] OR "Spacer Inhalers"[tw] OR "Spacer Inhaler"[tw] OR "Spacer-Inhalers"[tw] OR "Spacer-Inhaler"[tw] OR "Vaporizers and Nebulizers"[tw] OR "Inhalers"[tw] OR "Inhaler"[tw] OR "Inhalators"[tw] OR "Inhalator"[tw] OR "Inhalation Devices"[tw] OR "Inhalation Device"[tw] OR "Inhalation Spacer"[tw] OR "Inhalation Spacers"[tw]) AND ("Carbon Footprint"[Mesh] OR "Conservation of Natural Resources"[Mesh] OR "Greenhouse Effect"[Mesh] OR "Global Warming"[Mesh] OR "carbon footprint"[tw] OR "carbon footprints"[tw] OR "natural resources"[tw] OR "natural resource"[tw] OR "greenhouse"[tw] OR "global warming"[tw] OR "Environmental Pollution"[Mesh] OR "environmental pollution"[tw] OR "environmental pollutions"[tw] OR "Environmental Pollutants"[Mesh] OR "Environmental Pollutants"[Pharmacological Action] OR "environmental pollutant"[tw] OR "environmental pollutants"[tw] OR "Medical Waste"[Mesh] OR "Medical Waste"[tw] OR "Greenhouse Gases"[Mesh] OR "Greenhouse Gases"[tw] OR "Greenhouse Gas"[tw] OR "waste"[tw] OR "wastes"[tw] OR "wasting"[tw] OR "wastage"[tw] OR "wastages"[tw] OR "environmental impact"[tw] OR "environmental impact*"[tw] OR "environmental burden"[tw] OR "environmental burden*"[tw] OR ("dispos*"[tw] AND ("reuse"[tw] OR "re use"[tw] OR "reusab*"[tw] OR "re usabl*"[tw]))) AND ("2012/01/01"[PDAT] : "3000/12/31"[PDAT])) | | | | | |
| Articles retrieved | | | | | 208 references on 15-11-2022 |
| **Total articles retrieved** | | | | | **746 references** |

# **Appendix D. Coding scheme qualitative data**

A reaction pattern, involving experiential, behavioral and physiological elements; by which the individual attempts

to deal with a personally significant event.

Beliefs about capabilities

Beliefs about consequences

Emotion

The ability to focus selectively and choose between ≥ 2 alternatives (ideally/in principle)

Memory, attention, decision process

Skills (physical and cognitive)

The external (physical and social) factors which make the execution of a behavior possible

The internal (reflective and automatic) processes that influence decision-making and behavior

The (physical and psychological) capacity to engage in the concerned activity/behavior *(ideally/in principle)*

From the perspective of:

- Primary HCPs

- Secondary HCPs

- Asthma and COPD patients

*Abbreviations: COM-B: Capability, Opportunity, Motivation, Behavior; COPD: Chronic Obstructive Pulmonary Disease; DPI: dry powder inhaler; pMDI: pressurized metered dose inhaler; HCP: healthcare professional; SDM: Shared Decision Making; SMI: soft mist inhaler; TDF: Theoretical Domains Framework.*

The ability and competence required

for optimal inhaler use

**Figure D.1** **Deductive coding scheme for identification of implementation challenges/facilitators to factor environmental impact into inhaler treatment decision-making.**

Social

Circumstances or environment that discourages/encourages this behavior

Acceptance or reality about negative outcomes of this behavior in a given situation

Acceptance or reality about the ability of

this behavior in a constructive way

A coherent set of personal qualities of an individual in a social (society) or work setting

Awareness of the existence of

the environmental impact of inhalers

*Influences*

*Influences*

Psychological

**Factoring environmental impact into inhaler treatment decision-making**

- Environmental stressors: e.g. misinformation; time restraints;

availability, preference policy; costs; language barrier

- (Material) resources; (evidence-based) educational material

Reaction patterns:

- Positive association e.g. enthusiasm, interest, alertness;

- Negative association e.g. shock, fear, anxiety, worry, distress

- Cognitive dissonance e.g. in climate beliefs and actions.

Environmental context and resources

- Anticipated regret: risk aversion, reversibility of actions

- Poor outcome expectancies: adherence, self-management,

disease control; patient-HCP relationship.

- Responsibility to factor the environmental impact into inhaler

decision-making; set duties/ethics; leadership; humanitarian

- Potential (organizational) role; role in tackling climate change

- Perceived competence: perception on capacity to complete

specific task(s); behavioral control; rewards, praise, feedback

- Confidence to combat climate change: trust and/or personal

belief in ability; positive outcomes; significance; persistence

- Current behavior regarding (environmental-friendly) inhaler

decision making; e.g. inhaler superiority, habit, actions taken.

- Importance of SDM; e.g. willingness/attitude to start/switch

due to perspectives, preferences, wishes/needs

- Prioritization of sustainable inhaler actions

- Inhaler device ability and competence: Patient/inhaler device

related characteristics enabling and/or restricting (optimal)

inhaler use/switch; critical inhaler skills; (dis)advantages.

- Skill development, ability, competence, practice: Direct

inhaler feedback systems; regular inhaler technique

education; supporting tools/services/electronic monitors

- Awareness/acquainted with environmental impact of inhalers;

e.g. through self-informed knowledge or training.

- Multitude of elements: R-ladder ‘reduce, re-use, recycle’

- (In)complete critical information to factor the environmental

impact into inhaler decision-making.

**CONSTRUCTS FOR CHALLENGES/FACILITATORS**

Physical

**Capability**

**Opportunity**

Automatic

Reflective

**Motivation**

Physical

Professional role and identity

Knowledge

**TDF-DOMAINS**

**COM-B MODEL (SUB-CONSTRUCTS)**

**BEHAVIOR**

**APPROACH**

**IDENTIFIED ACTION AREAS**

**CONCEPTUAL STRATEGY ELEMENTS**

Education to facilitate sustainable

inhaler prescribing and healthcare

**1. Communication, education, awareness**

Building sustainable relationships

Return inhalers at community pharmacy

Prescribe inhalers only if

clinically indicated

Discuss and agree on sustainable actions

within regional/national healthcare setting

Inhaler incineration prevents release of harmful GHGs into

the environment and recycling reduces the need for

extracting, refining and processing raw materials

Improvement of inhaler technique and self-management, reducing unplanned care and (medication) waste

Reducing unnecessary inhaler prescribing reduces the environmental impact related with their use

Seeking to inform and educate HCPs and patients

about sustainability with the intention to facilitate environmental-friendly inhaler decision-making

Optimizing effective

pharmacotherapy management

Focusing on the prescription of

environmental-friendly inhaler alternatives

*Abbreviations: COPD: Chronic Obstructive Pulmonary Disease; DPI: dry powder inhaler; pMDI: pressurized metered dose inhaler; HCP: healthcare professional; HFC: hydrofluorocarbon; GHG: greenhouse gas; GWP: global warming potential; SMART: Single Maintenance and Reliever Therapy; SMI: soft mist inhaler.*

**Figure D.2** **Inductive coding scheme for the identification of potential implementation strategies to factor environmental impact into inhaler treatment decision-making.**

Set-up of an inhaler recycling scheme

Regular training and educational efforts

for HCPs

Consider the lowest inhalation frequency

**3. Promotion of smarter inhaler choices**

Combination inhaler vs. single inhalers

Encourage re-usable inhalers

Encourage the use of a dose-counter

Select pMDI with lowest GWP HFC

Start or switch to DPIs if appropriate

By choosing the most suitable inhaler for each individual, adherence and clinical outcomes may be improved, reducing unplanned care & (medication) waste

**Direct approach**

**Indirect approach**

**5. Appropriate inhaler disposal**

**4. Optimization of quality of care**

Tackle self-management barriers

Perform regular inhalation instructions

Encourage use of a spacer with a pMDI

Lower dose prescribing if appropriate

Tackle over/underuse inhaler therapy

Consider ‘SMART’ therapy in asthma

**2. Appropriate inhaler prescribing**

Monitoring and evaluating prescription

behavior and impact of interventions

**Appendix E. Perceptions of participants from focus group discussions** *(translated from original in Dutch)*

| **Participant** | **Quotes**  **Table E.1 Perceptions on challenges and facilitators to factor environmental impact into inhaler decision-making identified in focus group discussions; integrated under COM-B model, TDF-domains and constructs.** | **Construct*** |
| --- | --- | --- |
| **CAPABILITY** | | |
| **TDF-domain ‘’Knowledge’’** | | |
| GP_1_ | *‘’I believe the information is clear but not everyone is up-to-date. I think that could use more attention’’* | 1.1 |
| GP_1_ | *‘’…next week I will organize a course on sustainability, a PTAM on sustainability in our practice’’* | 1.1 |
| GP_2_ | *‘’When I think about sustainability, I think about prescriptions, what kind of medication to prescribe. We have been looking at pMDI and DPI for some time now, so regarding sustainability those pMDIs are more burdensome’’* | 1.1 |
| GP_2_ | *‘’We also try to perform [sustainable] actions in practice such as the sorting of paper, those kind of aspects, we are really working on that, sorting used medicines’’* | 1.1 |
| GP_2_ | *‘’I am a GP trainer and at the two-day GP trainers event in the autumn, we received a talk from the ‘green’ GP about what he has implemented in his practice to become carbon neutral. I believe they plant half a forest each year to become neutral, but yes many different aspects, sorting of paper, monitoring medication use but also e.g. having medication and instruments delivered or stock replenished once a week instead of three times a week, that also saves kilometres…so it does receive more attention’’* | 1.1 |
| GP_3_ | *‘’I am a trainer and there was a two-day event about sustainability and all sorts of aspects in practice e.g. that you could incorporate sustainability into how you travel to the practice…there were GPs who had built bike sheds and of course you have people who put solar panels on their roofs or who separate paper waste. I have to say those solar panels are not going to go on our flats because that flat is not ours and regarding cycling… I live in […] which is an hour by bike, I am not going to do that. Then I have to look for another job… ‘very nice but not very realistic’.’’* | 1.1 |
| GP_3_ | *‘’It’s a topic we’re working on and which I’ve heard a few things about from the networking days.’’* | 1.1 |
| GP_A1_ | *‘’Non-propellant driven inhalers. That’s the first and then I believe in reusable or at least refillable inhalers where possible…furthermore I just don’t know that much about it actually.’’* | 1.1 |
| CP_2_ | *‘’To what extent is the environmental impact of those pMDIs?’’* | 1.1 |
| CP_3_ | *‘’I once gave a presentation about being more sustainable in the pharmacy. I remember that switching to a green inhaler from pMDI to DPI gives CO_2_ savings per year of 90 to 200 kg per patient…the carbon footprint that you give is reduced. That’s per patient but I can’t remember the total number.’’* | 1.1 |
| CP_6_ | *‘’But that [pMDI] is more harmful to the environment because the SMI has a lower carbon footprint? Do I understand that correctly?’’* | 1.1 |
| CP_7_ | *‘’Since two or three years we have switched to digital prescriptions and I must say we already made the switch to bicycle deliveries. In addition we have institutional pharmacists who are examining how they can tackle waste because they know the conditions of medicines that remain in those constitutions so then you can more easily, just like in hospitals, examine how you can reuse something, especially expensive medicines but I’m not involved in that myself. The institutional pharmacist does that.’’* | 1.1 |
| CP_7_ | *‘’We have started working digitally as much as possible so we have much less paper use now. Recently, we also switched to bicycle deliveries instead of by car. Not necessarily for sustainability solely, also in terms of accessibility and so on… all those aspects contribute to that I believe.’’* | 1.1 |
| CP_7_ | *‘’I immediately think of DPIs because I know that those propellants contribute to CO_2_ emissions, so that’s the first thing that comes to mind’’* | 1.1 |
| CP_8_ | *‘’Lately, I’ve heard about it from different angles…CO_2_ emissions from inhalers, so I’ve noticed an increased attention. That’s good!’’’’* | 1.1 |
| P_A1_ | *‘’I recently completed a survey on inhalers and now I understand that what I use is the least environmentally friendly, actually the worst’* | 1.1 |
| P_A1_ | *‘’I’m very curious about the differences between a DPI and pMDI for the environment’’* | 1.1 |
| P_A2_ | *‘’I can imagine that those pMDIs need to be under some sort of pressure, so I can imagine that it’s less good for the environment but that’s all and it provides more waste than if you use DPIs?...something like that’’* | 1.1 |
| P_A3_ | *‘’Very exciting, climate-friendly! But I have no idea what you meant’’* | 1.1 |
| P_A4_ | *‘’I’ve never really thought about it, can you imagine, not even in my work, I have not been concerned about it at all…well it is important but I wasn’t really aware, let’s put it that way. I believe a lot of people aren’t aware of it at all.’’* | 1.1 |
| P_A4_ | *‘’I actually didn’t think much of it, now that we’re addressing it and more is known, environment and climate are of course in the news a lot, that’s when you start to think about it more.’’* | 1.1 |
| GP_A1_ | *‘’I still hear some complaints about that [waste]. More often people say ‘Well, I think it’s such a waste, I have to throw it [inhaler] away afterwards’’’* | 1.2 |
| GP_A1_ | *‘’With a DPI that plastic device is thrown away every month while a pMDI often lasts for two or three months, so I’m curious about that too.’’* | 1.2 |
| GP_A2_ | *‘’Does sustainability solely include CO_2_ emissions or does it also contain other aspects? That the device itself is taken into account, how it is made…’’* | 1.2 |
| GP_A2_ | *‘’It’s only for a month, every month you have to throw those [inhalers] away’’* | 1.2 |
| CP_4_ | *‘’I actually had a comment about those environmental considerations. You are really focused on the pMDIs now but in terms of the environment there’s a lot of factors play a role. If you think about all those devices that are thrown away every time and people get a new one every time.’’* | 1.2 |
| CP_7_ | *‘’I recently had a patient who said to receive a device every time…30 capsules plus a new device. So it’s a DPI but the packaging, it’s also plastic of course, you get that every time, that does not contribute to sustainability either.’’* | 1.2 |
| HP_2_ | *‘’Sustainability has a number of aspects for me…the entire process from development to destruction and that includes use’’* | 1.2 |
| HP_2_ | *‘’Usage itself can also be polluting e.g. think of chemotherapy in which the urine of patients is contaminated but with inhalers you also have contamination from the propellants but also from the plastic footprint that you have afterwards.’’* | 1.2 |
| HP_2_ | *‘’That’s also production’’* | 1.2 |
| P_C1_ | *‘’…it’s all plastic…you might have thoughts about that’’* | 1.2 |
| P_C2_ | *‘’A disc [DPI] is of course entirely made of plastic…and there are 60 puffs in it which lasts a month and then you receive a new one’’* | 1.2 |
| P_A3_ | *‘’It’s about two components:…is there gas in it or not and the outside which also contains plastic’’* | 1.2 |
| P_A3_ | *‘’I’m surprised when I order rotacaps…first it’s in plastic, then aluminum, that’s in a box and then another box’’I believe that can be done differently…’’* | 1.2 |
| P_A3_ | *‘’I think a lot is produced in India, so in terms of distances that is not very convenient for transport but I think that is a very broad discussion. That applies to all products of course, you want to have short lines, climate-wise.’’* | 1.2 |
| P_A5_ | *‘’What I am surprised about with the Seebri, I believe that’s a package for a month and every month you receive a new one and then automatically a new inhaler is included while actually there is not much wrong with it after a month. So in the context of plastic I would think only put in the new capsules and not a new inhaler every month’’* | 1.2 |
| GP_2_ | *‘’This is mainly about the propellant but that is also because it has simply not been made clear how much the other aspects contribute’’* | 1.3 |
| GP_2_ | *‘’It would be useful if someone would perform good research into the benefits for the environment e.g. if you put everything side by side, so a pMDI, DPI and SMI and the number of dosages, how polluting is that. You’re not going to that yourself as a GP. These are very complicated studies.’’* | 1.3 |
| GP_3_ | *‘’pMDIs contain at least 200 dosages, if you use it properly it can last a long time while something that that contains 60 dosages you’ll need a lot more [devices]…which cannot be refilled and in turn is all waste. This makes me wonder if plastic production and oil and costs are also taken into account before we blame pMDIs for everything?’’* | 1.3 |
| GP_A1_ | *‘’Furthermore I have to be honest that I personally don’t have an overview on the costs or what is more environmentally friendly, which inhaler is better regarding that aspect. So I am curious about that, then I can also inform my patients better in that area’’* | 1.3 |
| GP_A1_ | *‘’Because I don’t know much about it yet, that [sustainability] is my last priority’’* | 1.3 |
| GP_A1_ | *‘’I believe that knowledge is very important on this aspect. If I don’t know anything about it and I have no idea what a puffer costs or how harmful it is for the environment, then I can’t do much about it. If I do have insight into that aspect or when you tell me the difference of 280 kilometers and 6 kilometers, I think, oh that’s a big difference but as long you are not aware you don’t take that into account’’* | 1.3 |
| GP_A2_ | *‘’That’s also a question for me because I understood that there are also differences between pMDIs, that one is more [environmentally] friendly than the other. So you have the feeling ‘what’s wise when you don’t have that [information]. I notice then I’m less motivated as I wonder if it’s really more environmentally friendly. So for me that would really help with the motivation I believe’’* | 1.3 |
| GP_A2_ | *‘’I would like to have more information on that so that you can properly compare those DPIs and why there are so many which are thrown away after a month, that they are not reusable.’’* | 1.3 |
| GP_A2_ | *‘’If it is indeed clear, which may also be difficult when you put them [inhalers] side by side, and there is a clear difference…then yes of course [choose sustainable options], but firstly that must be indicated more clearly.’’* | 1.3 |
| CP_1_ | *‘’No, not really [sustainable actions in practice]. That is also because there is still little knowledge on the differences between DPIs and pMDIs. A lot of DPIs are thrown away, which is all plastic, what does that contribute to the environmental burden in the end? So I believe a lack of knowledge may be the cause [hampering implementation].’’* | 1.3 |
| CP_1_ | *‘’Can you provide us with some numbers on the conversion of pMDIs to DPIs, what does that contribute to the environment on a yearly basis per patient, can you say something about that?...What environmental benefit does that yield?...and have you also included the impact of production etc.?’’* | 1.3 |
| CP_1_ | *‘’There are also emissions from the production of pMDIs and DPIs. You have to subtract those [numbers] because if the DPI has a higher environmental burden, you are converting it [pMDI] into something and perhaps are suggesting ‘everyone should drive diesel instead of petrol’ but that’s not okay is it?’’* | 1.3 |
| CP_1_ | *‘’Can you give us a kind of overview, has research been performed in which the two [inhaler types] have been separated in that sense, from production to use and disposal? What is the environmental burden and carbon footprint? The delta between them, that is what we are trying to realize here.’’* | 1.3 |
| CP_1_ | *‘’I believe you can set this in motion by making it clear what the net environmental gains are when converting a person from a pMDI to DPI. Then I would at least be more motivated. Now I keep thinking that DPIs also contribute to the environment. So how much do we gain from that? As it’s so complicated with the presence of all those other factors, I believe that is the only way you can perhaps get healthcare professionals motivated’’* | 1.3 |
| CP_5_ | *‘’…from start to finish, what would it yield if we’re to convert everyone? Then healthcare professionals would be more inclined to include sustainability which isn’t done currently, other factors weigh more as isn’t clear for us.’’* | 1.3 |
| CP_6_ | *‘’There was also an article in the PW [journal of the Royal Dutch Pharmacists Association] last year from colleagues who partly calculated it…I think there are some numbers in there.’’* | 1.3 |
| CP_8_ | *‘’How that relates to each other…I mainly know the DPIs and pMDIs but with the other inhalers I don’t really have an idea what the best choice could be’’* | 1.3 |
| HP_1_ | *‘’Do you know is there a difference between inhalers with or without a spacer?…it’s a plus… people can use it but not always’’* | 1.3 |
| HP_2_ | *‘’When you look at medication, regardless of whether it works or not, I believe it is important to include the product’s footprint from cradle-to-grave,, so the entire chain. What happening is ‘cherry picking’, that’s a disappointment.’’* | 1.3 |
| HP_2_ | *‘’That’s a typical government calculation with numbers that are not correct. I mean that’s what the government does all the time.’’* | 1.3 |
| HP_2_ | *‘’I don’t know exactly whether DPIs have a smaller footprint. I also want to include the plastic footprint which is higher for DPIs. So I don’t know exactly. A lot is said but I don’t believe a word of it. There is only one study that assesses it reasonably, from 2019, but remarks can be made on that as well.’’* | 1.3 |
| HP_2_ | *‘’I don’t agree with the assumption that a pMDI is worse. You really have to be sure of that before you start advising that in guidelines. I am very much in favor of progressive guidelines but we have to know we are advising correctly.’’* | 1.3 |
| **TDF-domain ‘’Skills’’** | | |
| GP_2_ | *‘’If they don’t understand DPIs, they don’t really understand pMDIs either.’’* | 2.1 |
| GP_3_ | *‘’The disadvantage of pMDIs is that it does not contain a dose-counter.’’* | 2.1 |
| GP_3_ | *‘’The SMI does not really get anywhere [in the lungs]. The SMI also has a lot of actions, I believe it’s a difficult device.’’* | 2.1 |
| GP_3_ | *‘’I think it’s [SMI] a very difficult device. It’s the first to have a LAMA but if I could choose, I would rather have a DPI because I believe it just has too many steps. It really has a lot of steps. If you take a device like Relvar, that is just so simple, you just click it open and put it to your mouth, but when you observe what people have to do with that [SMI] inhaler en how people inhale it, a cloud comes out, I don’t know what happens so I have to say I am in favor of DPIs.’’* | 2.1 |
| GP_3_ | *‘’The tricky part with these puffs [pMDIs] is that people really have to use a spacer, except for SMIs, so that makes it more difficult...a lot of people don’t use a spacer. I’m surprised they use the one-breath method…while then you might as well use a DPI because if people are very short of breath then the one-breath method doesn’t do much for which then they have to breathe in and out calmly a few times.’’* | 2.1 |
| GP_3_ | *‘’The danger lies in that young people will leave their spacer at home or…and you want something that can also fit in your sports bag and that you can carry around.’’* | 2.1 |
| GP_3_ | *‘’The difficulty about those pMDIs is you never know how much dosages are left. At some point people order a new one. There is the CountAir that you can place on it. I always explain that in my training courses but people have to buy it themselves, it is not reimbursed by the health insurer. I would recommend if you produce a pMDI without a counter then make sure that device [CountAir] is included but that does not happen either. That makes is complicated for which I believe a lot of pMDIs are thrown away. With a dose-counter you know how much is left and you do not know that with a pMDI so I think that’s why pMDIs are still thrown away partly full.’’* | 2.1; (8.1) |
| GP_A1_ | *‘’There are inhalers without a dose-counter. These are worthless, makes me very unhappy.’’* | 2.1 |
| GP_A1_ | *‘’It used to be less but nowadays there are more types of pMDIs from different brands that don’t have a dose-counter. I sometimes indicate to deliver a pMDI including a dose-counter but then people still receive another brand that does not contain a dose-counter. I believe that would prevent lung attacks in a great number of people because they would be using an empty inhaler without a dose-counter. I think that’s a shame.’’* | 2.1 |
| GP_A1_ | *‘’I often find SMIs as alternative technically more difficult, for which you need less inhalation force. The inhaler technique is very difficult, especially for the elderly because they have to press and inhale at exactly the right moment and that twisting is very difficult.’’* | 2.1 |
| GP_A1_ | *‘’With an SMI e.g. the Spiriva, the device has to be twisted around. That’s very heavy, you really require strength in your hands in order to turn it. A DPI often only requires the opening of the valve and that’s it. With an SMI you have to start inhaling and then press it. So while you’re inhaling, you have to press so that the puff [medicine] comes out. That has to happen at exactly the right moment while with a DPI the moment you put it into your mouth you start inhaling and that’s when the powder comes out. It’s breath-controlled so to speak and with an SMI people press too fast and half of the gas is already gone before they’ve inhaled. The idea is nice, but not very feasible in practice.’’* | 2.1 |
| GP_A1_ | *‘’The SMI for example, it really has complicated actions.’’* | 2.1 |
| GP_A1_ | *‘’That can have an effect occasionally. Sometimes when I think the hygiene is not very good, we might be better off choosing a DPI than a spacer which you know will never be cleaned.’’* | 2.1 |
| GP_A1_ | *‘’The inhalation force is the biggest factor, so if patients are capable, do they have enough strength to inhale something and also to inhale deeply enough. Then there is the factor that I have to estimate whether people can use the correct technique e.g. inhaling a DPI is technically more difficult for many people whereas less can go wrong with a pMDI and spacer in terms of inhalation technique.’’* | 2.1 |
| GP_A1_ | *‘’If you provide a Ventolin in discus and you’re not able to inhale well enough, a pMDI can be a better option because the inhalation technique is easier…but then you are focusing more on the way of inhaling rather than the device itself.’’* | 2.2 |
| GP_A2_ | *‘’People don’t keep track of it [number of dosages] or they start to doubt and throw it away early. They think it must be empty or there’s still enough in it because it still makes a sound.’’* | 2.1 |
| GP_A2_ | *‘’There are a lot of lung specialists who still prescribe it [SMI] but in many cases this does go wrong. During instructions all of the steam passes before even reaching the mouth.’’* | 2.1 |
| GP_A2_ | *‘’Easy to carry around, that’s often why they [patients]don’t want a pMDI as they believe the spacer is too big. It’s a lot smaller than it used to be…but they do think that’s a disadvantage.’’* | 2.1 |
| GP_A2_ | *‘’To carry it around, that’s the most important thing. If you inhale it in the morning or evening, then of course you don’t have to carry it around but people still prefer the smallest device.’’* | 2.1 |
| CP_1_ | *‘’…in that case [SMI] the hand-lung coordination is even more important than with the pMDI’’* | 2.1 |
| CP_2_ | *‘’Ease of use, if patients can handle it well they will also use it sooner. Ease of use applies to the whole situation including placing it on the spacer. If you are at home a lot that’s not such a problem but if you travel a lot it is a problem. It is easier to carry around a small device when traveling than a spacer’’* | 2.1 |
| CP_3_ | *‘’I don’t believe a DPI is useful for someone who cannot inhale powerful enough.’’* | 2.1 |
| CP_3_ | *‘’People often travel or want an inhaler at multiple locations. Then it’s about the compactness of the device. So a pMDI without a spacer is always very popular in people, more convenient.’’* | 2.1 |
| CP_3_ | *‘’Some people just don’t have the coordination, that’s also a characteristic that can influence the choice [of inhaler] and how powerful someone can breathe.’’* | 2.1 |
| CP_4_ | *‘’The Respimat contains refill cartridges, but we also discussed that it’s not always easy for patients to click it into place. The Novolizer can also be refilled but a discus is thrown away. A lot of plastic ends up in the environment again.’’* | 2.1 |
| CP_6_ | *‘’The hand-lung coordination is actually less important in a pMDI with spacer because when you press, it [medicine aerosol] goes into the spacer. If the patient does not start inhaling immediately that’s not an issue as it is already in the spacer and will be inhaled in the next 5-10 breaths.’’* | 2.1 |
| CP_6_ | *‘’Personally I am not at all charmed by that [SMI] because it is quite complex in the number of actions, certainly in older COPD patients. A few patients come to the pharmacy every time because they cannot get it into the holder. It needs quite a bit of force and then you have to twist it’’* | 2.1 |
| CP_6_ | *‘’GSK launched the Ellipta as a successor for the Ventolin discus, that only has to be opened and it’s ready, you do not have to shake it or press it, you only have to hold it in the right position, that’s not very complex. With the pMDI the cap has to come off first and it has to go into something [spacer] and it has to be shaken. If you have a new dosage, it has to be shaken again. This contains a number of steps, which is the case with many new inhalers that have recently reached the market, while compared to a pMDI the number of steps [of DPIs] is actually very limited but if you do not have the strength…’’* | 2.1 |
| CP_6_ | *‘’The dose-counter [factor in inhaler-decision making] because they [patients] are not sure how much [dosages] is left, that’s also a reason for me to choose something else at times…and if it’s available.’’* | 2.1; 8.1 |
| CP_7_ | *‘’That [SMI] is really hard to press. Sometimes I can’t even do it myself.’’* | 2.1 |
| CP_7_ | *‘’I’ve never had anyone say that they had difficulty with the size of their Vortex [spacer].’’* | 2.1 |
| CP_8_ | *‘’With the Spiriva Respimat you have to press it in very firmly and patients often have trouble with that. When it’s delivered, they come back because we have to put it together…I often see that.’’* | 2.1 |
| CP_8_ | *‘’Sometimes patients come too early, then they believe it is already empty but it is not empty yet while they use it properly, twice per day for example. Then they should go on for another month but there is not always a dose-counter on it…then they do not keep track of it properly, the number of dosages…Nowadays you have those accessories that you can place a dose-counter on it but we do not use them actively. I believe the patient has to pay for those.’’* | 2.1; (8.1) |
| CP_8_ | *‘’There are, especially with the DPIs, standard build-in dose-counters. That’s not often the case with pMDIs I believe.’’* | 2.1 |
| CP_8_ | *‘’I especially observe it in children who are still in school, primary and secondary school, they do indeed find a spacer annoying. Instead of a pMDI they receive a Novolizer. The salbutamol Novolizer which is indeed more compact so that you can inhale quickly.’’* | 2.1 |
| CP_8_ | *‘’I can’t really think of elderly people that have trouble with the size of their device. They often have been using their device for years for their asthma and COPD. I think they are more used to it being something big. For a child it is often quite new.’’* | 2.1 |
| HP_1_ | *‘’They [SMI] are not that difficult, they can be inhaled without great force. They are good for those patients who cannot inhale with force.’’* | 2.1 |
| HP_2_ | *‘’This also depends on whether someone uses it only when necessary or permanently. Though, when necessary I sometimes also choose a pMDI with spacer in permanent surroundings/settings to ensure that it is used properly. For on the road I then choose a DPI because especially people who are active, carrying a spacer around can be annoying, especially young people do not like that. So I also consider the patient-related factors.’’* | 2.1 |
| HP_2_ | *‘’Can someone assemble a device, e.g. a SMI, assembling the Respimat is difficult for which patients visit the pharmacy regularly, they can find that very annoying. So those factors certainly play a role.’’* | 2.1 |
| P_C1_ | *‘’I find the technique for inhaling [DPI] simple and effective.’’* | 2.1 |
| P_C2_ | *‘’…using a discuss is really easy, you can carry it around everywhere you go, but you don’t have to forget it of course.’’* | 2.1 |
| P_C2_ | *‘’I have the discus, I inhale and it’s done. I only have one action and then I inhale the medicine, it’s really simple. I open it and I inhale, I turn it and put it away again. I hardly have to do anything.’’* | 2.1 |
| P_C2_ | *‘’I only use the discus and it’s very simple, you open it, you inhale, you close it again and you put it aside, that’s handy.’’* | 2.1 |
| P_A1_ | *‘’It’s quite big [spacer] if you’re going for a walk or something. So I always have a big bag with me to put it in…I got used to that at some point…I don’t care about people around me or anything like that anymore. I really use it. Even when I go for a walk, you really have to sit down if you need it.’’* | 2.1 |
| P_A1_ | *‘’I’ve had one too in the past, there was no dose-indicator on the Foster, I had to tick it off myself to see how often I had used it, that doesn’t work for me in practice. They also said you can weigh it. Well, that doesn’t work me in practice either. Sometimes I wonder if there’s anything left in it, so you either throw it away too early while there is still some left or your inhaling while there’s nothing left. That’s an issue’’* | 2.1 |
| P_A1_ | *‘’There’re ways of inhaling in which it reaches the lungs better. I notice with all those devices that go directly on to the mouth or with the spacer in front of it, that with the spacer it reaches my lungs better.’’* | 2.1 |
| P_A2_ | *‘’In that respect, every form of DPI I’ve had was easier, they’re more compact and you can carry it around’’* | 2.1 |
| P_A2_ | *‘’It [SMI] is compact regarding size by the way.’’* | 2.1 |
| P_A2_ | *‘’I agree that the discus is the easiest, I also believe that.’’* | 2.1 |
| P_A3_ | *‘’It [spacer] is not so difficult to use. If I’m going away for two days, I don’t carry it with me, then I just inhale it [pMDI] solely…that works too. Yet, the spacer does have a purpose to get receive it more precisely. Well, you don’t have that for two days, I don’t think the world will end, I don’t believe that’s a problem.’’* | 2.1 |
| P_A3_ | *‘’The Spiriva has the disadvantage that it has to be twisted/turned, it has a spring which is incredibly unhandy. I use it because it works but it’s unhandy, it’s not a button after which it [medicine] comes out, a button and then you breath in. It’s a turning spring that shoots back and when you have to place a new capsule into the device, you have to push really hard and then it often gets stuck.’’* | 2.1 |
| P_A3_ | *‘’It’s not that I don’t take my medication, I just don’t carry the spacer around.’’* | 2.1 |
| P_A4_ | *‘’Concerning the spacer, it’s a big thing to carry around when going on holiday and you have a really nice toilet bag, it doesn’t fit in there. That’s not so bad but it’s inconvenient.’’* | 2.1 |
| P_A4_ | *‘’Alvesco [pMDI] itself is actually very easy because you press it and then you inhale but that’s not even that simple because you have to do it at the same time, so you press it and then you have to inhale, that’s a coordination that requires some attention.’’* | 2.1 |
| P_A5_ | *‘’I believe they’re quite big, if you want to carry it around, you have to think about how to that neatly’’* | 2.1 |
| P_A5_ | *‘’I clean the spacer every day in a bowl of soapy water and then I let it air dry but despite doing that it still looks dirty after a few times. It’s as if I’m not taking good care of it but I am and that also makes me wonder is that rubbish also going into my lungs, what do my lungs look like on the inside?’’* | 2.1 |
| P_A5_ | *‘’I do find those inhalers very noticeable, it’s not that I’m not taking my medication discreetly, if you’re in a room where people don’t know I have asthma or what that is, I often get frightened reactions like ‘oh, are you okay?’ while ‘yes, I’m okay and to keep it that way I’m taking my medication’ so I do find that difficult about those things [inhalers], also because they have bright colors which in itself doesn’t have to be bad but it could have been less striking.’’* | 2.1 |
| P_A5_ | *‘’I have experience with the Respimat I believe, what a tornado…what a force to fire off and inhale.’’* | 2.1 |
| P_A5_ | *‘’I can’t do without [spacer], it’s [disease] often too bad for that so I have to make do with a spacer otherwise it [medication] does not come in properly.’’* | 2.1 |
| GP_2_ | *‘’Very briefly, very schematically and then they return to the GP_A_…and if they use it for the first time, they receive an inhalation instruction from the pharmacist.’’* | 2.2 |
| GP_3_ | *‘’The nice thing about a Novolizer is that once it is inhaled properly, the color changes.’’* | 2.2 |
| GP_A1_ | *‘’The standard is annually [check-up]. That’s only for people who are very stable, they are called in annually and then it depends on what is needed.’’* | 2.2 |
| GP_A1_ | *‘’With a good instruction all inhalers can be explained well and nowadays there are very good websites, videos and forms to print out. I believe you can instruct all inhalers to all patients.’’* | 2.2 |
| GP_A1_ | *‘’There are still some people of which I’m a 100% sure that I have explained it [inhaler use] to, and then they say ‘oh, I’ve never done it that way before’, that really surprises me, then I think ‘oh okay, well, that’s why it doesn’t work, right’.’’* | 2.2 |
| GP_A1_ | *‘’Or it’s long past its expiration date. I always find that so special that no one looks at that e.g. two years past its expiration date.’’* | 2.2 |
| GP_A1_ | *‘’’Those puffs [pMDIs] don’t help at all and I do it every day. I put it on the chimney and then I turn it on and it sprays into the air.’ He literally placed the puffer in the middle of the room and thought I’ll breathe that in or so. I really provided the instructions but it didn’t come through.’’* | 2.2 |
| GP_A2_ | *‘’When the diagnosis has just been made, we need to provide more support…Starting medication and learning how to deal with their disease is important. I believe self-management is very important and after that it’s often once every six months or once a year when people are stable.’’* | 2.2 |
| GP_A2_ | *‘’It’s very important to ask why they [patients] use something or not use anything and how often that is. A lot goes wrong there so it remains important to ask and gain insight into why it does not work or why people don’t do it. That’s the most important part.’’* | 2.2 |
| GP_A2_ | *‘’It also often happens that the inhaler has been empty for some time while people still keep on inhaling, that always amazes me. The display says zero, but they still keep on inhaling.’’* | 2.2 |
| GP_A2_ | *‘’You have to ask what the problem is. Is it their own stubbornness? Are they afraid? That remains the most important part.’’* | 2.2 |
| CP_1_ | *‘’The lung care is not very well organized, especially after corona, no spirometer has been measured anymore. So that’s a very undeveloped area. GP_A_ often do not know that a patient is new in the practice because it is not passed on, so that’s not going very well.’’* | 2.2 |
| CP_5_ | *‘’Lung care has also been neglected here, especially after corona. From the pharmacy, but also from GP_(A)_s, less attention has been paid while we know that adherence and use of inhaler medication often goes wrong or certainly requires attention.’’* | 2.2 |
| CP_6_ | *‘’You need to be cognitively skilled to go through all the inhalation steps properly and it’s also important for therapy adherence that people gain awareness on when to use their medication, what it’s for, that they understand when an inhaler is empty without a dose-counter. Certain capacities are required.’’* | 2.2 |
| CP_7_ | *‘’Sometimes patients specifically indicate that they find it difficult to use a spacer or their inhaler. That’s when I consciously look to see how we can make it easier. There are certain aids which can be placed on the spacer or another inhaler may be easier but I have to admit that I’m not consciously involved in converting patients from pMDI to DPI or vice versa, I don’t do that.’’* | 2.2 |
| CP_7_ | *‘’We’re not actively detecting who is therapy adherent but if it’s noticeable we will consult the patient and ask out their reasons, how they use it and what problems they encounter. If they have certain problems, we will consult the doctor about a possible switch to another inhaler.’’* | 2.2 |
| CP_7_ | *‘’I always start by consulting the patient, defining the problem, how they are using their inhaler and if I observe that it’s used incorrectly I will provide another inhalation instruction but if it’s used correctly I will talk to the prescriber to discuss if the dosage or medication needs adjustments.’’* | 2.2 |
| HP_1_ | *‘’Check over time. The first time they [patients] say ‘okay, I understand’ and then you notice at the next check it’s not so good.’’* | 2.2 |
| HP_2_ | *‘’I believe it’s [inhaler switch] possible for some, but it requires adequate guidance’’* | 2.2; (5.1) |
| P_C1_ | *‘’With the explained technique, it works sufficient for me. So far, I’ve not experienced it getting worse.’’* | 2.2 |
| P_C1_ | *‘’My pharmacy has an annual meeting and they review all the medications I use and ask ‘do you have problems with that?’ I like the fact that they revise that together annually, I appreciate that.’’* | 2.2 |
| P_C1_ | *‘’I haven’t really had any feedback about it, also because I haven’t indicated any complaints. I don’t know when my GP will start talking about it again. I do still regularly go to the GP_A_ for blood pressure and such but those inhalers are not really discussed there.’’* | 2.2 |
| P_C2_ | *‘’I also wonder, I’m inhaling every time, but am I doing it correctly?’’* | 2.2 |
| P_C2_ | *‘’What I’ve heard about the effects on voice was also mentioned in the instruction leaflet, that you have to rinse your mouth with water every time…otherwise you quickly suffer from fungal infections in your mouth…that’s why I do it very consistently. So voice related, it doesn’t have much of an effect on me.’’* | 2.2 |
| P_C2_ | *‘’Never [annual inhalation instruction/check-up]’’* | 2.2 |
| P_C2_ | *‘’I don’t need the instruction because it’s so simple that no further instructions are needed.’’* | 2.2 |
| P_A1_ | *‘’That it should regularly be checked-up on whether you are using it correctly and whether it is indeed the most suitable [inhaler] for individual patients…because everyone is different, also in terms of use.’’* | 2.2 |
| P_A1_ | *‘’In theory, an annual check-up takes place at the pharmacy but I have to admit that I’ve neglected it slightly. I believe it’s going well and the lung nurse also checks, provides comments & constructive criticism.’’* | 2.2 |
| P_A1_ | *‘’I believe I was not fully informed on the technique [DPI] at the time.’’* | 2.2 |
| P_A1_ | *‘’I currently no longer experience inhaler issues [pMDI] anymore due to very intensive instructions with this one. Really a lot. Sometimes I didn’t do something correctly but I’ve had really intensive instructions, so that makes a difference’’* | 2.2 |
| P_A1_ | *‘’That you can also inhale properly in tense situations but I think that also depends on a good instruction and also a repetitive instruction. So indeed, that you go the pharmacy annually or somewhere else for a check-up.’’* | 2.2 |
| P_A1_ | *‘’At some point, a habit can creep in and you may not be doing correctly anymore. I believe it’s important that someone regularly checks-in.’’* | 2.2 |
| P_A1_ | *‘’Actually you should gargle three times and drink something in between, that helps. I notice that it does help, my mouth is less dry afterwards.’’* | 2.2 |
| P_A1_ | *‘’If you don’t do it correctly, you will not get it in correctly. So indeed repeated instructions so that you can get it in correctly.’’* | 2.2 |
| P_A1_ | *‘’You will have to train it really well. That it [new inhaler] is something I can inhale correctly.’’* | 2.2 |
| P_A1_ | *‘’It’s also my own responsibility that I should make an annual appointment at the pharmacy and then I do receive feedback.’’* | 2.2 |
| P_A2_ | *‘’I never know for sure if I’m using it correctly and depending on how severe your asthma is, it can take a day or 2-4 before you realize that you’ve not taken your medication correctly. I’m very unhandy and I’ve coordination issues and I’m in a hurry so that does go wrong sometimes. There is no feedback telling me I’m doing it incorrect so we can say it’s easy but maybe I’ve been doing it wrong my whole life. You don’t know until you start to suffer from it but then you’re really doing it incorrectly.’’* | 2.2 |
| P_A2_ | *‘’No, never, it was explained properly once and that’s it’’* | 2.2 |
| P_A3_ | *‘’Some inhalation methods have been applied for years without realizing if you’re doing it correct’’* | 2.2 |
| P_A3_ | *‘’…maybe that [side-effects] also has to do with how it’s been used and that’s why some of it gets stuck in your throat, which is a disadvantage of dry substances by the way’’* | 2.2 |
| P_A3_ | *‘’I notice now that this [inhaler] helps, I don’t gargle anymore while I actually should do it.’’* | 2.2 |
| P_A4_ | *‘’You have to hold it in a certain way and inhale shortly after, that’s a certain skill you have to develop and at some point you get the hang of it.’’* | 2.2 |
| P_A4_ | *‘’The GP is busy enough as it is and will certainly not call me in, this also counts for the hospital…’’* | 2.2 |
| P_A4_ | *‘’I would be in favor of a check-up every now and then from either the GP_A_ or pharmacy to check if everything is going well. I know from my own experience in the pharmacy that this was often not the case and habits creep in, not doing something or not doing it correctly and that’s difficult to get rid of. For myself I believe it’s fine, I can manage, but there are a lot of people for whom it would be good to check more often whether everything is going well and whether they can still do it. E.g. the elderly, who are becoming less mobile and so on. Certainly people who’ve been using it for a longer period of time…or people with rheumatism or something. They should receive extra attention I believe.’’* | 2.2 |
| **TDF-domain ‘’Memory, attention and decision process’’** | | |
| GP_1_ | *‘’The ability to inhale, so not the age but what inhalation force someone has in order to use a DPI’’* | 3.1 |
| GP_1_ | *‘’This is also the case in children for which spacers [and pMDIs] are required.’’* | 3.1 |
| GP_1_ | *‘’So far I believe very few [sustainable actions] but it has only recently become a hot topic, so changes can be made in that aspect.’’* | 3.1 |
| GP_1_ | *‘’This is something that has only recently been in the picture. It’s not that old.’’* | 3.1 |
| GP_1_ | *‘’No, not that many [patients asking about the environmental-impact of inhalers]’’* | 3.1 |
| GP_2_ | *‘’For very young children, under the age of six or eight, I always choose a pMDI instead of a DPI and at some point also above a certain age. That also depends on whether a patient is handy, has good strength in his/her hands, that sort of aspects. So at 70 or 75 years old this could change again, shifting back from a DPI to pMDI.’’* | 3.1 |
| GP_2_ | *‘’I do know that we’ve been busy in our care group to get rid of those pMDIs. We chose, I believe, above 10 years and under 70 or 75 years and started to convert those people. We also consciously explained that to patients that it’s because of the environment.’’* | 3.1 |
| GP_2_ | *‘’Never, no [patients asking about the environmental-impact of inhalers]’’* | 3.1 |
| GP_2_ | *‘’In principle, yes [DPI preference]’’* | 3.1 |
| GP_2_ | *‘’If someone is very short of breath and you are doubting whether or not to send them to the hospital, a pMDI prescription could still be desired as you expect them not to have the strength to inhale, and of course the little ones.’’* | 3.1 |
| GP_3_ | *‘’I believe we’re not very concerned about sustainability in the whim of the day.’’* | 3.1 |
| GP_3_ | *‘’I can’t say this and this is what we’ve achieved in practice because it’s not that simple. That’s actually the current situation.’’* | 3.1 |
| GP_3_ | *‘’I prefer the DPI and that also depends on age as pediatricians are very clear on this aspect; preferably no DPIs but pMDIs under the age of 10. Everyone over 10 can start with DPIs if possible.’’* | 3.1 |
| GP_3_ | *‘’I believe a pMDI is intended for those who cannot use a DPI.’’* | 3.1 |
| GP_3_ | *‘’People [healthcare professionals] often see someone [patients] when they are short of breath and then start a pMDI…in emergency or acute situations or with locums, a lot of people start with pMDIs. Only when people enter into chain care [mutli-disciplinary coordinated care] other choices are made. I believe that’s one of the reasons a lot of pMDIs are prescribed.’’* | 3.1 |
| GP_3_ | *‘’I provided a training on this topic, in principle anyone between the ages of 10 and 65 who can count to 10 and hold their breath can use a DPI.’’* | 3.1 |
| GP_3_ | *‘’Between 10 and 65 a DPI should be possible and with very elderly people I often observe that doesn’t work for them. I also had someone recently, that man was very short of breath and the SMI did not work at all, then you have to use a pMDI with mask, not rely on any technique. That man had a saturation of 75% and I wanted him to stay at home as he was terminal but he recovered completely with just a Bevespi and prednisone treatment.’’* | 3.1 |
| GP_3_ | *‘’In general, asthma patients are somewhat younger so they should be on DPIs even more since it’s easier to carry around.’’* | 3.1 |
| GP_3_ | *‘’Vulnerable elderly people where medication has to be provided by caregivers, everyone has to instruct then so that’s difficult for which a pMDI is the easiest technique. Just breathe in and out calmly five times, that can still be learned. So I believe this is also a vulnerable group.’’* | 3.1 |
| GP_3_ | *‘’In my experience not [difference in effectiveness between device types].’’* | 3.1 |
| GP_3_ | *‘’Some people find it unpleasant to taste the powder in their mouth, people may find that annoying, that’s less with pMDIs.’’* | 3.1 |
| GP_3_ | *‘’In principle that’s standard [start on DPIs] but the question is whether that’s shared decision as I don’t even give people the option of a pMDI.’’* | 3.1; 3.2 |
| GP_A1_ | *‘’In our practice, maybe once a year a patient asks what is more environmentally friendly, otherwise I never get that question from patients.’’* | 3.1 |
| GP_A1_ | *‘’No, not really [consider sustainability].’’* | 3.1 |
| GP_A1_ | *‘’For me there is no such thing as age, it is very rarely age-dependent.’’* | 3.1 |
| GP_A1_ | *‘’The initial assessment is always based on the capability of patients to use their inhaler. This depends on the hand motor skills of elderly but this may also concern children or people with rheumatism or whether they can use their inhaler correctly.’’* | 3.1 |
| GP_A1_ | *‘’A pMDI sticks less to the mouth or throat which gives fewer side-effects than most DPIs.’’* | 3.1 |
| GP_A1_ | *‘’Flixotide pMDI has larger particles than a Foster or Quvar, then a fine particle pMDI would be preferred if people suffer from fungal infections in the mouth.’’* | 3.1 |
| GP_A1_ | *‘’They have less strength but that doesn’t only apply to rheumatism, others as well. That’s observed as it’s performed together with the patient, you observe the discomfort and difficulty, it’s not a large group but it’s decisive.’’* | 3.1 |
| GP_A1_ | *‘’People of 50 years old sometimes have a poor hand function, while people of 90 years old can still do it fine. No, I don’t think age really plays a role.’’* | 3.1 |
| GP_A1_ | *‘’I do believe people with Seretide experience side-effects sooner compared to Foster which has finer particles; fungus, cold and hoarseness complaints. Sometimes I want to switch to Foster and you observe a reduction regarding those side-effects. That’s certainly not the case with everyone, but there is a considerable group of people who experience side-effects faster with Seretide.’’* | 3.1 |
| GP_A1_ | *‘’Spiriva was actually the first drug on the market that worked for 24 hours. Nowadays that’s also possible in those Ellipta’s for example so I believe it’s a habit, more old-fashioned, I don’t really understand why they would still choose for a SMI.’’* | 3.1 |
| GP_A1_ | *‘’pMDIs are all the same actually. They all have to be shaken and require a spacer. In the past, I don’t think that’s available anymore, there were capsules that would go into a DPI. That was very unfriendly for the patient, then they [patients had to open the strips, that wouldn’t go well or the capsules didn’t puncture open properly. I don’t know if it still exists but I do not prescribe it anymore.’’* | 3.1 |
| GP_A1_ | *‘’The cheaper brands often don’t have a dose-counter, that’s the biggest problem for me.’’* | 3.1 |
| GP_A1_ | *‘’The size of the dose-counter can be a problem for many people. If they are visually impaired and it contains a tiny counter, it’s useless. Then you’re more likely to choose an Ellipta which does contain a large counter.’’* | 3.1 |
| GP_A2_ | *‘’No, [take sustainability into account]’’* | 3.1 |
| GP_A2_ | *‘’Some people encounter more side-effects with a DPI, whom are then put on a pMDI. Children from 0-6 are always put on pMDIs.’’* | 3.1 |
| GP_A2_ | *‘’There is only a small group that has too little strength but that’s really a very small group and that are often the elderly.’’* | 3.1 |
| CP_1_ | *‘’Because of the preference policy in which the health insurer determines at substance level which product they will reimburse, pMDIs are more interchangeable. Whether it comes from manufacturer A, B or C, it’s put on to the spacer and does the same. With the DPI there’s a proliferation of all kinds of different devices which also contain quite a few which are of poor quality. Therefore, in some cases, DPIs are deliberately not chosen because the device does not meet the quality requirements. I believe as a consequence there may have been an increase towards pMDIs since the preference policy.’’* | 3.1; 8.1 |
| CP_4_ | *‘’It’s not necessarily about the difference between asthma and COPD but more about the kind of patient that’s in front of you. Based on the patient, you look at their situation…in my opinion it’s on a patient-level. Of course you can generalize slightly, but in the end it’s about the individual patient.’’* | 3.1 |
| CP_6_ | *‘’…my region has traditionally been very DPI oriented…’’* | 3.1 |
| CP_6_ | *‘’If I consider what I’ve done in the past 10 years, I have provided asthma patients with DPIs more often than COPD patients. I notice that I’m shifting now…but I’ve done this in the past years.’’* | 3.1 |
| CP_6_ | *‘’If someone is cognitively strong and has enough strength, a DPI is first choice.’’* | 3.1 |
| CP_6_ | *‘’I mainly choose pMDIs if people have cognitive problems e.g. when home care plays a role. You can inhale that and reach sufficient deposition, even if you can’t breathe consciously.’’* | 3.1 |
| CP_6_ | *‘’That largely depends on the age category. People who are on the road are often the younger patients, that often contains an asthma component. Then you look at what’s easy to carry around, not a spacer but a DPI or a breath actuated pMDI, something like that. COPD patients are often more housebound, so choosing a spacer is more easily made.’’* | 3.1 |
| CP_6_ | *‘’With asthma I look at a younger category, how can I fit it into their life. With COPD I focus more on the cognition…you also talk about ease of use but it’s more about their capability, can people use their inhaler, that’s often the older category who are often sicker, especially in the later stages of COPD.’’* | 3.1 |
| CP_6_ | *‘’Instinctively, I tend to choose DPIs faster for asthma patients as I have a younger person in front of me but I have to say that lately I’m shifting more towards DPIs in COPD patients. That’s because I had to delve into the different resistances [of DPIs] for a training course. Nowadays you also have DPIs with a low resistance that contain a once daily dose. This is also very useful for COPD, so this is shifting somewhat.’’* | 1.1; 3.1; 5.1 |
| CP_7_ | *‘’We have quite an old population here in this neighborhood. I’ve also had people on DPIs who did not have the strength to open the valve. The manufacturer produces the inhaler in such a way that you have to open or close the valve with force, older people without strength suffer from that.’’* | 3.1 |
| CP_7_ | *‘’I also observe that pMDIs and spacers are still regularly prescribed’’* | 3.1 |
| CP_7_ | *‘’I believe inhaling [pMDI] is easier for the elderly because they don’t have to hold their breath.’’* | 3.1 |
| CP_7/8_ | *‘’No [environmental impact is not included in inhaler choice]’’* | 3.1 |
| HP_1_ | *‘’I don’t have a specific reason but I believe the DPI is somewhat more effective.’’* | 3.1 |
| HP_1_ | *‘’In practice, I prefer the DPI.’’* | 3.1 |
| HP_1_ | *‘’No, honestly, I don’t do that [discuss environmental impact of inhalers with patients].’’* | 3.1 |
| HP_2_ | *‘’I have the idea that a pMDI, if used properly with a spacer, is more effective.’’* | 3.1 |
| HP_2_ | *‘’I also consider side-effects because DPIs have more side-effects than pMDIs when used with a spacer, regardless of molecules.’’* | 3.1 |
| HP_2_ | *‘’The Netherlands used to be a DPI country…In principle [prescribe] DPIs if patients are capable and meet all criteria.’’* | 3.1 |
| HP_2_ | *‘’If there is a specific problem, I would rather choose a pMDI because the patient is central in this.’’* | 3.1 |
| HP_2_ | *‘’Not really [differences between diseases] regarding device type. You consider if the patient can consciously breath and is able to hold it well, exhale etc. These are determining factors, the hand-mouth coordination is also a factor and the inhalation force. Those are the three factors. It’s possible that a patient with COPD can do all three aspects and a patient with asthma cannot. The reverse is also possible, so it’s not disease-specific. Diseases do play a role, from co-morbidity to strength, but it’s one overall group.’’* | 3.1 |
| P_C1_ | *‘’Has not been addressed [environmentally friendly options discussed with healthcare professional]’’* | 3.1 |
| P_A1_ | *‘’No! [environmentally friendly options discussed with healthcare professional]’’* | 3.1 |
| P_A2_ | *‘’Only if you’re very short of breath or having a hard time, a DPI will not work. At least not for me.’’* | 3.1 |
| P_A3_ | *‘’Salbutamol occasionally, those are rotacaps with capsules where those pins go in and always break.’’* | 3.1 |
| P_A3_ | *‘’I’ve had it [DPI discus] in the past, I found it easy. I also have salbutamol, rotacaps with those pins. That’s really inconvenient. Now that I hear this, maybe I’ll go back to the discus.’’* | 3.1 |
| GP_1_ | *‘’If I say to the patient: we’re not going to take an X-ray of this knee because I know the result and it’s not good for the environment. I don’t believe the patient is happy with that but I do believe it’s different with pMDIs. I think you could convince a patient in this case,‘this is really bad for the environment’, because we know that people have already made changes regarding propellants in aerosol cans.’’* | 3.2 |
| GP_3_ | *‘’I usually provide a choice, I have a turbuhaler, a discus and a novolizer on my desk. Something along the lines of: ‘We’re going to start medication, you can choose from these three devices.’’’* | 3.2 |
| GP_3_ | *‘’I do direct towards DPIs’’* | 3.2; 4.2 |
| GP_3_ | *‘’It depends on their knowledge and of course in two years it could all have been adjusted again but it could also be that people say ‘I like to keep it that way, I’m well-adjusted now,’ but I do think you can offer it to people.’’* | 3.2 |
| GP_3_ | *‘’You ask people for their preferences and then you offer the possibilities in which you can indicate whether you’re environmentally conscious or not…In that sense it’s shared decision making.’’* | 3.2 |
| GP_3_ | *‘’I assess all lung functions and then my advice is asked, what should we start with? I make a proposal but I don’t have a patient in front of me. So patients do not have everything to choose from. A diagnosis and proposal is made and sometimes that’s told through the phone… that’s the reality.’’* | 3.2; 4.2 |
| GP_3_ | *‘’In principle that’s standard [start with DPI], but is that shared decision making as I don’t give people the option of a pMDI?’’* | 3.1; 3.2 |
| GP_A1_ | *‘’You could also discuss it with people ‘in terms of sustainability, it’s better to use this. Would you like to try that?’ That could be possible of course.’’* | 3.2 |
| CP_7_ | *‘’We have placebo’s.’’* | 3.2 |
| CP_7_ | *‘’I believe that [sustainable decision-making] depends on the patient whether they want to cooperate, but I would certainly suggest it.’’* | 3.2 |
| CP_7_ | *‘’I believe the patient has to be willing and whether a conversion is possible or not, that’s the most important part.’’* | 3.2 |
| CP_8_ | *‘’I am open to that and I believe the patient is also open for it in general.. Maybe it’s a bit more difficult in my neighborhood but in general I believe the patient is willing. It’s a hot topic, everyone is talking about it, sustainability.’’* | 3.2 |
| P_C1_ | *‘’I must say that my GP and the other people involved have provided me with a tailored healthcare service but I may also only be a very mild COPD patient, so it could be more difficult in other cases.’’* | 3.2 |
| P_C1_ | *‘’What effects to expect of both [devices] were told and I actually agreed with them, to try it first and then see how it goes and it’s still going well. No reason to change that.’’* | 3.2; 4.2 |
| P_C2_ | *‘’I didn’t have a choice, I was prescribed the discus. I believe that if I had a choice I wouldn’t have known what to choose as I’m inexperienced. The medication is prescribed since you have a complaint but you accept what is prescribed because I wouldn’t know what is better or not. If it turns out not to be suitable then you can talk about it afterwards ‘this doesn’t work, what else do you have to offer?’.’’* | 3.2; 4.2 |
| P_A1_ | *‘’Based on the knowledge and situation in the world right now? Yes, yes! [willingness to discuss environmentally friendly options]’’* | 3.2 |
| P_A1_ | *‘’I would like to practice with a training device to evaluate if that’s something I could use…’’* | 3.2 |
| P_A1_ | *‘’That you receive good training, with a dummy device to see what’s suitable for you, as there are so many types now when I look at the desk of the lung specialist. There are 20 or 30 I believe, it’s quite a large selection and there a lot of types too. So to get a good grip on that…you are of course dependent on your doctor.’’* | 3.2; 4.2 |
| P_A2_ | *‘’It’s fine if a test is done in advance. ‘We want to shift over to a new medicine because this and this and this but we want to be sure that it suits you. This is how we test it and these are the risks, that it works, that’s fine in a safe setting, but not that you visit the pharmacy, you only have two puffs left and then if it doesn’t work…’’* | 3.2 |
| P_A2_ | *‘’I don’t ask for input in advance. If you get 5 options, just do whatever. I can’t even choose ice cream, let alone medication. It just has to work and if it doesn’t work or it’s not nice then I’ll come back.’’* | 3.2; 4.2 |
| P_A3_ | *‘’I find that very worrying as patient, I find that really worrying. Then we are ultimately guided by the market model. You don’t even have a choice, you are just presented with something new and you don’t even know whether it’s exactly the same or how the lung specialist feels about it. You hear it from the pharmacy, there is poor communication on that aspect…It’s the question whether it’s the same, you don’t know what’s in it, or whether it’s different, you have to find that out for yourself.’’* | 3.2; 8.1 |
| P_A5_ | *‘’As patient you don’t have a choice, with all due respect to the pharmacist, ‘well look ma’am I’ve your medication and the active ingredient is the same’ then you have to make do with it. I believe that’s the case but if I say I prefer my own since I know that works ‘no sorry you’ve to make do with this now’ and in three months I’ll get something new again. Then I think well good luck these next three months.’’* | 3.2; 8.1 |
| P_A5_ | *’I would like to be assured patients receive the most suitable inhaler and that this will only be deviated from in consults or with clear reasoning…That we’ve a choice, do we want that or not?’’* | 3.2 |
| GP_3_ | *‘’That it contains a good dose-counter, those aspects are important.’’* | 3.3 |
| GP_A1_ | *‘’Force of inhalation. That’s the first aspect we take into consideration and the ease of use. If it’s more user-friendly then this will also affect adherence.’’* | 3.3 |
| GP_A1_ | *‘’First the force of inhalation, ease of use and dose-counter and then the adherence because if people don’t take it accordingly, you can better provide a once daily dosing scheme.’’* | 3.3 |
| GP_A1_ | *‘’The patients’ interest always comes first, what we believe is best for the patient in all those aspects mentioned. If it doesn’t matter what kind of DPI to choose, then I would look to consider what is more sustainable, what is better for the environment?’’* | 3.3 |
| CP_2_ | *‘’Haven’t done anything [environmentally friendly actions] as I wonder…it’s already so complex to find the most suitable inhaler for an individual that sustainability has a lower priority’’* | 3.1; 3.3 |
| CP_4_ | *‘’Ease of use plays an important role, if the patient uses multiple medications, multiple inhalers. If you want to stay in the same group of inhalers and the patient uses more medication, can you get them all in one sort of device? So, that plays a role.’’* | 3.3 |
| CP_6_ | *‘’There are many factors involved. What does someone need exactly?’’* | 3.3 |
| CP_6_ | *‘’Whether I consciously include all those aspects every time? No, I don’t.’’* | 3.3 |
| HP_1_ | *‘’That depends on the specific situation, how the patient feels but also what is needed.’’* | 3.3 |
| HP_1_ | *‘’I don’t have one specific parameter that I always consider, I want to see what’s the best option first.’’* | 3.3 |
| HP_2_ | *‘’Is conscious inhalation possible, how is the technique, inhalation force and the hand-mouth coordination. Those are the three factors that determine my choice for a device in which previous experiences also play a role.’’* | 3.3; 5.1 |
| HP_2_ | *‘’In addition, the molecules, the inhalation technique, the firing rate and therapy adherence.’’* | 3.3 |
| P_C1_ | *‘’Easy to use and a fast effect. Effectiveness. Fortunately, that’s the case for me.’’* | 3.3 |
| P_C1_ | *‘’It’s [effectiveness] at the top and also number one’’ (nr 1 factor of importance – decision making)* | 3.3 |
| P_C1_ | *‘’I would say easy to use’’ (nr 2 factor of importance)* | 3.3 |
| P_C1_ | *‘’Fast effect is also important’’ (nr 3 factor of importance)* | 3.3 |
| P_C1_ | *‘’The size and compactness are important, that it’s easy to carry around.’’ (nr 4 factor of importance)* | 3.3 |
| P_C1_ | *‘’The costs, but so far everything has been covered, if it were to cost a bit more then yes, it might be a matter of finding out what the possibilities are as alternative…I’m quite cost-conscious…I do think it’s important somehow.’’ (nr 5 factor of importance)* | 3.3 |
| P_C1_ | *‘’I would say, shared sixth place [side-effects]’’ (nr 6 factor of importance)* | 3.3 |
| P_C1_ | *‘’Skills…if the instruction is not good or you cannot do it, then it could still be important to make an adjustment. Right?’’ (nr 7 factor of importance)* | 3.3 |
| P_C1_ | *‘’The frequency of inhaling…because I only have to do that once a day and the other one is whenever I feel the need…I think the sound effects and cleaning [of device] are even less important than the frequency.’’ (nr 8 factor of importance)* | 3.3 |
| P_C1_ | *‘’Did I mention the dose-counter yet?...I think it’s handy that it’s there.’’ (nr 9 factor of importance)* | 3.3 |
| P_C1_ | *‘’I don’t have a spacer so that would be number 10, I think, I don’t need it yet.’’ (nr 10 factor of importance)* | 3.3 |
| P_C1_ | *‘’Around factor 3 I believe [place of sustainability], I think it’s important especially after what was just said.’’* | 3.3 |
| P_C1_ | *‘’I’m positive about taking that step [switch] but it has to remain the same for the patient’’* | 3.3 |
| P_C2_ | *‘’The effectiveness of my inhaler. It has to have a good effect…that is very important to me.’’ (nr 1 factor of importance)* | 3.3 |
| P_A1_ | *‘’Examining what suits you the best, in consults with your doctor, because I understand that there are new inhaler types these days. That they look carefully at how you use it and what is best for you.’’* | 3.3 |
| P_A1_ | *‘’I sometimes try-out if I can use my inhaler without a spacer. Well, that doesn’t work at all. Then I also have problems with inhaling, so technically I won’t be able to handle everything that well. Despite the fact that it is the least friendly for the environment, I find the spacer very pleasant to use.’’* | 3.3 |
| P_A1_ | *‘’I was actually advised not to start with that [DPI] at all, because of my lung function, I can also not inhale properly technical-wise, so that was said immediately ‘you can better keep going on with what your using now, the spacer’. That is already a piece of tailored care for me.’’* | 3.3 |
| P_A1_ | *‘’That it works fast…I believe the Trimbow takes 10 to 15 minutes before it really works. I believe that’s important, especially if your short of breath somewhere, that it works quickly.’’* | 3.3 |
| P_A1_ | *‘’Effectiveness’’ (nr 1 factor of importance)* | 3.3 |
| P_A1_ | *‘’Yes, I agree [ease of use].’’ (nr 2 factor of importance)* | 3.3 |
| P_A1_ | *‘’Number of actions/steps’’ (nr 3 factor of importance)* | 3.3 |
| P_A1_ | *‘’Fast effect’’ (nr 4 factor of importance)* | 3.3 |
| P_A1_ | *‘’…the dose-counter’’ (nr 5 factor of importance)* | 3.3 |
| P_A1_ | ‘*’The side-effects can be severe. I’ve had some pretty severe side-effects, even though I don’t exactly remember what they were. That was a reason to change [inhaler] again.’’ (nr 6 factor of importance)* | 3.3 |
| P_A1_ | *‘’The spacer’’ (nr 7 factor of importance)* | 3.3 |
| P_A1_ | *‘’Yes, the skills’’ (nr 8 factor of importance)* | 3.3 |
| P_A1_ | *‘’The sound effects because if you are somewhere where it’s quiet, you cannot use it. It has never happened but imagine that you are at a concert, then you would not be able to use it, then you would really have to go outside, that is also possible, so for me that’s next.’’ (nr 9 factor of importance)* | 3.3 |
| P_A1_ | *‘’No, I’ve gotten used to that by now [cleaning the inhaler]. So that would be the next.’’ (nr 10 factor of importance)* | 3.3 |
| P_A1_ | *‘’The frequency [of inhaling] does not matter for me.’’* | 3.3 |
| P_A1_ | *‘’The size or compactness, that you have to carry it in your bag. You always have to take that into account.’’ (nr 11 factor of importance)* | 3.3 |
| P_A1_ | *‘’I believe at 6 [place of sustainability] because the other factors are still more important for me…it sounds very selfish…but yes, no, for me those other factors are more important.’’* | 3.3 |
| P_A1_ | *‘’With the knowledge I have now, I believe it’s very important that all of the mentioned aspects are taken into account.’’* | 3.3 |
| P_A1_ | *‘’It may sound silly, but I am happy with the use of this…I notice that it has a great effect on me in practice, so is there a comparable DPI inhaler available then?* | 3.3 |
| P_A2_ | *‘’The number of actions/steps don’t matter that much. If they are logical actions then it doesn’t matter if you have to do 4 steps or 20. If it goes smoothly that’s not a problem as far as I’m concerned. If you have 1 very difficult action then I find that worse than 10 very easy ones. As long as you don’t spend 10 minutes on preparing.’’* | 3.3 |
| P_A2_ | *‘’I really liked those powder capsules but if it didn’t work then it really did not work, those things got really stuck. That’s frustrating because you often have to do it several times per day, 1-2 times/day and it’s often at moments that you’re in a hurry, you just want to ‘click-click-click’ automatically and not have to think about it.’’* | 3.3 |
| P_A2_ | *‘’Absolutely not! [most important, sustainability] If it [alternative] works and everyone has access, it’s [sustainability] a fair third/fourth factor to consider’’* | 3.3 |
| P_A2_ | *‘’The effectiveness is absolutely the most important! By far the most important!’’ (nr 1 factor of importance)* | 3.3 |
| P_A2_ | *‘’It’s an important factor, even if you experience almost no side-effects, it’s still important that they remain to a minimum.’’ (nr 2 factor of importance)* | 3.3 |
| P_A3_ | *‘’The same for me! [importance of effectiveness] (nr 1 factor of importance)* | 3.3 |
| P_A3_ | *‘’Exactly the same! [minimum of side-effects]’’ (nr 2 factor of importance)* | 3.3 |
| P_A3_ | *‘’For me it’s [sustainability] not the most important factor.’’* | 3.3 |
| P_A4_ | *’Maybe I would like to think about switching, when I hear about the difference of pMDIs vs. DPIs, but only under the condition that it works just as well. That’s difficult because you’ll have to test it, use it for a few weeks etc. So the practical feasibility is another issue I believe.’’* | 3.3; (8.1) |
| P_A4_ | *‘’By far the most important, effectiveness! (nr 1 factor of importance)* | 3.3 |
| P_A4_ | *‘’I also place it at 2 [side-effects].’’ (nr 2 factor of importance)* | 3.3 |
| P_A5_ | *‘’…‘’I think it’s a certainty and right that you get what benefits you, that it isn’t a game of Russian roulette’’* | 3.3 |
| P_A5_ | *‘’Yes, also place 1 [effectiveness]’’ (nr 1 factor of importance)* | 3.3 |
| P_A5_ | *‘’I think at place 2 [minimum of side-effects]’’ (nr 2 factor of importance)* | 3.3 |
| P_A5_ | *‘’I think it is important that it is discreet and also somewhat mild in use’’* | 3.3 |
| **MOTIVATION** | | |
| **TDF-domain ‘’Professional role and identity’’** | | |
| GP_1_ | *‘’Very, yes! [responsible for factoring sustainability in healthcare]’’* | 4.1 |
| GP_2_ | *‘’There are so many aspects to consider as a GP, so one chooses this and the other chooses that. You can’t implement everything at the same time or you’ll go completely crazy!’’* | 4.1 |
| GP_2_ | *‘’…and the interests [barrier hampering implementation]’’* | 4.1 |
| GP_3_ | *‘’All GP_A_ are very loyal, they visit every training but I do notice that GPs are more difficult to motivate, they already have so many subjects, or maybe this subject does not interest them. That makes it more difficult as GPs often start treatment in acute asthma patients.’’* | 4.1; 4.2 ; 8.1 |
| GP_3_ | *‘’The question is to what extent this is implemented in the various practices. I provide information or education but the implementation must take place in the practice itself.’’* | 4.1 |
| CP_1_ | *‘’I do believe it would be very good to include sustainability in many aspects, there are many other aspects of medicine and the surrounding industry where sustainability plays a role.’’* | 4.1 |
| CP_8_ | *‘’I do see the importance and I do believe people would like to contribute somehow.’’* | 4.1 |
| HP_1_ | *‘’In reality this should be the case [responsibility for factoring sustainability in healthcare]…but right now, I think we’re not.’’* | 4.1 |
| HP_2_ | *‘’Yes [responsibility for factoring sustainability in healthcare]’’* | 4.1 |
| P_C2_ | *‘’I saw this research and thought it may be important to contribute to this.’’* | 4.1 |
| P_A2_ | *‘’I think it’s very important that it happens but it’s more important that it has no impact on the people who use it.’’* | 4.1; 6.1 |
| GP_1_ | *‘’Most people are seen in the acute phase if they are a starter so then you start with salbutamol. Follow-up and explanations are provided by the GP_A_. Often I send acute patients immediately to the GP_A_ to provide an explanation and otherwise the pharmacy does that. I myself never give instructions on how to use an inhaler.’’* | 4.2 |
| GP_1_ | *‘’So we’re going to focus on possibilities in the practice, also on medication use. We examined the percentage of pMDI users in our practice and if there is something we can do about that? We’ll discuss that with each other and then we’re going to make a plan for everyone who wants to join, who wants to start with it…I know that 39% of patients receive a pMDI here, we’ve a list from above 7 [years], that’s quite a large group. We can examine that specifically as not many have done anything yet.’’* | 4.2 |
| GP_2_ | *‘’In daily practice, I don’t really do that either [discuss environmental impact]. People are either very sensitive and will address it themselves or they don’t and then you will not win them over, then they themselves are just more important.’’* | 3.1; 4.2 |
| GP_2_ | *‘’I don’t see it as my job to refer every patient to that [sustainability], that’s not directly what there here for, they come for something completely different. Sometimes you can factor it in, but that’s not really what happens in my consultations yet.’’* | 3.1; 4.2 |
| GP_3_ | *‘’I believe we as GPs play a major role in this [sustainable inhaler decision-making].’’* | 4.2 |
| GP_3_ | *‘’The disadvantage of a pMDI is that it doesn’t have a dose-counter. I find it a shame that such aspects aren’t cot on to by the industry. It’s possible, a pMDI with dose-counter, that has been proven with Foster etc.’’* | 4.2; 2.1 |
| GP_3_ | *‘’I have to say, it’s not like we’ve set a really hard target for DPIs.’’* | 4.2 |
| GP_3_ | *‘’It’s a shame that the industry doesn’t fully think about what’s needed in the field, everyone is busy with their own interests…making sure there’s a range from A-Z whether that’s for Novolizer or Foster, I don’t care, just make sure that you have 1 type for all those…That’s to do with their interests, that this is not cot on to. That’s not financially interesting or the market is not big enough etc.’’* | 4.2 |
| GP_3_ | *‘’Most of the care is structured care which is predominantly provided by GP_A_s.’’* | 4.2 |
| GP_3_ | *‘’We only see very severe patients, people who keep experiencing lung attacks or who are really not doing well, who are very short of breath. Those patients visit us.’’* | 4.2 |
| CP_1_ | *‘’Many health insurers also signed the Green Deal, so they all have to move towards sustainability too…informing them on ‘what if we did this and that, we could have an enormous impact on the environment’ then perhaps that will also motivate them to start looking at the preference policy from a different perspective.’’* | 4.2 |
| CP_3_ | *‘’What also needs to be taken into account is that pharmacists don’t always have insight into what’s been discussed with the prescriber. Perhaps this aspect [sustainability] has been considered with the patient but he/she really doesn’t want to change. At the counter they’re also not always willing to talk about it.’’* | 4.2 |
| CP_3_ | *‘’Also be aware that we don’t have much say in this.’’* | 4.2 |
| CP_6_ | *‘’It [formulary] guides GPs when using their system and therefore more uniformity in inhaler prescriptions is observed in the region but that’s a process of years and that’s also the problem. Covid didn’t help either, it’s very difficult to keep those projects and processes alive, that it doesn’t fade out.’’* | 4.2 |
| CP_6_ | *‘’If you have a good relationship with your GPs and you can discuss those aspects, then I can well imagine that it’s more easier in the village I’m in than when you’re a pharmacist in the city.’’* | 4.2 |
| CP_7_ | *‘’We receive the prescriptions and we deliver them, but without specifically asking doctors or patients what the reason is for this type of inhaler.’’* | 4.2 |
| CP_7_ | *‘’When it comes to sustainability, I believe the manufacturer can also play a key role in terms of making sure that it’s made from recycled plastic or that devices have a longer life-span.’’* | 4.2 |
| CP_7_ | *‘’In practice, I only hear when things aren’t going well or when there’s a problem through the assistants. If everything is going well, I don’t hear anything.’’* | 4.2 |
| CP_8_ | *‘’I do try to respect the prescriber’s choice as he/she has made this choice with a certain idea in mind.’’* | 4.2 |
| CP_8_ | *‘’The GP_(A)_ is located in our practice. So occasionally we [pharmacy] look together with the patient what’s suitable. I have done that once, it’s not standard policy, but then I examine what would suit the patient and discuss it afterwards with the GP_A_, then he/she prescribes what’s necessary.’’* | 4.2 |
| CP_8_ | *‘’As a pharmacist you don’t know the underlying idea of GP_(A)_s. Usually, what I can tell from the type of inhaler prescribed is that if it’s a pMDI the inhalation force will probably be bad but maybe the inhalation force is good and a discus is difficult to use for the patient. We don’t know that.’’* | 4.2 |
| CP_8_ | *‘’I think it would be good to at least know how GP_(A)_s come to their decision for a particular inhaler. I know that there are pharmacists who also do spirometry themselves, a small device in the consultation room, but I believe that in order to switch to another medication or device, you have to do a spirometry or have more information from the GP_A_ because switching is difficult if you don’t know that.’’* | 4.2 |
| CP_8_ | *‘’I only hear about it when it’s really not going well, then it reaches us and you start investigating.’’* | 4.2 |
| CP_8_ | *‘’It’s good to know what everyone else is doing…maybe it’s useful for if we start switching [inhalers].’’* | 4.2 |
| CP_8_ | *‘’That healthcare professionals have examined together what is best for the patient and then you could also incorporate the sustainability aspect in terms of what’s recommended as first choice. If that is then respected by the health insurers, you’re there.’’* | 4.2 |
| HP_1_ | *‘’Personally I usually see patients, adults, and they already have inhaler therapy so it’s not the first time they see a doctor. As a specialist it’s always a second check.’’* | 4.2 |
| HP_2_ | *‘’All stakeholders need to be involved, from health insurers to government to the patient themselves…you have the strongest and weakest link.’’* | 4.2 |
| P_C1_ | *‘’It starts with the information you’re offered by you healthcare professional. The fact that it has not been discussed up to now, the environmental effects, well I’m very curious whether that will happen the next time and if he will not bring it up, I will ask about it myself following this conversation.’’* | 4.2 |
| P_C1_ | *‘’I might start asking ‘what else do we have and what are the consequences and how environmentally friendly is it?’ but that feels a bit remote to me. I assume I can continue as I’m used to now.’’* | 4.2; 3.2 |
| P_A2_ | *‘’It’s not my problem to improve. I think it’s really cool but it shouldn’t become my problem so if time and energy can be put into it and I would work in the industry, I would be totally focused on it.’’* | 4.2 |
| P_A4_ | *‘’The question is also whether you should start that [discuss environmental-impact] as an individual patient or whether it should be properly investigated first. That’s when your research came by, so I think that’s good.’’* | 4.2 |
| P_A4_ | *‘’I have to admit that until I actually read about those CO_2_ emissions and the impact of propellants, I thought ‘oh my gosh that’s intense, I have to change’ but how and when, you don’t do that so quickly.’’* | 4.1; 4.2 |
| P_A5_ | *‘’I don’t really have suggestions on how that could be implemented, the environmental impact, because it’s not my profession, so I don’t know.’’* | 4.2 |
| **TDF-domain ‘’Beliefs about capabilities’’** | | |
| GP_1_ | *‘’The InCheck-DIAL may be used’’* | 5.1 |
| GP_1_ | *‘’I think if you have a very severe asthma patient, I wouldn’t change [related to switching], but especially in people with mild or moderate asthma, I would discuss and recommend it [DPI].’’* | 5.1 |
| GP_2_ | *‘’I’ve actually switched everyone who was on a pMDI to a DPI based on their age in the past period. One or two [patients] didn’t accept that. They tried it once or twice and said ‘this doesn’t work for me’ so I placed them back on a pMDI.’’* | 5.1 |
| GP_2_ | *‘’Most people were fine with it. People who visited the GP_A_ were automatically converted when they got there and we tried to reach the rest with the buffer. Except for a few who didn’t respond well to a DPI, that actually went well.’’* | 5.1 |
| GP_2_ | *‘’I agree with GP_1_ that with some [patients] you’ve been working on it for three years to get to an acceptable level, I’m not going to change that, they often also go the lung specialist but most of the patients in the practice you can try to convert and if it doesn’t work, you can place them back.’’* | 5.1 |
| GP_2_ | *‘’We’ve taken out the young and old of course. I believe you should be able to capture approximately 60-70%t or 60-65%.’’* | 5.1 |
| GP_3_ | *‘’That can be checked with the InCheck-DIAL. We as GPs don’t perform that and I’m honest…I know how the device works and I teach it but the average GP doesn’t use the InCheck-DIAL to check whether the inhalation force is correct. That’s something that occurs during GP_A_ consultations of which you hope it occurs. The device is available but I don’t think it’s used in every consultation. They mainly use it when they have doubts or when people have complaints, deposits in the mouth or thrush, to examine how they are inhaling.’’* | 5.1 |
| GP_A1_ | *‘’For a very large group, it doesn’t matter which brand they receive.’’* | 5.1 |
| GP_A1_ | *‘’If it all goes well, they [patients] prefer to stick with the old [device]. When it’s not going well, you immediately have a good reason to say we’re going to try something different but when it’s going well people literally say ‘why should I change? I’m doing fine’.’’* | 5.1 |
| GP_A2_ | *‘’We have an InCheck-DIAL, which is a device that you can use to measure but because of corona it is of course difficult. We do have attachments for it now, but I have to say that I don’t use it that much.’’* | 5.1 |
| GP_A2_ | *‘’It’s easier for starters because they don’t know the difference yet while if they do have one, you often have people who prefer to use their old one.’’* | 5.1; 8.1 |
| CP_6_ | *‘’You have to be cautious with that too, the group of COPD patients also have a very large relatively young category of patients who still work. In research you start with COPD patients from the age of 40 who are still fully engaged in the labor process, so it’s not just 70+ who stay at home. I now also catch myself thinking that a DPI is also easier to carry around for them.’’* | 3.1; 5.1 |
| CP_6_ | *‘’Nowadays there is the InCheck-DIAL so you can measure whether a patient can do it, yes or no? Here in the region you observe that by using the InCheck-DIAL more people are using DPIs because ‘hey, I don’t think that patient can do it’ and with the InCheck-DIAL they notice that someone does have enough strength to use a DPI, so were going for DPIs anyway.’’* | 5.1 |
| CP_1_ | *‘’…That’s only for people who are stable and who are capable…I think those [non-stable] are actually more like to be considered than the stable people.’’* | 5.1 |
| CP_8_ | *‘’I can imagine that a GP, if the patient has sufficient inhalation force, will tend to provide a DPI so that the patient also will have an overview of when it’s empty. I can imagine that.’’* | 5.1 |
| CP_8_ | *‘’If people switch from a pMDI with a spacer to a DPI which may be a discus, they might find that more suitable. So, I can see that happening.’’* | 5.1 |
| CP_8_ | *‘’I think that especially with younger patients, a conversion will be easier than with older patients. It’s dependent on the patient. Suppose you have a patient who has used 10 different devices and is ultimately stable on one, on a pMDI for example, you will of course not convert that to a DPI. So actually, the prior process that took place also plays a role in this [switching].’’* | 5.1 |
| HP_1_ | *‘’There can be different factors why an inhaler works or not and maybe if we think more about sustainability in connection with healthcare, that is a way to improve care and treatment of patients.’’* | 5.1 |
| HP_2_ | *‘’If you can piggyback on other relevant factors, you can also profit, so I agree on that.’’* | 5.1 |
| HP_2_ | *‘’I’m proponent for the 80:20 rule, for 80% of the people it doesn’t matter that much what you give, they all have pretty good molecules. It’s about the 20% or let it be 30%.’’* | 5.1 |
| HP_2_ | *‘’I think 70% can be put onto a DPI, certainly as initial treatment and then it also depends on patient-specific characteristics and especially molecules whether you choose an SMI or DPI.’’* | 5.1 |
| HP_2_ | *‘’I sometimes switch if someone is doing well, that is possible, it depends on the disease status.’’* | 5.1 |
| HP_2_ | *‘’Then you only focus on the effects and not the side-effects, so I think that percentage [70%] is lower…but I think that 50% can be converted.’’* | 5.1 |
| HP_2_ | *‘’I would be an advocate of not switching if someone is doing well because that may involve risks with a lot of time investment. Its better to put the new patients on adequate medication because you have to explain to them anyway, so I would focus more on the starters.’'* | 8.1; 5.1 |
| P_A1_ | *‘’Then this should be examined and discussed to see if there’s something comparable that’s more suitable and also try it out first with the possibility that if it doesn’t work out, I can still go back to the old one, no matter how environmentally unfriendly.’’* | 3.2; 5.1 |
| P_A1_ | *‘’In the past I’ve had all sorts of inhalers that are directly placed on to the mouth. I couldn’t handle that very well in practice. It was really a search.’’* | 5.1 |
| P_A4_ | *‘’I believe the inhaler, the device that contains the medicine, is subordinate to the medicine itself. That it doesn’t really matter in what way you have to inhale as long as it reaches its destination. I don’t think a lot of people think about that at all…that it’s not that relevant to the patient.’’* | 5.1 |
| GP_3_ | *‘’Recently I also provided a training for medical staff and the foundation on sustainability. I would say that the difference between England and Sweden is very big, that one country has 30% pMDIs and the other 70%, there is a whole world of improvement possible in that aspect.’'* | 5.2 |
| GP_3_ | *‘’I think if you choose to contribute to the CO_2_ impact, you should opt for a DPI’’* | 5.2 |
| HP_2_ | *‘’You have to know that this is useful because if you convert everyone now you’ll have succeeded in 5 years but if the carbon footprint of pMDIs is lower than that of DPIs, you’ve done something wrong.’’* | 5.2 |
| **TDF-domain ‘’Beliefs about consequences’’** | | |
| GP_3_ | *‘’A disadvantage is that the industry is supposedly also working very hard to reduce [carbon footprint of pMDI inhalers], so if we wait to long with this project, the CO_2_ will have already been reduced to an extent that it’s [switching] not worth it. That’s the other side of the story.’’* | 6.1 |
| GP_A2_ | *‘’If we want some other device, then it’s: ‘no but the lung specialist said’. So that also plays a role. It’s quite difficult to transfer people once they are already on something else. I understand that, they are used to something and then you receive something new while you actually don’t want that yourself. That’s a hurdle you have to overcome en some just won’t agree to it.’’* | 6.1; (4.2) |
| HP_2_ | *‘’The fact that in two year time the propellants will be much less polluting. That is in 2040 or 2050, then the propellants should give 95% less CO_2_ pollution I believe.’’* | 6.1 |
| HP_2_ | *‘’If I place someone on a pMDI now, I may think ‘oh, oh, oh, that causes problems’ but if in a year and a half that problem is vanished and the patient has been treated well like this, a pMDI might be better.’’* | 6.1 |
| P_A1_ | *‘’I find it difficult because I’ve had a lot of inhalers, including the Ventolin. Well, I can’t tolerate the Ventolin but it’s also the technique of inhaling…I now the use the spacer and personally I find it the most pleasant of all the devices I have used so far.’’* | 6.1 |
| P_A2_ | *‘’The active substance must stay the same but the excipients do not have to be the same. I experienced problems with the excipients. I believe it’s quit risky to experiment with excipients in people who sometimes have an allergic reaction in the lungs.’’* | 6.1 |
| P_A2_ | *‘’We’re not talking about plastic or paper straws. It’s not like ‘oh this feels a little better’. These are people with very sensitive lungs.’’* | 6.1 |
| P_A2_ | *‘’I experience a lot of problems by searching it in this direction. It would be nice but it would be at the expense of health and that should never be the case.’’* | 6.1 |
| P_A3_ | *‘’What also played a huge role for me, why I switched a lot, is that I almost lost my voice and that also has to do with the excipients in inhalers. That’s why I switched 4-5 times until I finally got something that has a good effect and have the least trouble with. That plays a role for me regarding the ultimate inhaler choice’’* | 6.1 |
| P_A4_ | *‘’Unfortunately the interchangeability of medication and inhalers isn’t that good. Pharmaceutical companies all produce their own device with different underlying thoughts, that makes it difficult to exchange them. I’ve also tried various other devices, one didn’t work and the other made me really hoarse, so you can’t just switch.’’* | 6.1; (8.1) |
| P_A4_ | *‘’There are certain conditions in which you don’t want to switch medication. That’s like epilepsy or thyroid hormones, that’s really different from a blood pressure lowering drug, that’s a complete different category in terms of risks. So you would want to continue with medication as stable as possible, that should stay the same and then it isn’t. That’s very worrying, I believe.’’* | 6.1 |
| GP_A1_ | *‘’Sometimes it can be as simple as that they always received a purple inhaler and then suddenly it’s white or pink and they get really upset and don’t use it for weeks until they visit us again.’’* | 6.2 |
| CP_1_ | *‘’The reusable [device] is often very complicated to use…that’s why people visit the pharmacy again, ‘can you push it in, I can’t get it in’. That may not benefit the adherence either.’’* | 6.2 |
| CP_2_ | *‘’At first, we all shifted to pMDIs with spacer, at least here in the pharmacy, because of the better deposition. It feels strange to go to a DPI now due to sustainability or the environment. I can’t let go of that yet, that the pMDI is the preferred device.’’* | 6.2 |
| HP_1_ | *‘’I’ve performed a study a few years ago on medication differences between patients using a DPI or pMDI. We have not found different characteristics between the devices but patients with many treatments, e.g. first a pMDI is chosen and then within a year they are switched to another, have more exacerbations and less controlled disease. So we first examine if the problem is really related to the device or medication or if a higher dosage is warranted before we switch.’’* | 6.2 |
| HP_2_ | *‘’It also depends on the health insurer who makes a very cheap deal regarding a number of devices, converting patients accordingly, which then does not work, causing the patient to have a lung attack, that stuff is thrown away and the patient gets a new one [inhaler] which fortunately does work, but then you’ve had a huge waste in explanation, lung attacks and unusable medication.’’* | 6.2 |
| HP_2_ | *‘’As long as the patient and inhalation problem stays central, that’s fine, but I think we need to discuss it more transcendently otherwise the patient will think the environment or money is the motivation behind it. There is so much distrust in healthcare that I believe we have to be careful.’’* | 6.2 |
| P_A2_ | *‘’I found out afterwards, the dosage, that I was inhaling too much. I thought I had to take half the dosage twice per day of what used to be in it or something. I had switched from 25 to 50 mg and if you keep inhaling the same [frequency], then problems arise.’’* | 6.2 |
| P_A4_ | *‘’It’s important that attention is paid to environmentally friendly options, but ultimately it’s about care.’’* | 6.2 |
| P_A5_ | *‘’I was also admitted to the hospital and that’s when I had enough of it. My lung specialist fortunately said ‘no, just the one you had and no other [inhaler]!’…maybe it’s not related, just a coincidence.’’* | 6.2 |
| P_A5_ | *‘’Environment is important but it also has to work. If someone is taken to hospital by ambulance and is hospitalized for a week, then I think you’ve emitted more CO_2_ than if you just inhaled normally.’’* | 6.2 |
| P_A5_ | *‘’I believe it’s great the environment is taken into account and necessary but I’m afraid we’ll overshoot the target, namely care for our lungs. I hope a balance remains in what’s good for the environment and care for lung patients remains top priority’’* | 6.2 |
| **TDF-domain ‘’Emotion’’** | | |
| P_C1_ | *‘’I don’t use any propellants yet, so I almost feel I’m a ‘clean user’.’’* | 7.1 |
| P_C1_ | *‘’When I hear what you said about the differences…it’s clear what direction to go’’* | 7.1 |
| P_A1_ | *‘’So all new lung patients should be put on DPIs!’’* | 7.1 |
| P_A1_ | *‘’I’m very curious what alternatives are out there instead of pMDIs…whether you’ll receive DPIs or if there are other options.’’* | 7.1 |
| CP_1_ | *‘’There’s a ‘responsible exchange’ list which contains inhalers that shouldn’t be exchanged but somehow that list is of secondary importance to health insurers and preference policies…In principle we should not worry about the preference policy for inhalers at all, because they are all on the list.’’* | 7.2 |
| CP_6_ | *‘‘It may also be a fear that you think the patient does not have enough strength and it [DPI] will not reach its target site.’’* | 7.2 |
| CP_7_ | *‘’If a doctor prescribes a DPI, they have to choose between a Ventolin discus or salbutamol Novolizer for example but it doesn’t force you to switch the patient to a pMDI. Not yet, they are not there yet but you never know, it could happen in the future.’’* | 7.2 |
| P_A1_ | *‘’Well, shocking! [environmental impact]’’* | 7.2 |
| P_A1_ | *‘’I would find it [inhaler switch] a scary step to take.’’* | 7.2 |
| P_A2_ | *‘’I’ve never heard of it going wrong, maybe this is my imagination but sometimes I find it [switch] a scary idea.’’* | 7.2 |
| P_A3_ | *‘’Recently I read an article in the newspaper about inhalers in relation to the carbon footprint, I was slightly shocked by it. If that’s what you mean by climate-friendly, then I get it!’’* | 7.2 |
| P_A4_ | *‘’Health insurers are of course also focusing on this. I absolutely don’t want them [health insurers] to determine that I must have a certain inhaler if it doesn’t work for me. That you do retain freedom of choice…it’s a delicate matter.’’* | 7.2; (3.3) |
| P_A1_ | *‘’I find it difficult because I’ve also used those DPIs and that didn’t go well so I’m like ‘not with my body’ but then I hear these numbers then perhaps I need to start looking at DPIs at some point’’* | 7.3 |
| **OPPORTUNITY** | | |
| **TDF-domain ‘’Environmental context and resources’’** | | |
| GP_1_ | *‘’I’m not familiar with that problem [influence of preference policy] because the local formulary is signed by … [health insurer], that’s very pleasant.’’* | 8.1 |
| GP_2_ | *‘’The use of multiple devices is sometimes the reason why I choose something else.’’* | 8.1 |
| GP_2_ | *‘’We have a local formulary, this is agreed on between the hospitals and GPs so that they more or less prescribe medication in the same manner. You can choose between different DPIs or pMDIs but you only have one or two options in each group, who more are less are of the same type so that you don’t have a discus for one and something completely different for the other. That is complicated for people. The difficult part is that health insurers do not always accept that, so then it’s not reimbursed.’’* | 8.1 |
| GP_2_ | *‘’Medication is often not available…that is very difficult…then you shift from a discus to Novolizer or something else. That may be converted at the pharmacy without my knowledge and then the patient is sent home with a complete different device and inhalation technique.’’* | 8.1 |
| GP_3_ | *‘’Starting a Novolizer is nice but the DPI does not contain the combination medicine, so you often run into the problem that the ‘series’ isn’t complete’’* | 8.1 |
| GP_3_ | *‘’You try to stay in a series [of devices]…the tricky part with the Novolizer, that’s why it’s not for asthma, is that you do not have the combination medication in there.’’* | 8.1 |
| GP_3_ | *‘’You want the entire series [of medication] to be in the same device.’’* | 8.1 |
| GP_3_ | *‘’The preference policy also determines in the end. So for the Symbicort I get something different and the Foster is a pMDI while those contain the two preferred medicines according to the new asthma guidelines…so you are really hindered there.’’* | 8.1 |
| GP_3_ | *‘’Their formulary first examines which health insurer is involved and then they choose the inhaler device. We were wondering if that’s the way to go. Our formulary is based on an ideal world but the problem is we don’t have an ideal world….then it turns out that the Seretide is replaced, Symbicort is replaced…you’re very dependent on the health insurer.’’* | 8.1 |
| GP_3_ | *‘’[health insurer] has the most influence as this where the majority of poor people are insured, this also contains the group of COPD patients. COPD is a disease of the lower social classes, people with a lower income and a greater smoking history. That makes it extra complicated for us.’’* | 8.1 |
| GP_A1_ | *‘’A device in which multiple options are available is preferred. So, if people need multiple devices that they can use the same sort of device. With a pMDI that does not matter that much, a pMDI is a pMDI.’’* | 8.1 |
| GP_A1_ | *‘’It would be nice if a patient can get the same brand device, so that they don’t get a different color or name every three months, but that can’t be arranged.’’* | 8.1 |
| GP_A2_ | *‘’People are used to something and then they suddenly get another. It’s often the health insurance that determines this, people find that very annoying and some have no confidence in it. It’s very difficult for people to gain confidence in it.’’* | 8.1 |
| GP_A2_ | *‘’The health insurers actually have a lot of influence. The insurer causes people to be switched to other things than that we have prescribed, that happens a lot. We have established a regional formulary but in the end the insurer determines. They don’t take it seriously. They say ‘yes’ but then they don’t cooperate, that remains very difficult.’’* | 8.1 |
| CP_1_ | *‘’So in our region people are often switched to pMDIs, then you at least have freedom of choice with a spacer and get a high quality product. That may not be good for the environment, that’s secondary.’’* | 8.1 |
| CP_1_ | *‘’The lung formulary often conflicts with national preference policies of different health insurers. I’m curious what will come out of this as it’s becoming increasingly complicated to combine everything.’’* | 8.1 |
| CP_1_ | *‘’The DPI such as the discus consisted of one action. When the discus lost its patent, you had to pull a kind of strip before inhaling was possible, so a whole new action was introduced. If you didn’t pull the strip, you simply inhaled an empty strip from the day before. If you don’t realize that, it goes wrong. So, we come up with a name of a medication we want to prescribe and okay this lady can handle a DPI but then you receive a bad product and pharmacies deal with it differently. One will say I won’t deliver this and the other will say I have to comply to the preference policy otherwise I will be short-changed’’* | 3.1; 8.1 |
| CP_1_ | *‘’There aren’t that many SMIs available yet, right?’’* | 8.1 |
| CP_1_ | *‘’The availability problems and preference policy make it complicated at the moment, there are so many factors at play right now instead of sustainability. If all those other factors were not at play, sustainability in itself would already be complicated to implement because you still have to look at patient factors. So, I sometimes end up with a pMDI because of all those other influencing factors. Therefore, my attention is not focused on that [sustainability] at all right now. That’s not because I don’t believe it’s important but because other aspects require more attention. If maybe one of these aspects I experience would be resolved, then I can fit sustainability into it.’’* | 8.1 |
| CP_1_ | *‘’When New Years Eve has passed and health insurers make other devices preferent, you can receive a DPI in box A in December and a completely different DPI in January. That’s not the case with pMDIs, you can receive a different pMDI in a different color maybe, but you attach it to the same spacer, the procedure remains the same. You have a more stable set of preparation steps before inhaling.’’* | 8.1 |
| CP_2_ | *‘’I agree on the influence of the preference policy and the lung formulary, the same problems here.’’* | 8.1 |
| CP_3_ | *‘’Low literacy also plays a major role. That causes problems with instructions and which inhaler best to choose. It causes a lot of challenges, especially in the area of communication’’* | 8.1 |
| CP_3_ | *‘’This [preference policy] limit sour freedom of choice and that of the prescriber.’’* | 8.1 |
| CP_4_ | *‘’The existence of a formulary to reach the most suitable medication for a patient is not the problem, but rather the preference policy.’’* | 8.1 |
| CP_5_ | *‘’We encounter issues with the preference policy in combination with the formulary in practice, which often clash with each other.’’* | 8.1 |
| CP_6_ | *‘’The preference policy what my colleagues mentioned. We also encounter that in this region.’’* | 8.1 |
| CP_6_ | *‘’Sometimes I want to choose an inhaler that’s not preferred, as a pharmacist I’m then held accountable.’’* | 8.1 |
| CP_6_ | *‘’You may also want to choose medication which is not available in a pMDI.’’* | 8.1 |
| CP_6_ | *‘’It limits your choice but not whether or not someone is allowed to have a DPI. The health insurer does not force me to choose a pMDI but it does force me to choose between different DPIs. Salbutamol is a good example, known as Ventolin in the old days. Now health insurers prefer the Novolizer. That’s another device with a slightly different resistance. That’s where the preference policy interferes.’’* | 8.1 |
| CP_6_ | *‘’What medicines are available in that particular inhaler device…’’* | 8.1 |
| CP_7_ | *‘’We do insist as there’re financial consequences for the pharmacy. GPs must take that into account.’’* | 8.1 |
| CP_7_ | *‘’In the way it’s arranged now, I don’t know, then we have to organize the pharmacy and manage staff as it will take longer because you’ll have to measure how much inhalation force someone has. It has to be set-up in such a way that we can perform it properly. Now the GP_A_ does that and we provide inhalation instructions. Though, it would be a nice to implement in the pharmacy as well.’’* | 8.1 |
| CP_7_ | *‘’Then you’ll indeed also have to train your staff to perform this properly.’’* | 8.1 |
| CP_8_ | *‘’…the language barrier, both written and spoken, that is still a challenge. We usually spend more time at the counter communicating a simple story as when your in a district with younger people or people who do speak the language sufficiently. That’s always a challenge, but also makes it fun to work there.’’* | 8.1 |
| CP_8_ | *‘’That [preference policy] is an issue…GP_A_ prescribe according to the formulary. The preference policy then says something different, it also changes every year, so that’s an issue.’’* | 8.1 |
| CP_8_ | *‘’In the beginning of last year we had some discussion, because the GPs prescribe something according to the lung formulary but then we convert it because of the preference policy. The GP argues that the lung formulary is valid so why is the preference policy added? Everyone points to each other.’’* | 8.1 |
| CP_8_ | *‘’Then you’re moving towards a consult, you really have to organize everything, not only the location but also your time and staff’’* | 8.1 |
| HP_2_ | *’I really don’t have time for it [discuss environmental impact], I’m happy if I can treat patients adequately. Also taking the world issues into account, that’s something that wouldn’t work’’* | 8.1 |
| HP_2_ | *‘’Converting to a SMI in someone who needs an ICS, still leads to a pMDI. That does not make sense unless it’s required for other reasons. If someone only needs an SMI, a LABA/LAMA, that’s fine, but also supervised.’’* | 8.1; 2.2 |
| P_A5_ | *‘’I received the supposedly same active ingredient but in different proportions from Poland but the dose-counter got really whack. I pressed it once and it immediately showed 20 while I thought there was a lot more in it…I didn’t find that reliable.’’* | 8.1 |
| GP_1_ | *‘’We have a beautiful poster that we get from the care group on how we should prescribe but the word sustainability is not mentioned anywhere…and this is from 2022 so that still requires some attention. Oh, there is a very small thing placed on it. That’s funny, I only just noticed it for the first time, it’s very inconspicuous. ‘The Green GP says you should preferably not use pMDIs’.’’* | 8.2 |
| GP_1_ | *‘’I believe the implementation requires relatively simple and quick process changes, so a pMDI yes or no? That’s easy to remember, implement and communicate.* | 8.2 |
| GP_1_ | *‘’I believe that’s a task for the Nederlands Huisartsen Genootschap (NHG). They have included a section on sustainability in their policy plan for 2023-2025 but that has not yet been worked out yet. I believe it’s necessary to publish an article about it in ‘huisarts en wetenschap’ as incentive, not just on this topic but also on what choices can be made.’’* | 8.2 |
| GP_1_ | *‘’I don’t think these [formulary] are put together based on sustainability.’’* | 8.2 |
| GP_3_ | *‘’…I asked to add more DPI options to the formulary because Foster, as it’s a single device, isn’t included at the moment...but because we have so much Foster in the guidelines with the SMART method, that should also be possible as DPI. I asked if we could look at that during the next revision.’’* | 8.2 |
| GP_3_ | *‘’No, there is no brochure about that [sustainability of inhalers].’’* | 8.2 |
| GP_A1_ | *‘’A shortcut could be made if you can find out which inhaler is the most and least environmental harmful, then that could be added to the formulary e.g. the provision of a number classification behind each inhaler. That one is very environmentally friendly and 10 is not at all. That we can take that into account, if we are in doubt between two sorts, that we choose the most sustainable. I believe that would be the most practical for us in order to make better choices.’’* | 8.2 |
| CP_1_ | *‘’You comply with an agreement but if it fails somewhere, the entire car comes to a standstill. That’s what I notice with the current formulary.’’* | 8.2 |
| CP_1_ | *‘’In itself the formulary is very good and we do our best to adhere to it but it’s difficult to maintain.’’* | 8.2 |
| CP_2_ | *‘’The formulary is the holy grail. I get the impression that it has been thought through very well by specialists, GPs and CPs. That’s what we all work with.’’* | 8.2 |
| CP_3_ | *‘’I believe there was something about that in the green GP practice booklet.’’* | 8.2 |
| CP_3_ | *‘’the formulary is useful for all prescribers who enter the profession. If they are taught well, they will start sooner with DPIs’’* | 8.2 |
| CP_4_ | *‘’I find the formulary useful because all healthcare professionals came together and made agreements that are in accordance with guidelines and that you can provide the most suitable device for each individual within a limited range of possibilities.’’* | 8.2 |
| CP_8_ | *‘’We use inhalatorgebruik.nl [link to educational patient material] standardly.’’* | 8.2 |
| HP_2_ | *‘’I would strongly argue in favor of expanding and professionalizing inhalatorgebruik.nl [link to educational patient material]…It could include a paragraph or video on sustainability as ultimately the patient is the end-user, if they realize the footprint, it can help’’* | 8.2 |
| HP_2_ | *‘’There are companies advertising their product is the best, I find that worrying, we don’t know that exactly. Educational material can only be created if you can actually say something sensible about it.’’* | 8.2 |
| P_A4_ | *‘’There are videos, inhalation instruction videos, that can be watched and then the hope is that you can do it just as well yourself.’’* | 8.2 |

*1.1 = Knowledge and awareness on environmental impact of inhalers; 1.2 = Multitude of environmental elements; 1.3 = Insufficient overview of critical information; 2.1 = Inhaler device ability and competence differences; 2.2 = Inhaler skill development, practice and assessment; 3.1 = Current environmental-friendly inhaler decision influences; 3.2 = Importance of SDM; 3.3 = Prioritization of environmental-friendly inhaler decision-making; 4.1 = Environmental responsibility; 4.2 = Potential organizational roles; 5.1 = Perceived competence to switch to environmental-friendly alternatives; 5.2 = Confidence to combat climate change; 6.1 = Anticipated regret to prioritize environmental-friendly alternatives; 6.2 = Poor outcome expectancies of non-medical inhaler switching; 7.1 = Positive association; 7.2 = Negative association; 7.3 = Cognitive dissonance; 8.1 = (Potential) environmental stressors; 8.2 = (Material) resources.

*Abbreviations: COM-B: Capability, Opportunity, Motivation, Behavior; COPD: Chronic Obstructive Pulmonary Disease; DPI: dry powder inhaler; CP: community pharmacist; GP_(A)_: general practitioner (assistant); GWP: global warming potential; HCP: healthcare professional; HFC: hydrofluorocarbon; HP: hospital pulmonologist; P_A/C_: asthma/COPD patient; pMDI: pressurized metered dose inhaler; PTAM: pharmacotherapeutic audit meeting; PW: Pharmaceutisch Weekblad (journal of the Royal Dutch Pharmacists Association); SDM: Shared Decision Making; SMART: Single Maintenance And Reliever Therapy; SMI: soft mist inhaler; TDF: Theoretical Domains Framework.*

**Table E.2 Perceptions on potential implementation strategies to factor environmental impact into inhaler treatment decision-making identified in focus group discussions, organized into identified action areas and constructs.**

| **Participant** | **Quotes** | **Construct*** |
| --- | --- | --- |
| **ACTION AREA 1: Communication, education and awareness** | |  |
| GP_1_ | *‘’Next week I’m organizing a training on sustainability, a PTAM on sustainability in our practice.’’* | 1.1 |
| GP_1_ | *‘’PTAM, that’s a group of CPs and GPs together, and then we choose topics. The one who chooses a topic also has to prepare it. So we’re going to focus on possibilities in the practice, also on medication use. We examined the percentage of pMDI users in our practice and if there is something we can do about that? We’ll discuss that with each other and then we’re going to make a plan for everyone who wants to join, who wants to start with it…I know that 39% of patients receive a pMDI here, we’ve a list from above 7 [years], that’s quite a large group. We can examine that specifically as not many have done anything yet.’’* | 1.1; 1.2 |
| CP_3_ | *‘’Of course we’ll consider what you’ve presented here in the PTAM and better inform doctors about the options available and not to choose for pMDIs so easily’’* | 1.1 |
| CP_7_ | *‘’We happen to have a PTAM on asthma and COPD in June, then I will ask them more about how they make their choice.’’* | 1.1 |
| CP_7_ | *‘’The first step is to make good agreements with doctors but also to discuss the sustainability aspect with them and show numbers so that instead of choosing a pMDI standardly, they also consciously think about starting with a DPI if the patient can handle it. They way the healthcare is currently organized, the first step is to make agreements with GP_(A)_.’’* | 1.1; 1.2 |
| HP_2_ | *‘’We could position that [link to educational patient material] even stronger, also when prescribing medication. I always have a reference on my prescription’’* | 1.1 |
| P_A4_ | *‘’You first have to generate some support’’* | 1.1 |
| CP_8_ | *‘’This has to be performed regional or even smaller, making those agreements with each other. It would be nice if the regional agreements regarding the devices are also respected by the insurers.’’* | 1.2 |
| CP_8_ | *‘’That healthcare professionals have examined together what is best for the patient and then you could also incorporate the sustainability aspect in terms of what’s recommended as first choice. If that is then respected by the health insurers, you’re there.’’* | 1.2 |
| GP_2_ | *‘’If we want to find out the status of things, indeed overuse of salbutamol, then I ask the pharmacy for a printout of patients who use too much’’* | 1.3 |
| GP_2_ | *‘’They can be identified and then you select them. The assistant then calls to see if they’re okay with that [switch to DPI] and then you convert them.’’* | 1.3 |
| CP_6_ | *‘’This contains some investment to keep it going and also to go back to your doctors every now and then, ‘hey, listen, we agreed this and that’. That also depends on your PTAM structure’’* | 1.3 |
| **ACTION AREA 2: Appropriate inhaler prescribing** | |  |
| GP_1_ | *‘’Mainly leaving things, so not doing things, that is the most important, so not providing medication if it’s not strictly necessary.’’* | 2.1 |
| GP_1_ | *‘’It’s also about the theme doing or not doing, that’s not only sensible care but also sustainable care. So we make that a topic of discussion with each other.’’* | 2.1 |
| GP_3_ | *‘’It’s most sustainable to not prescribe medication then it will not pollute the water. There are of course a number aspects you can do with responsible prescribing, not prescribing anything is a lot cheaper, no painkillers, no antidepressants, then they do not pollute the water either.’’* | 2.1 |
| GP_A1_ | *‘’In COPD it hasn’t been proven that ICS helps. The assignment was to stop Seretide in COPD patients and only provide a bronchodilator. We did that in the majority of patients. A few patients did not want to switch, so I left it. While some who switched got more exacerbations…maybe it has not been proven, but for this patient it apparently worked…So I find that difficult’’* | 2.1 |
| CP_3_ | *‘’People often travel or want an inhaler multiple locations…’’* | 2.1 |
| CP_7_ | *‘’I believe in optimal use and phasing-out of medicine when necessary so people do not stay on inhalers for an unnecessarily long time’’* | 2.1; 4.1; 4.2 |
| CP_7_ | *‘’Those ICS, which can be reduced at a certain point in COPD, that should e.g. be closely monitored’’* | 2.1 |
| **ACTION AREA 3: Promote smarter inhaler choices** | |  |
| GP_1_ | *‘’Focus on lifestyle, prescribing small amounts and especially not to prescribe pMDIs, those are actually key points for me’’* | 3.1; 4.1 |
| GP_2_ | *‘’When it comes to sustainability, I think that if you prescribe something, then consider what kind of medication to prescribe. We have been focusing on pMDIs and DPIs for some time now, so regarding sustainability those pMDIs are more burdensome’’* | 3.1 |
| GP_2_ | *‘’We’ve been busy in our care group to reduce pMDIs. We’ve chosen, I believe, age >10 years and maybe <70 or 75 years to start converting those people and consciously explained to patients it’s because of the environment.’’* | 3.1 |
| GP_A1_ | *‘’Avoid disposable inhalers as much as possible…and the pMDIs with propellant in it.’’* | 3.1; 3.2 |
| GP_A1_ | *‘’If it all goes well, they [patients] prefer to stick with the old [device]. When it’s not going well, you immediately have a good reason to say we’re going to try something different but when it’s going well people literally say ‘why should I change? I’m doing fine’.’’* | 3.1 |
| GP_A2_ | *‘’For starters it's easier as people don’t know the difference yet, while if you already have one you often have people who prefer their previous device.’’* | 3.1 |
| GP_1_ | *‘’You prefer to have a minimum of different things [devices].’’* | 3.2 |
| GP_2_ | *‘’We try to stay within the same device as much as possible, it that’s within the prescription options, because then they don’t have to learn anything new.’’* | 3.2 |
| GP_A1_ | *‘’You detect it in their medication dispensing overview that it has been repeated to late: ‘that can’t be that it has been used twice a day, because then it would have been empty three months ago’. But often when you ask about it, they also indicate themselves ‘I actually forgot it in the evening’. Then you can better switch to e.g. Relvar or something that works for a duration of 24 hours.’’* | 3.2 |
| GP_A1_ | *‘’If they use multiple inhalers, that they get the same type, so they don’t have a pMDI, SMI and DPI, everything mixed together’’* | 3.2 |
| GP_A2_ | *‘’For the people who do not faithfully inhale their inhaler twice a day but with difficulty can manage it once daily, a 24-hour inhaler can be chosen, which can be used once daily.’’* | 3.2 |
| CP_4_ | *‘’We might also have to think about re-using devices but that also has to remain convenient for the patient, that they get it in easily’’* | 3.2 |
| CP_6_ | *‘’Dosing once or max twice daily, that’s also something I think is important, also to ensure people use it’’* | 3.2 |
| CP_8_ | *‘’If patients use several inhalers, a combination inhaler is often a solution, increasing patient compliance’’* | 3.2 |
| P_C1_ | *‘’Or another type of propellant?’’* | 3.2 |
| P_C2_ | *‘’A system where you can detach it and put a new disk in separately. Then at least the disk itself can last longer and does not have to be replaced that often.’’* | 3.2 |
| HP_2_ | *‘’…Dosing frequency, as the more you use, the more...with the lowest possible dosage as why use more medication if it isn’t necessary?’’* | 3.2 |
| HP_2_ | *‘’There’s reduce and re-use and so on, first we have to make sure that it’s as little as possible and that what we use is re-used.’’* | 3.2 |
| **ACTION AREA 4: Optimization of quality of care** | |  |
| GP_3_ | *’Underuse, which you can detect well by what has actually been collected in the pharmacy in the past period and sometimes it’s also an incorrect inhalation technique. Aspects that all have to be checked’’* | 4.1 |
| GP_A1_ | *‘’Maybe they [smokers] are more adherent to therapy because they are more short of breath in general. But then again some smokers are not because there’s also a large group of smokers who say: ‘I don’t use my inhaler because I smoke too’. It’s like they don’t deserve to use their inhaler.’’* | 4.1 |
| GP_A2_ | *‘’We know that ICS work less well in people who smoke.’’* | 4.1 |
| GP_A2_ | *‘’Always ask why they use something or not and how often. A lot goes wrong in that area, it’s important to get a good view of why it does not work or people don’t do that’’* | 4.1 |
| GP_A2_ | *‘’You have to ask what the problem is. Is it their own stubbornness? Are they afraid? That remains the most important part.’’* | 4.1 |
| CP_1_ | *‘’The number of exacerbations someone has and use of SABA. If that’s high and they also had a prednisone course, those are people where at least the inhalation technique should be re-evaluated. Perhaps you’ll come to the conclusion to switch to a different device’’* | 4.1 |
| CP_5_ | *‘’I believe the lung care has also been neglected here, especially after corona. From within the pharmacy but also from GPAs and GPs, less attention has been paid, while we know that therapy adherence and use of inhaler medication often goes wrong or certainly requires attention.’’* | 4.1 |
| HP_2_ | *‘’My focus would be on adherence, that’s the most important part…’’* | 4.1 |
| HP_2_ | *‘’The use of medication, whether patients use it faithfully or not as that’s where the effectiveness lies. If you have really good medication but it’s not used because the packaging is inconvenient, then it’s also thrown away and not effective.’’* | 4.1 |
| P_A3_ | *‘’With the last few [dosages] you know that there’s hardly any effective substance left, so at some point I will throw it away.’’* | 4.1 |
| CP_1_ | *‘’Pulmonary nurses talk a lot about the one-breath method, especially with the spacer…but you actually shouldn’t do that with pMDIs’’* | 4.2 |
| GP_2_ | *‘’Of course I’ve seen them all [inhaler products] at some point but the last IMIS I did was probably three years ago. In the meantime a lot of new devices have been released’’* | 4.2 |
| GP_A1_ | *‘’’Those puffs [pMDIs] don’t help at all and I do it every day. I put it on the chimney and then I turn it on and it sprays into the air.’ He literally placed the inhaler in the middle of the room and thought I’ll breathe that in or something. I had really given an instruction but it didn’t come through.’’* | 4.2 |
| HP_2_ | *‘’I believe we can gain a lot with inhalation instructions and adequate use’’* | 4.1; 4.2 |
| HP_2_ | *‘’Our lung consultants say ‘anyone can learn a SMI’. It depends on the quality of the instructor and not on the patient but you really have to invest in it. So its [SMI] more difficult because it requires a bit more explanation…’’* | 4.2 |
| **ACTION AREA 5: Appropriate inhaler disposal** | |  |
| GP_1_ | *‘’I believe that’s the pharmacist’ job, that’s where they receive medication and where the boxes stand to hand it in. We shouldn’t be doing that ourselves.’’* | 5.1 |
| CP_7_ | *‘’That they [inhalers] can be handed in at the pharmacy as now we throw it in one pile of chemical waste’’* | 5.1; 5.2 |
| HP_2_ | *‘’We must support patients to not throw away their DPIs plastic but to dispose of it.’’* | 5.1 |
| CP_7_ | *‘’I believe the manufacturer can play a role in terms of making sure it’s made from recycled plastic…maybe special recycling bins can separate inhalers.’’* | 5.2 |
| P_A3_ | *‘’There’s a lot to be gained in terms of material, that’s industrial design and I’ve no idea how to replace plastic…but suppose you’re to make a mountain of all inhalers used in a year, how much plastic that would be.’’* | 5.2 |

*1.1 = Relationship building and educational efforts on topic; 1.2 = A collective environmental-friendly action plan; 1.3 = Cyclic monitoring of environmental-friendly actions; 2.1 = Reduce unnecessary inhaler use; 3.1 = Prioritize DPIs as environmental-friendly inhaler *(if appropriate)*; 3.2 = Consider a sustainable device design and dosing treatment regimen *(if appropriate)*; 4.1 = Support individualized self-management of inhaler use in patients; 4.2 = Regular assessment and ongoing education on correct inhaler technique; 5.1 = Return inhalers to the community pharmacy; 5.2 = Set-up of inhaler recycling scheme.

*Abbreviations: BA: breath-actuated; COM-B: Capability, Opportunity, Motivation, Behavior; COPD: Chronic Obstructive Pulmonary Disease; DPI: dry powder inhaler; CP: community pharmacist; GP_(A)_: general practitioner (assistant); GWP: global warming potential; HCP: healthcare professional; HFC: hydrofluorocarbon; HP: hospital pulmonologist; P_A/C_; asthma/COPD patient; pMDI: pressurized metered dose inhaler; SDM: Shared Decision Making; SMART: Single Maintenance And Reliever Therapy; SMI: soft mist inhaler; TDF: Theoretical Domains Framework.*
